# Prediction of Nanoparticle Sizes for Arbitrary Methacrylates Using Artificial Neuronal Networks

**DOI:** 10.1002/advs.202102429

**Published:** 2021-10-23

**Authors:** Julian Kimmig, Timo Schuett, Antje Vollrath, Stefan Zechel, Ulrich S. Schubert

**Affiliations:** ^1^ Laboratory of Organic and Macromolecular Chemistry (IOMC) Friedrich Schiller University Jena Humboldtstr. 10 Jena 07743 Germany; ^2^ Jena Center of Soft Matter (JCSM) Friedrich Schiller University Jena Philosophenweg 7 Jena 07743 Germany

**Keywords:** drug delivery, graph convolutional network, machine learning, nanoparticles, nanoparticle size, neuronal network

## Abstract

Particle sizes represent one of the key factors influencing the usability and specific targeting of nanoparticles in medical applications such as vectors for drug or gene therapy. A multi‐layered graph convolutional network combined with a fully connected neuronal network is presented for the prediction of the size of nanoparticles based only on the polymer structure, the degree of polymerization, and the formulation parameters. The model is capable of predicting particle sizes obtained by nanoprecipitation of different poly(methacrylates). This includes polymers the network has not been trained with, indicating the high potential for generalizability of the model. By utilizing this model, a significant amount of time and resources can be saved in formulation optimization without extensive primary testing of material properties.

## Graphical abstract

Utilizing a combination of different model structures and machine learning techniques, a model that is able to predict the size of polymeric nanoparticles based solemnly on the structure and preparation parameters is presented. The prediction achieves results with errors <10%, even for previously unseen structures, which demonstrates the generalizability of the model.

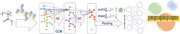

## 1 Introduction

Nanoparticles represent a crucial enabler of modern nanotechnology and are highly relevant in many different applications. Catalytic systems,^[^ ^1^, ^2^ ^]^ sensors,^[^ ^3^ ^]^ and biomedical applications^[^ ^4^ ^]^ are significantly improved during the last years by applying nanoparticle technology to obtain tailor‐made properties. In particular, polymer‐based nanoparticles feature the great benefit of a broad structural diversity and a relative chemical inertness, which are beneficial for, for example, biomedical approaches^[^ ^5^ ^]^ such as drug^[^ ^6^, ^7^ ^]^ or gene delivery.^[^ ^8^, ^9^ ^]^ Furthermore, the properties of such systems can easily be varied using different monomers, polymer compositions, or additives.^[^ ^10^ ^]^ Consequently, polymeric nanoparticles can be designed in a tailor‐made manner for a specific task, for example, the targeting of certain organs or tumors in the human body, which can lead to a highly selective drug‐delivery and, thus, a reduction of potential side‐effects.^[^ ^11^ ^]^

One of the most critical parameters determining the properties of polymeric nanoparticles, besides the structural composition, is the particle size. It defines the volume‐to‐surface ratio, the properties such as cellular uptake efficiency,^[^ ^12^, ^13^, ^14^ ^]^ the loading capacity,^[^ ^15^ ^]^ the renal clearance,^[^ ^16^ ^]^ and, consequently, influences the targeting efficiency for specific organs as well as the overall toxicity.^[^ ^9^ ^]^ However, the size strongly depends on many different parameters, such as the type of polymer, the molar mass, or the preparation technique. Therefore, the development of a method for the preparation of nanoparticles with defined particle sizes is highly challenging, and many attempts in a trial‐and‐error approach must be performed in order to obtain the nanoparticles of the desired size. This multidimensional problem is predestined to be tackled using machine learning approaches, which can, in theory, fit any function and, as a result, predict highly complex relationships.^[^ ^17^ ^]^ The trend to perform chemical in silico calculations for the prediction of molecular and material properties shifted in recent years from methods like density functional theory (DFT) or other ab initio methods to data‐driven methods from the field of machine learning.^[^ ^18^ ^]^ One of the main reasons is that once the model is trained, which might take a very long time, each prediction takes a fraction of the time of classical calculations (e.g., DFT), which are then much more computation‐intensive.^[^ ^19^ ^]^

Based on this challenge, the current study focuses on a machine‐learning approach for the size prediction of polymeric nanoparticles based on poly(methacrylates), which represents an often‐utilized polymer class for nanoparticle preparation.^[^ ^20^, ^21^, ^22^ ^]^ Some first attempts regarding this challenge were already published using neural networks.^[^ ^23^, ^24^, ^25^, ^26^, ^27^ ^]^ As shown, such machine learning techniques can feature a high benefit for drug delivery because of the high multidimensionality factors influencing a successful system.^[^ ^28^ ^]^ However, all of the available models feature significant drawbacks, such as a limitation to a specific polymer^[^ ^24^ ^]^ or a material‐composition system^[^ ^23^ ^]^ and the need for distinct physical properties as input data,^[^ ^27^ ^]^ which have to be determined with time and cost‐intensive methods. Additionally, a reoccurring deficit in previous publications is the fact that the models are often trained only using very small datasets (usually below 50 samples). The resulting models, even if not overfitted, may only predict properties within the space of training data with sufficient accuracy and, thus, cannot be generalized to other structures or compositions.

The herein presented approach focuses on using mainly the chemical structure as input data and, thereby, a machine‐learning technique, using the approach of a chemist: looking at the structure and predicting the properties. Such an approach was implemented for the first time in the prediction of nanoparticle sizes and, to the best of our knowledge, for polymers in general. This more general method enables the model to predict particle sizes for any polymer regardless of the polymer type or derivatization. This makes this model extremely useful for the preparation of nanoparticles of new polymers since the preparation parameters can be tailored to the desired size. As a result, the number of experiments and, therefore the expected time and resources required, can be reduced to a small fraction of the classical approach.

## 2 Results and Discussion

### 2.1 Model Design

**Figure 1:**
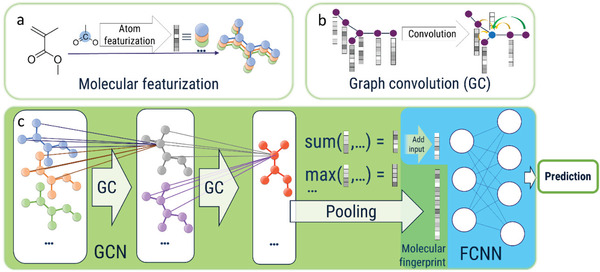
Schematic representation of the graphical overview of the model workflow. First, the structural data are transformed into a node‐featurized graph representation (a), which is subsequently fed into a multi‐layered graph convolutional network (GCN). In each layer, the features of each node are updated with a new feature vector created by applying a convolutional kernel on the feature vectors of the connected nodes (b). After pooling over the whole graph via different pooling functions, the resulting fixed size vectors are passed, together with additional input data, into a fully connected neuronal network, which regresses the desired tasks (e.g., particle size) (c).

For an efficient prediction, a model was designed, which can be split into two general parts: a graph convolutional network (GCN) and a fully connected neuronal network (FCNN) (**Figure** **1**). The GCN is utilized for the interpretation of the (chemical) structural information and for the reduction to a fixed size vector, which can be applied within the FCNN. Usually, a GCN is utilized as a standalone model for the prediction of molecular properties, for example, for their potential application as drugs^[^ ^29^ ^]^ or for reactivity prediction.^[^ ^30^ ^]^ This works very well for properties that are only derived from the structural input. However, for the current investigation, this is not applicable since additional information, such as formulation parameters and degree of polymerization, is required for a sufficient accuracy of the prediction.

As input, the GCN takes a node‐featurized graph representation of the chemical structure. Hereby, a graph *G* = (*N*, *E*) is defined by its node set *N* = {*n*_1_,*n*_2_,…, *n_n_*} and edges set *E* = {(*n_i_*,*n_j_*) *n_i_*,*n_j_* ∈ *N*}, representing the atoms |*N*| = |*atoms*|, which are connected via edges, representing the bonds |*E*| = |*bonds*|. Each node becomes featurized with an atom specific feature vector *n_n_* = *F^n^*, which is derived from the underlying atomic properties $F_{i}^{n}=f_{i}\;\left(atom_{n}\right)$. Herein, *f_i_* is a featurizing function, for example, the type of atom, (partial) charge, or hybridization (for a complete list as well as an exemplary vector for methyl methacrylate, see Supporting Information). The edges stay featureless and only define the relationships between the nodes. In each layer of the GCN, a new node‐feature‐vector is calculated via a weighted‐sum of each feature‐vector of each connected node and the feature‐vector itself, which can be calculated as a feature matrix for the whole graph in one expression:

(1) $$H_{n+1}=\;\sigma \left({\tilde{D}}^{-\frac{1}{2}}\tilde{A}{\tilde{D}}^{-\frac{1}{2}}H_{n}W_{n}+b_{n}\right)$$

Hereby, *H_n_* represents the feature matrix representation of the n^th^ layer of the GCN, with *H*_0_ being the feature matrix of the initial graph (*H*_0_ ∈ *M*_|*N*| × |*F*|_), *σ* the activation function, *W_n_* and *b_n_* represent the trainable weight matrix and the bias of the n^th^ layer, $\tilde{A}$ and $\tilde{D}$ the adjacency matrix and the degree matrix of the graph, including the identity matrix, to assure self‐awareness of each node.^[^ ^31^ ^]^ Another way to comprehense the graph convolution is that in each layer, each node sends its information to all connected nodes. Each node then collects all messages it receives and combines them with its own feature vector to a new feature vector, thus, also the name message passing neuronal network (MPNN).^[^ ^32^ ^]^

Since each layer represents a convolution of all directly connected nodes, the awareness range of each node and, thus, of the network is equal to the number of convolutional layers in the model. The number of layers, as well as the feature vector size after each layer, is a tunable hyperparameter. The output of the graph convolution is a graph with the same topology as the original input graph with convoluted feature‐vectors. The GCN is connected with the fully connected neuronal network (FCNN) by applying pooling functions (max and weighted‐sum) to all feature‐vectors of the graph, and the resulting vectors are passed as a fixed size concatenated vector into the FCNN (Figure 1).

**Figure 2:**
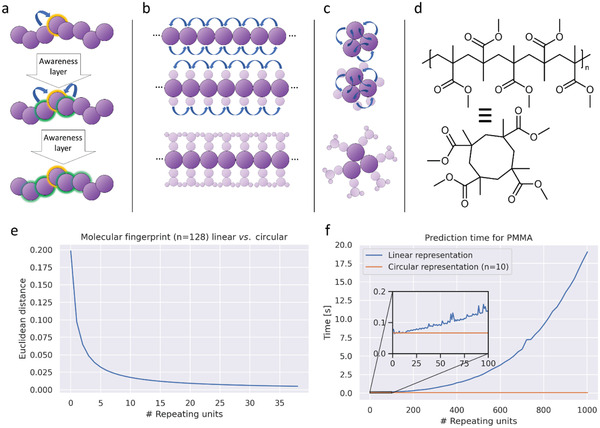
Awareness range and pseudo‐polymeric representation. a) Awareness range from the perspective of a single unit (highlighted in orange) and the units, whose features reach the unit during successive convolution (highlighted in green). b) Transfer of this interpretation to an infinite linear representation with each node at the same time. For each respective node, a small transparent copy of the information (other nodes) is attached to illustrate the “knowledge” of each node received during the convolution. As can be seen, all nodes have the same information since all neighboring moieties are equal. c) Transfer of this model to a reduced cyclic representation with three nodes: The convolution results are identical to the linear (infinite) representation. d) Transfer of the concept to a structural representation of linear polymers. For the model, the shown circular representation of four repeating units is the same as an infinity long polymer, as long as the atoms in the ten‐membered ring are not encoding a feature describing their membership in the ring (for a more detailed explanation, see the Supporting Information). e) Euclidian distance between the graph‐convolutional fingerprints of a circular representation of PMMA (ring size = 10) and a linear representation with an increasing number of repeating units. The Euclidian distance and, thus, the similarity of the outputs of the network approaches zero very fast. f) Explanatory computation times for predicting molecular fingerprints for a complete linear graph representation of the polymer compared to the reduced cyclic representation. The exponential increase of computation time with an increasing number of repeating units shows the high benefit of the circular representations.

As structural input for the GCN, the polymer can be utilized as a graph representation of the whole chemical structure of the polymer, meaning a graph with a number of nodes *N* equal to the number of atoms in the repeating unit *A*_R_ times the mean degree of polymerization *Dp*: |*N*| = |*A_R_* | *Dp. Such a graph would result in a large size leading to a significantly higher demand for computational power. Furthermore, most of the resulting feature‐vectors would be similar for the repeating units, except these at the *α*‐ and *ω*‐end of the chain or close to the terminal groups. However, since the terminal groups should have a very small effect on the particle size in the polymers utilized, the prediction can be performed with only the reoccurring identical feature vectors. This would represent an infinite long polymer chain, which is, as already described in other studies, a reasonably well descriptor for polymeric structures.^[^ ^33^ ^]^ To achieve a graph representation of a pseudo endless linear polymer, a cyclic representation of the polymer, with a ring size of 10 repeating units, was applied (**Figure** **2**). By doing so, the cyclic representation is equal to an infinite linear polymer from the viewpoint of each atom. Consequently, this simplification can be utilized since the model is not aware of bond‐angles, ring‐strain, macrocycles, and other effects that would occur during the transition from a linear polymer to a macrocycle.

Besides the structural input, the model also requires information about other parameters describing the utilized materials, such as the degree of polymerization of the polymer^[^ ^22^, ^34^ ^]^ and some preparation parameters (**Table** **1**). These parameters, together with the output of the GCN, are applied as input for the FCNN. Here, each layer consists of a fixed number of neurons, which are all connected to every neuron of the previous layer. The value of each neuron is the weighted sum of all previous neurons, which, together with a single bias value, is passed into the activation function of the neuron. A complete layer can be represented by:

(2) $$l_{n+1}=\;\sigma \left(l_{n}W_{n+1}+b_{n+1}\right)$$

where *l_n_* is the vector representing the values of the nodes of the *n* ^th^ layer in the network, $W_{n+1}\in M_{|l_{n}\left|\times \right|l_{n+1}|}$ is the weight matrix representing the connections between the two layers, *b_n_* is the bias vector of the *n* ^th^ layer, and *σ* the activation function of the layer.

**Table 1:** Overview of the theoretical possible parameter space utilized for the model design and points available in the training data

Parameter	Possible parameter space	Range applied in the current study
Repeating unit	Theoretically unlimited (to chemically reasonable monomers)	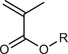 R = {methyl, ethyl, propyl, butyl, *i*‐butyl, *t*‐butyl, hexyl, phenyl, benzyl, isobornyl}
Degree of polymerization	[1, +∞)	[53, 1013]^a)^
Polymer concentration [g/L]	(0, *ρ*_*Polymer*_)	[1, 30]
Solvent	{all possible solvents}	{THF}
Antisolvent	{all possible solvents}	{Water}
Surfactant	{all possible surfactants}	{PVA}
Surfactant concentration [g/L]	[0, *ρ*_*surfactant*_)	[0, 0.0025]

[a,b], closed interval from a to b including a and b; (a,b), open interval from a to b excluding a and b; [a,b), half‐open interval from a to b including a and excluding b; {a,b,…,n}, set of elements, with all possibilities listed.

^a)^ Degree of polymerization was determined using the *M*_n_‐value obtained from SEC‐measurements.

The output of the FCNN is a single value representing the predicted size of the nanoparticle resulting from the precipitation under the input conditions. The whole model was implemented using the PyTorch framework^[^ ^35^ ^]^ and the PyTorch Geometric library^[^ ^36^ ^]^ for the GCN.

### 2.2 Particle Formulation and Model Training

To create a model capable of predicting nanoparticle sizes directly from the underlying polymeric structure, data for particle sizes from a broad spectrum of the parameter space have to be utilized for the training of the model. The number of possible parameter combinations is theoretically unlimited (Table 1), even for a single type of particle formulation with no additives besides a single surfactant. Due to this fact, we started by focusing on different commercially available, unpolar, uncharged methacrylates with carbon‐only side groups (in total, ten different monomers). For each monomer, multiple polymers were synthesized via controlled radical polymerization, that is, reversible addition‐fragmentation chain transfer (RAFT) polymerization,^[^ ^37^ ^]^ resulting in different samples of well‐defined molar masses (in total 33 polymers, **P1** to **P33**; for details see Supporting Information). For the nanoparticle formulation, high‐throughput nanoprecipitation via a liquid handling robot was applied.^[^ ^38^ ^]^ As solvent and anti‐solvent, we limited the formulation to tetrahydrofuran (THF, solvent) and water (anti‐solvent) and as a surfactant only poly(vinyl alcohol) (0.25%) or no additive was used. The polymer concentration in the solvent ranged from 1 to 30 g/L. Each formulation was performed three times and each one was measured three times to eliminate outlier and check for the experimental particle‐size derivation within the same formulation. In total, 3753 particle formulations were successfully investigated. After statistical processing of the data (for details see Supporting Information), 2813 measurements remained, which were utilized for training, validation, and testing of the model. The difference between the initial number of data and the remaining data after workup is mainly due to a strict removal of invalid data, for example, high dispersity values of particles or DLS‐measurement artifacts. A complete explanation and intermediate results of the processing are described in the Supporting Information. After a complete workup, one structure was removed entirely from the data and later used as test data to check the model capabilities to predict the particle size of non‐utilized polymer types. The remaining data were split into training and validation sets (8:2). During training, to prevent overfitting, a dropout ratio of 0.1 was defined for each convolutional and fully connected layer, which explains the higher inaccuracy of training prediction compared to validation metrics.

**Figure 3:**
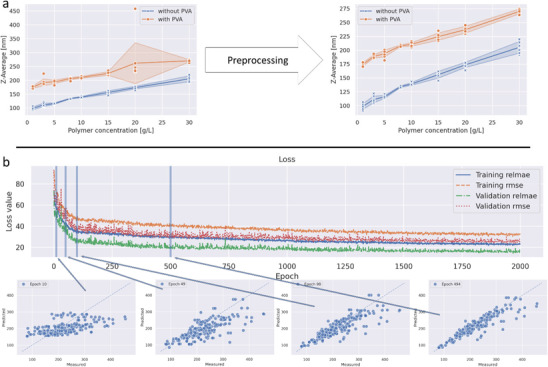
Data preprocessing and training. a) Raw data of polymer measurements were preprocessed to remove outliers and invalid samples, in which no particles are formed or measurement artifacts were recorded. b) Training curve of the model with exemplary validation plots at the epochs 10, 49, 98, and 494. Plotted are the mean absolute percentage error (MAPE) in percent and root‐mean‐square error (RMSE) as absolute values for the training and validation data.

During the training of the model, training and validation metrics were monitored continuously (**Figure** **3**). As a loss function, the mean absolute percentage error MAPE (Equation (3)) was chosen since the prediction should be capable of predicting the sizes with a relative loss as small as possible, meaning the prediction should be more accurate for smaller particle sizes.

(3) $$\frac{\sum_{i\;=\;0}^{n}\left|\frac{{\hat{y}}_{n}-y_{n}}{y_{n}}\right|}{n}$$

### 2.3 Prediction Results

**Figure 4:**
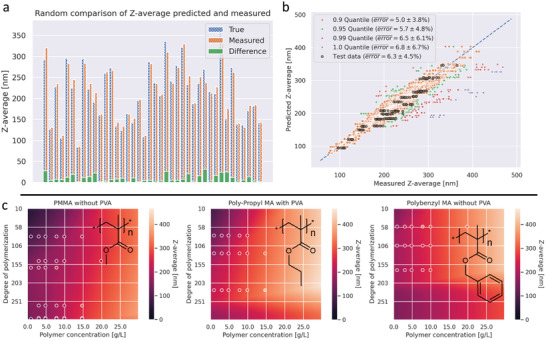
Overview of prediction results. a) Comparison of measured (blue) and predicted (orange) particle sizes for 40 random samples, including absolute error (green). b) Plot of measured values against predicted values for all nanoparticle sizes. For training data points, the 0.9, 0.95, 0.99, and 1.0 quantiles are highlighted with different colors, and the respective errors were calculated. Furthermore, the size values for the test data are plotted and fall into the same range as the training and validation data. c) Prediction space of poly(methyl methacrylate), poly(propyl methacrylate), and poly(benzyl methacrylate) over polymerization degrees from 10 to 300 and polymer concentrations of 0 to 30 g /L. Markers are inserted for data points from measurements from the original dataset.

The training was performed with multiple model instances, each time with a different testing polymer, which was removed prior to the training. After training the models, they converged to a mean absolute percentage error of about 5% (5.46% ± 0.65%) for the training data (**Table** **2**). As indicated in **Figure** **4**, most predictions fall close to the diagonal, where the prediction equals the real value. The model performance decreased slightly compared to the results where only one structure was removed from the training data set by removing polymers from the training set. For example, by removing simultaneously poly(*tert*‐butyl methacrylate) and poly(phenyl methacrylate), the prediction error for both increased slightly where as the model fits the training data better (MAPE = 5% for both removed and 5.8% and 5.3% for individual removed training sets). This finding can be mainly attributed to the decreasing, already limited size of the total available training data, which makes the model less generalizing.

**Table 2:** Prediction error for the model trained on different training set compositions

Testing polymer	MAPE train‐data [%]	MAPE test‐data^a)^ [%]	MAPE all^a)^ [%]	Worst performing group^b)^
Poly(methyl methacrylate)	4.6	17.6 (281)	6.7 (56.9)	Dp = 1013
Poly(ethyl methacrylate)	6.1	11.9 (13.3)	6.6 (6.8)	c = 1 g/L
Poly(propyl methacrylate)	5.4	13.8 (16.7)	6.1 (6.5)	c = 1 g/L
Poly(butyl methacrylate)	5.4	19 (23.8)	6.3 (7.4)	Dp = 180
Poly(*iso*‐butyl methacrylate)	6.8	6.3 (11.3)	6.7 (7.2)	Dp = 176
Poly(phenyl methacrylate)	5.3	12 (14.3)	5.6 (5.9)	Dp = 114
Poly(cyclohexyl methacrylate)	5.4	13.2 (33.5)	5.8 (7.9)	Dp = 178
Poly(*tert*‐butyl methacrylate)	5.8	16.5 (20.9)	6.4 (7.3)	without PVA
Poly(benzyl methacrylate)	4.9	13.5 (13.2)	5.4 (5.6)	without PVA
Poly(isobornyl methacrylate)	4.7	27.4 (40.2)	5.8 (7.9)	without PVA
Poly(phenyl methacrylate) + Poly(*tert*‐butyl methacrylate) ^c)^	5.0	14.4 (17.3)	6.4 (7.1)	Dp = 180
Mean value ± standard deviation	5.46 ± 0.65	15.12 ± 5.58	6.14 ± 0.48	

Each time all data points of a testing polymer were excluded from the training set prior to the default training/validation/test split. The model test was then performed with the test data from the split in combination with the complete test polymer dataset.

^a)^ The mean absolute percentage error (MAPE) for the test data and the complete dataset is given for the set without the sample group, which produces the highest error and for the bare dataset.

^b)^ 'Dp', the degree of polymerization that belongs to the group, which has the most significant influence on the error. 'c', polymer concentration in the stock solutions of the group with the most decisive influence on the error.

^c)^ Not included in mean value + standard deviation.

**Figure 5:**
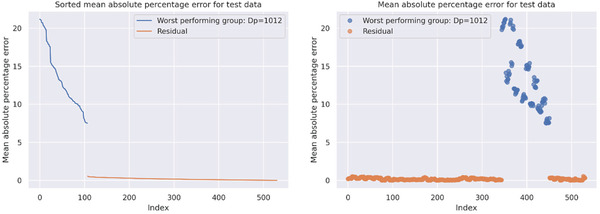
Mean absolute percentage error for the prediction of the particle sizes for the test data of the model, trained without poly(methyl methacrylate). The data are split into the group of the sample with the highest error, defined by the polymer, the degree of polymerization (Dp), polymer concentration, or additive (in the displayed example, a group with a specific Dp contributes the highest error). It can be clearly seen that this group can be separated from the remainder and thus qualify as unpredictable by the model.

For testing the model's capabilities to predict unknown structures, the initially removed formulation data were fed into the model, and the resulting predictions were compared to the measurement data. The results are listed in Table 2. Since some of the results seem to have a very high error, a closer investigation on the results shows that these errors usually result from one single group (polymers of a single polymer class with either specific degree of polymerization, stock solution concentration, or additive) which is hardly predictable. For example, the degree of polymerization for poly(methyl methacrylate) goes up to 1013 repeating units, whereas the highest degree of polymerization in the training data of the other polymers is 223. As a result, the model overestimates the influence of the higher molar masses resulting in a particle size prediction up to 20‐fold the measured value (**Figure** **5**). If these specific data points are removed from the testing data, the error drops down significantly. This indicates the current limitation of the model, that prediction can only be reliably performed within the parameter space of the training data, very nicely. As seen in Table 2, the group of the testing polymer which has the most decisive influence on the error is always a group with peripheral parameters (e.g., the highest degree of polymerization, the lowest polymer concentration, no surfactant), which also supports the statement that the model performs better, the closer the prediction input is to the center of the training parameter space.

The resulting mean deviations of the predictegd values (15% ± 5%) were, as expected, higher than the validation loss but remarkable for a particle size prediction of a previously unseen polymer.

As displayed in Figure 4 for arbitrary polymers, predictions can be performed for a range of degrees of polymerization and polymer concentrations resulting in a surface representing the particle size at every point. This can be very practical for scientists for targeting a specific particle size with a specific polymer since the required concentration and formulation conditions (e.g., application of surfactant) can be extracted directly without further calculations over a wide range of molar masses.

### 2.4 Limitations of the Model

The model, as represented here, can theoretically be used for every linear polymer under any particle formulation conditions. To limit the number of experiments required to train the model, we focused on methacrylates without hydrophobic side groups. Additionally, the formulation was performed with a single pair of solvent and antisolvent and only with PVA as the surfactant in a fixed concentration or without surfactant. As a result, the model, trained solely on our data, is only able to predict particles formulated under similar conditions. However, this is not an intrinsic limitation of the model, as it is trainable for custom formulations and different classes of polymers. For this purpose, an easy to use model configuration was implemented, which can be customized to match specific purposes. Furthermore, the model will be continuously trained to become better at generalizing over different classes of polymers and different formulation procedures.

## 3 Conclusion

With a combination of graph convolutional and fully connected neuronal networks, a model capable of predicting the nanoparticle size of different methacrylates prepared by nanoprecipitation was designed. The basis for this is a new experimentally generated dataset of 3753 nanoparticle formulations, which is publicly available.^[^ ^39^ ^]^ As input, the model takes nothing but the structure of the polymer as a node‐featurized graph, the degree of polymerization, and the nanoprecipitation parameters. Furthermore, a method was established to transform polymers into a circular reduced graph representation, which decreases the computational power by orders of magnitude compared to the classical graph representation for molecular data, which can be used as a general approach for machine learning models in polymer property predictions.

Our model was able to predict the particle size of an unknown polymer type with very good results, which demonstrates the high potential for property prediction in polymer science.

The model structure was designed in a very general way, which enables it to be reused, in theory, for any class of linear polymer as input in combination with an arbitrary number of additional numerical experimental inputs to predict different property values. Given enough high‐quality training data, our model can provide a head start in material preparation via in silico parameter optimization.

## Conflict of Interest

The authors declare no conflict of interest.

## Author Contribution

Software design, data processing, and machine‐learning were carried out by J.K.; polymer synthesis and characterization by T.S. and S.Z.; nanoprecipitation by A.V.; DLS‐measurements by A.V.; writing of the manuscript by J.K. and S.Z.; concept of the study by J.K, A.V., S.Z., and U.S.S.; supervision by S.Z. and U.S.S.; interpretation of measurement data by J.K, T.S., A.V., and S.Z.; correction of the manuscript by T.S., A.V., and U.S.S.

## Supporting information

Supporting Information

## Data Availability

The data that support the findings of this study are available in the Supporting Information of this article.
