# Supplementary material for: Prediction of Nanoparticle Sizes for Arbitrary Methacrylates Using Artificial Neuronal Networks
Source: Adv Sci (Weinh). 2021 Oct 23;8(23):2102429. doi: 10.1002/advs.202102429 (PMC8655218; doi:10.1002/advs.202102429)
Supplement: Supplementary file 1 — Supporting Information [file ADVS-8-2102429-s001.pdf]

WILEY-VCH

© 2021 Wiley-VCH GmbH

ADVANCED SCIENCE
Open Access

Supporting Information

for *Adv. Sci.*, DOI: 10.1002/advs.202102429

Prediction of nanoparticle sizes for arbitrary methacrylates using artificial neuronal networks

*Julian Kimmig, Timo Schuett, Antje Vollrath, Stefan Zechel, Ulrich S. Schubert**

© 2021 Wiley-VCH GmbH

Supporting Information

Prediction of nanoparticle sizes for arbitrary methacrylates using artificial neuronal networks

*Julian Kimmig, Timo Schuett, Antje Vollrath, Stefan Zechel, Ulrich S. Schubert**

J. Kimmig, T. Schuett, Dr. A. Vollrath, Dr. S. Zechel, Prof. U. S. Schubert

Laboratory of Organic and Macromolecular Chemistry (IOMC), Friedrich Schiller University Jena, Humboldtstr. 10, 07743 Jena, Germany, E-mail: ulrich.schubert@uni-jena.de

J. Kimmig, T. Schuett, Dr. A. Vollrath, Dr. S. Zechel, Prof. U. S. Schubert

Jena Center of Soft Matter (JCSM), Friedrich Schiller University Jena, Philosophenweg 7, 07743 Jena, Germany

Table of Contents

Materials and instrumentations 3
Polymer preparation 4
RAFT-Polymerisation of the methacrylates 4
NMR-analysis 4
Nanoparticle formulation 14
Dynamic light scattering measurements 15
Data preprocessing 15
An explanation for reduced cyclic representations 43
Conversion of structural data to a node-featurized graph representation 44
Featurization of the cyclic polymer representations 48
The model design 50
Hyperparameter optimization 51
Pretrained model overview 52
Pretrained model: np_model_0 54
Pretrained model: np_model_1 57
Pretrained model: np_model_2 59
Pretrained model: np_model_3 61
Pretrained model: np_model_4 64
Pretrained model: np_model_5 66
Pretrained model: np_model_6 68
Pretrained model: np_model_7 71
Pretrained model: np_model_8 73
Pretrained model: np_model_9 75
Pretrained model: np_model_10 78
Pretrained model: np_model_11 81
Pretrained model: np_model_12 83
Pretrained model: np_model_13 85
Pretrained model: np_model_14 88
References 91

Materials and instrumentations

All monomers as well as the initiator (2,2′-azo*bis*(2-methylpropionitrile, AIBN) and the RAFT-agent (2-cyano-2-propylbenzodithioat) were purchased from Sigma-Aldrich. Methanol was received from Thermo Fisher Scientific, and toluene and dimethylformamide from Acros Organics. Dimethylformamide (DMF) was dried over a molecular sieve under a nitrogen atmosphere. The liquid monomers that were used, were destabilized over a short AlOx column (neutral AlOx, obtained from Molecula). The dialysis tubings were purchased from Spectrum Labs (Spectra/PorTM, pre-wetted tubing, 3.5 kDa) and were rinsed with water before use. For the nanoparticle preparation, poly(vinyl alcohol) (PVA, Mowiol® 4-88) was purchased from Sigma-Aldrich and tetrahydrofuran (THF) was obtained from VWR and purified using a solvent purification system (SPS; Pure solv EN, InnovativeTechnology).

Nuclear magnetic resonance spectra were measured using a Bruker AC 300 (300 MHz) and Bruker AC 400 (400 MHz) spectrometer at 298 K if not stated differently. The chemical shift is given in parts per million (ppm on the δ scale) related to deuterated solvent.

Size exclusion chromatography measurements (SEC) were performed with the following setup: Shimadzu with CBM-20A (system controller), DGU-14A (degasser), LC-20AD (pump), SIL-20AHT (autosampler), CTO-10AC vp (oven), SPD-20A (UV detector), RID-10A (RI detector), PSS SDV guard/1000 Å/1,000,000 Å (5 µm particle size, supplier: PSS GmbH, separation range: 400 to 1,000,000 g/mol) chloroform/isopropanol/triethyl-amine [94/2/4] with 1 mL/min at 40 °C, poly(methyl methacrylate) (standards).

The data evaluation as well as the model training was performed on a custom build desktop computer using a 14 core Intel(R) Core(TM) i9-9940X CPU @ 3.30GHz and a Nvidia GeForce RTX 2080 Ti. The model training was performed utilizing the GPU using the Nvidia CUDA API.

Polymer preparation

For the preparation of the nanoparticles, different methacrylate-based homopolymers with variable side-groups and different degrees of polymerization were utilized. All polymers were synthesized *via* RAFT polymerization using a standard procedure:

RAFT-Polymerisation of the methacrylates

Solutions of azo*bis*isobutyronitrile (AIBN), 2-cyano-2-propylbenzodithioat (CTA) and the respective monomer in *N*,*N*-dimethylformamide (DMF) or toluene were prepared with different [M]:[CTA]:[I] ratios (**Table S1**) in a 10 mL microwave vial or a 100 mL round bottom flask. After closing the reaction vessel with a suitable septum, the reaction mixture was degassed by flushing with nitrogen for 20 minutes. The solution polymerizations were carried out in an oil-bath at 70 °C for 17 h. The polymers **P1-P12** were precipitated twice in cold methanol and dried *in vacuo* afterward. The polymers (**P13-P33**) were dried *in vacuo,* dissolved in 2 mL THF, purified *via* dialysis in THF (5 cycles of solvent exchange), and dried *in vacuo* again.

NMR-analysis

P1: ^{1}H NMR (300 MHz, $CDCl_3$) δ = 0.78 (s, 3H), 0.95 (s, 2H), 1.61-2.09 (m, 3H) ppm.

P2: ^{1}H NMR (300 MHz, $CDCl_3$) δ = 0.78 (s, 3H), 0.95 (s, 2H), 1.62-2.05 (m, 3H) ppm.

P3: ^{1}H NMR (300 MHz, $CDCl_3$) δ = 0.8 (s, 3H), 0.95 (s, 2H), 1.61-2.09 (m, 3H) ppm.

P4: ^{1}H NMR (300 MHz, $CDCl_3$) δ = 0.8 (s, 3H), 0.95 (s, 2H), 1.61-2.09 (m, 3H) ppm.

P5: ^{1}H NMR (300 MHz, $CDCl_3$) δ = 0.8 (s, 3H), 0.95 (s, 2H), 1.61-2.09 (m, 3H) ppm.

P6: ^{1}H NMR (300 MHz, $CDCl_3$) δ = 0.8 (s, 3H), 0.95 (s, 2H), 1.61-2.09 (m, 3H) ppm.

P7: ^{1}H NMR (400 MHz, $CDCl_3$) δ = 0.81-1.12 (m, 3H), 1.26 (s, 3H), 1.69-2.06 (m, 2H) 4.04 (s, 2H) ppm.

P8: ^{1}H NMR (400 MHz, $CDCl_3$) δ = 0.79-1.14 (m, 3H), 1.26 (s, 3H), 1.73-2.07 (m, 2H) 4.03 (s, 2H) ppm.

P9: ^{1}H NMR (400 MHz, $CDCl_3$) δ = 0.76-1.11 (m, 3H), 1.26 (s, 3H), 1.74-2.08 (m, 2H) 4.04 (s, 2H) ppm.

P10: ^{1}H NMR (400 MHz, $CDCl_3$) δ = 0.76-1.19 (m, 6H), 1.67 (s, 2H), 1.75-2.13 (m, 2H), 3.92 (m, 2H) ppm.

P11: ^{1}H NMR (400 MHz, $CDCl_3$) δ = 0.79-1.17 (m, 6H), 1.67 (s, 2H), 1.67-2.09 (m, 2H), 3.92 (m, 2H) ppm.

P12: ^{1}H NMR (300 MHz, $CDCl_3$) δ = 0.61-1.10 (m, 6H), 1.58 (s, 2H), 1.67-2.14 (m, 2H), 3.83 (s, 2H) ppm.

P13: ^{1}H NMR (400 MHz, $CDCl_3$) δ = 0.77-1.15 (m, 6H), 1.34-1.52 (m, 2H), 1.62 (s, 2H), 1.75-2.10 (m, 2H), 3.95 (s, 2H) ppm.

P14: ^{1}H NMR (400 MHz, $CDCl_3$) δ = 0.78-1.14 (m, 6H), 1.35-1.51 (m, 2H), 1.63 (s, 2H), 1.74-2.10 (m, 2H), 3.96 (s, 2H) ppm.

P15: ^{1}H NMR (300 MHz, $CDCl_3$) δ = 0.68-1.05 (m, 6H), 1.24-1.42 (m, 2H), 1.46-1.63 (m, 2H), 1.67-1.99 (m, 2H), 3.88 (s, 2H) ppm.

P16: ^{1}H NMR (400 MHz, $CDCl_3$) δ = 0.79-1.15 (m, 9H), 1.84 (s, 1H), 1.93 (s, 2H), 3.71 (s, 2H) ppm.

P17: ^{1}H NMR (400 MHz, $CDCl_3$) δ = 0.79-1.16 (m, 9H), 1.84 (s, 1H), 1.93 (s, 2H), 3.71 (s, 2H) ppm.

P18: ^{1}H NMR (400 MHz, $CDCl_3$) δ = 0.78-1.15 (m, 9H), 1.84 (s, 1H), 1.93 (s, 2H), 3.71 (s, 2H) ppm.

P19: ^{1}H NMR (300 MHz, $CDCl_3$) δ = 0.83-1.14 (m, 3H), 1.27-1.47 (s, 9H), 1.68-1.86 (m, 3H) ppm.

P20: ^{1}H NMR (400 MHz, $CDCl_3$) δ = 0.55-1.21 (m, 3H), 1.36-1.55 (m, 9H), 1.83 (s, 3H) ppm.

P21: ^{1}H NMR (300 MHz, $CDCl_3$) δ = 0.85-1.12 (m, 3H), 1.26-1.46 (m, 9H), 1.67-1.86 (m, 3H) ppm.

P22: ^{1}H NMR (300 MHz, $CDCl_3$) δ = 1.06-1.41 (m, 7H), 1.41-1.59 (m, 2H), 1.66 (s, 2H), 1.71-1.90 (m, 4H), 4.64 (s, 1H) ppm.

P23: ^{1}H NMR (400 MHz, $CDCl_3$) δ = 1.14-1.47 (m, 7H), 1.56 (s, 2H), 1.73 (s, 2H), 1.83 (s, 4H), 4.71 (s, 1H) ppm.

P24: ^{1}H NMR (400 MHz, $CDCl_3$) δ = 1.15-1.47 (m, 7H), 1.56 (s, 2H), 1.73 (s, 2H), 1.78-1.96 (m, 4H), 4.71 (s, 1H) ppm.

P25: ^{1}H NMR (300 MHz, $CDCl_3$) δ = 0.52-0.97 (m, 3H), 1.59-2.06 (m, 2H), 4.69-5.01 (m, 2H), 7.10-7.30 (m, 5H) ppm.

P26: ^{1}H NMR (300 MHz, $CDCl_3$) δ = 0.49-0.97 (m, 3H), 1.60-2.07 (m, 2H), 4.81 (s, 2H), 7.20 (s, 5H) ppm.

P27: ^{1}H NMR (300 MHz, $CDCl_3$) δ = 0.51-0.97 (m, 3H), 1.60-2.06 (m, 2H), 4.81 (s, 2H), 7.20 (s, 5H) ppm.

P28: ^{1}H NMR (300 MHz, $CDCl_3$) δ = 1.27-1.63 (m, 3H), 2.05-2.50 (m, 2H), 6.88-7.33 (m, 5H) ppm.

P29: ^{1}H NMR (300 MHz, $CDCl_3$) δ = 1.27-1.62 (m, 3H), 2.07-2.52 (m, 2H), 6.87-7.34 (m, 5H) ppm.

P30: ^{1}H NMR (300 MHz, $CDCl_3$) δ = 1.27-1.63 (m, 3H), 2.06-2.52 (m, 2H), 6.89-7.33 (m, 5H) ppm.

P31: ^{1}H NMR (300 MHz, $CDCl_3$) δ = 0.65-1.18 (m, 14H), 1.40-1.95 (m, 7H), 4.26 (s, 1H) ppm.

P32: ^{1}H NMR (300 MHz, $CDCl_3$) δ = 0.64-1.19 (m, 14H), 1.40-1.94 (m, 7H), 4.26 (s, 1H) ppm.

P33: ^{1}H NMR (300 MHz, $CDCl_3$) δ = 0.65-1.19 (m, 14H), 1.40-1.92 (m, 7H), 4.26 (s, 1H) ppm.

Table S1: Reaction details for the RAFT-polymerization.

Polymer	Monomer	V_{DMF} [mL]	V_{Toluol} [mL]	m_{CTA} [mg]	$m_{Initiator}$ [mg]
**P1**	Poly(methyl methacrylate)	4.99	-	29.48	5.47
**P2**		4.99	-	14.74	2.73
**P3**		4.99	-	7.39	1.36
**P4**		40	-	88.29	16.30
**P5**		40	-	32.26	6.19
**P6**		40	-	221.0	41.0
**P7**	Poly(ethyl methacrylate)	4.38	-	25.86	4.80
**P8**		4.38	-	12.93	2.40
**P9**		4.38	-	6.46	1.20
**P10**	Poly(propyl methacrylate)	3.90	-	23.03	4.27
**P11**		3.90	-	11.51	2.14
**P12**		3.90	-	5.76	1.07
**P13**	Poly(butyl methacrylate)	3.52	-	20.75	3.85
**P14**		3.52	-	10.38	1.92
**P15**		3.52	-	5.19	0.96
**P16**	Poly(*i*-butyl methacrylate)	3.52	-	20.75	3.85
**P17**		3.52	-	10.38	1.92
**P18**		3.52	-	5.19	0.96
**P19**	Poly(*t*-butyl methacrylate)	3.52	-	20.75	3.85
**P20**		3.52	-	10.38	1.92
**P21**		3.52	-	5.19	0.96
**P22**	Poly(cyclohexyl methacrylate)	2.97	-	17.55	3.25
**P23**		2.97	-	8.77	1.63
**P24**		2.97	-	4.39	0.81
**P25**	Poly(benzyl methacrylate)	2.84	-	16.75	3.11
**P26**		2.84	-	8.37	1.55
**P27**		2.84	-	4.19	0.78
**P28**	Poly(phenyl methacrylate)	3.08	-	18.20	3.37
**P29**		3.08	-	9.10	1.69
**P30**		3.08	-	4.55	0.84
**P31**	Poly(isobornyl methacrylate)	-	2.25	13.27	2.46
**P32**		-	2.25	6.64	1.23
**P33**		-	2.25	3.32	0.62

Table S2: Characterization results of synthesized polymers. All SEC measurements were performed using size exclusion chromatography using chloroform/isopropanol/triethylamine (94/2/4 v%) against a PMMA standard.

Polymer	M_n	M_w	Đ
**P1**	29.700	34.400	1.16
**P2**	16.500	18.900	1.15
**P3**	101.400	191.000	1.88
**P4**	14.700	15.900	1.08
**P5**	26.400	31.200	1.18
**P6**	8.200	9.000	1.10
**P7**	10.000	11.400	1.14
**P8**	15.200	17.800	1.18
**P9**	22.400	26.800	1.19
**P10**	9.700	11.100	1.15
**P11**	16.400	18.000	1.10
**P12**	28.600	32.900	1.15
**P13**	8.400	10.100	1.20
**P14**	15.700	17.900	1.14
**P15**	25.500	32.200	1.26
**P16**	10.800	12.400	1.15
**P17**	13.900	15.300	1.10
**P18**	25.000	28.700	1.15
**P19**	9.000	10.300	1.14
**P20**	13.600	16.800	1.23
**P21**	25.900	29.400	1.13
**P22**	11.700	13.000	1.11
**P23**	20.600	22.300	1.09
**P24**	14.200	21.300	1.50
**P25**	10.200	12.200	1.18
**P26**	19.000	22.800	1.20
**P27**	30.000	37.700	1.25
**P28**	10.800	14.300	1.32
**P29**	18.600	24.600	1.32
**P30**	27.900	38.300	1.37
**P31**	12.300	13.500	1.10
**P32**	18.600	21.600	1.16
**P33**	26.800	30.700	1.15

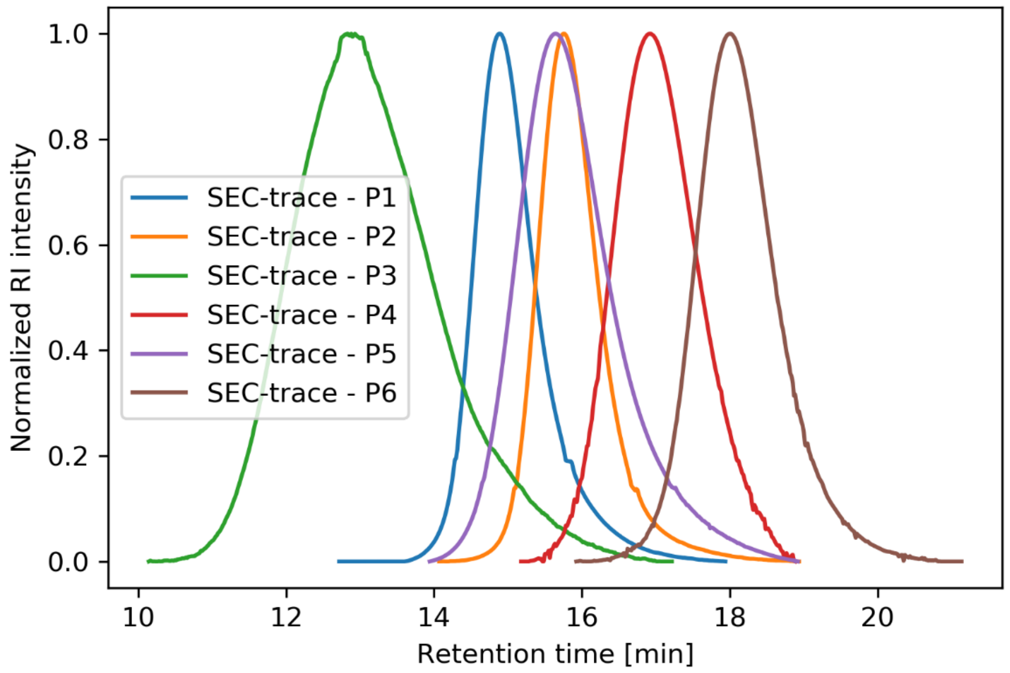

Figure S1: SEC-traces of Polymers **P1-P6**.

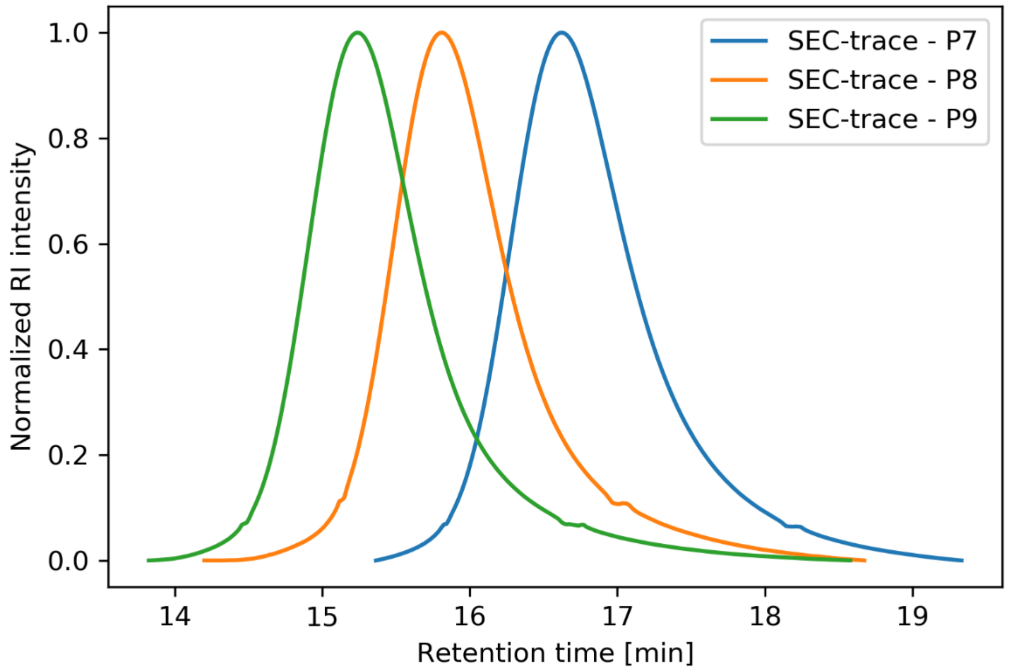

Figure S2: SEC-traces of Polymers **P7-P9**.

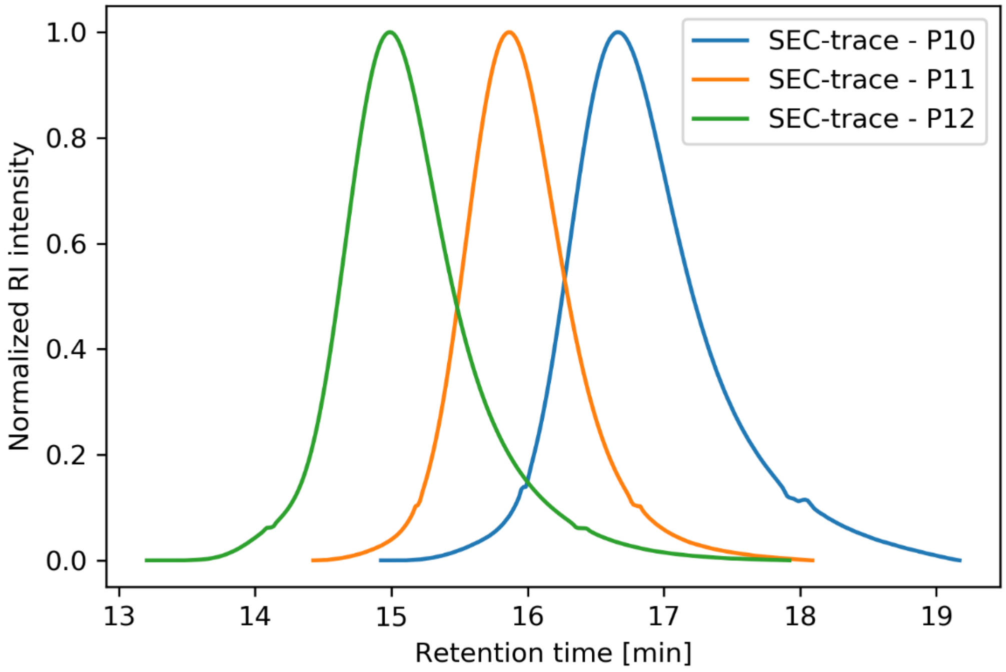

Figure S3: SEC-traces of Polymers **P10-P12**.

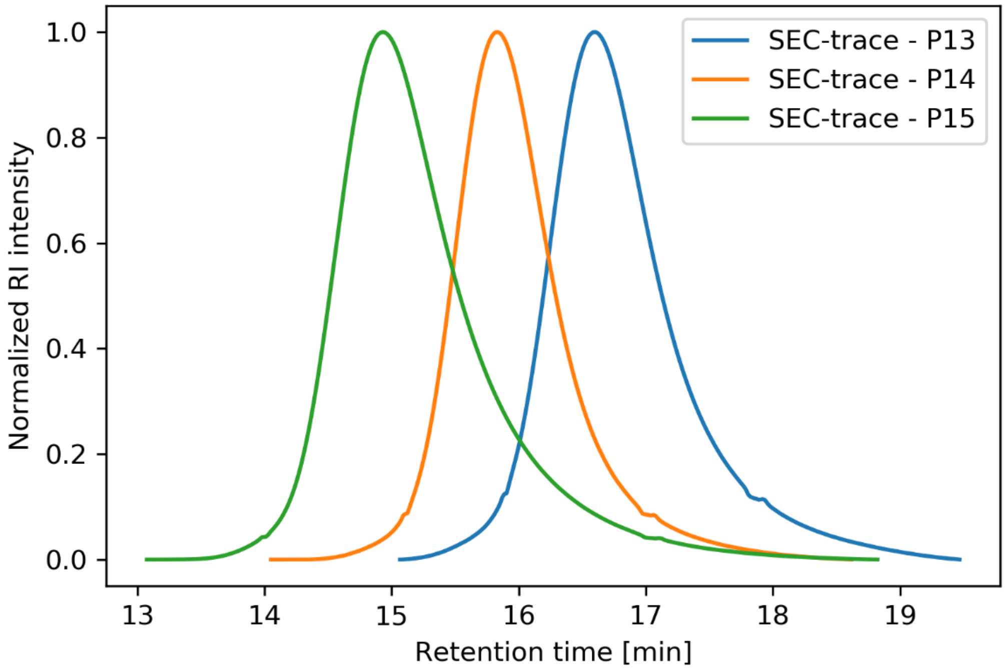

Figure S4: SEC-traces of Polymers **P13-P15**.

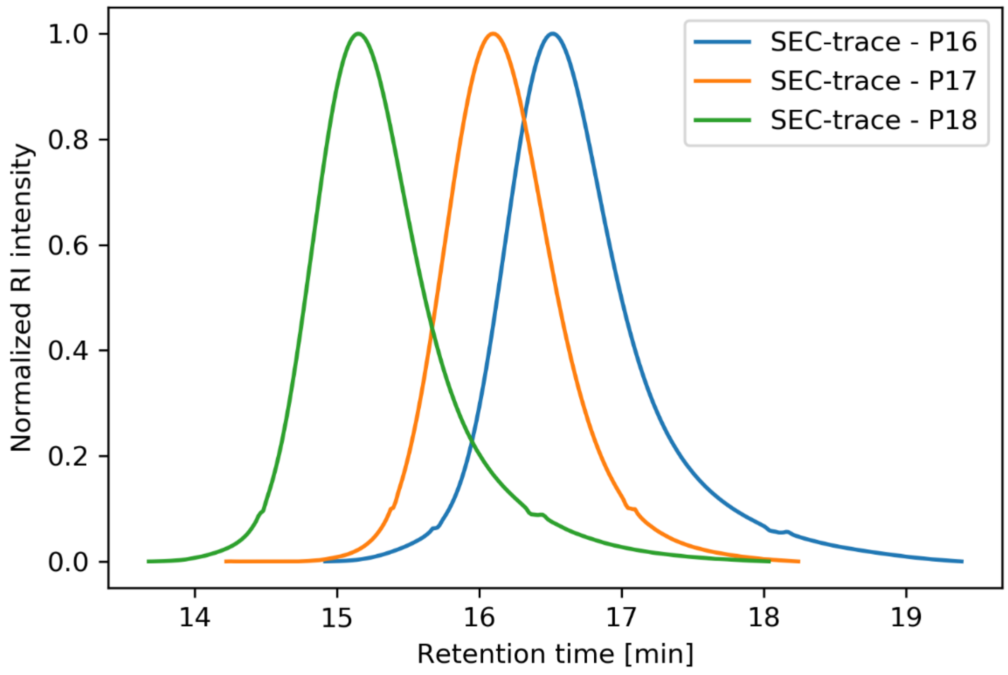

Figure S5: SEC-traces of Polymers **P16-P18**.

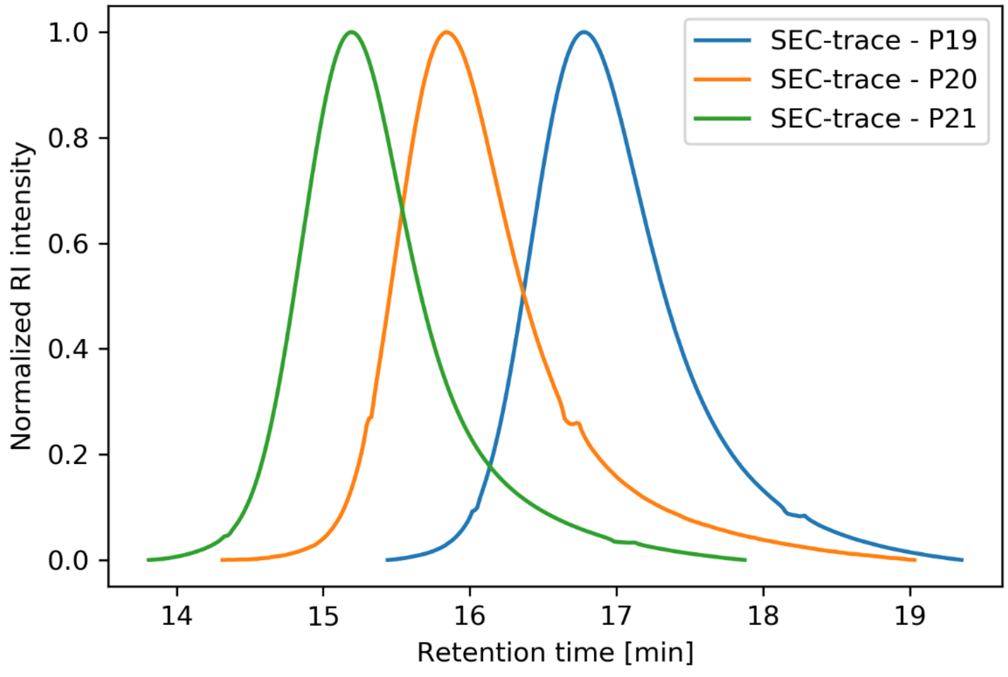

Figure S6: SEC-traces of Polymers **P19-P21**.

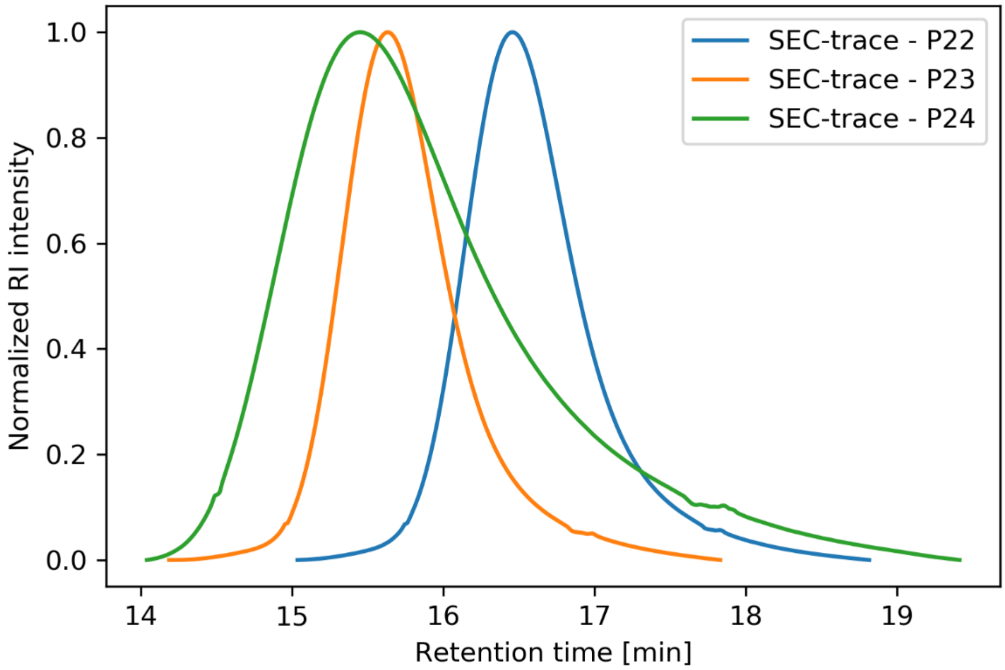

Figure S7: SEC-traces of Polymers **P22-P24**.

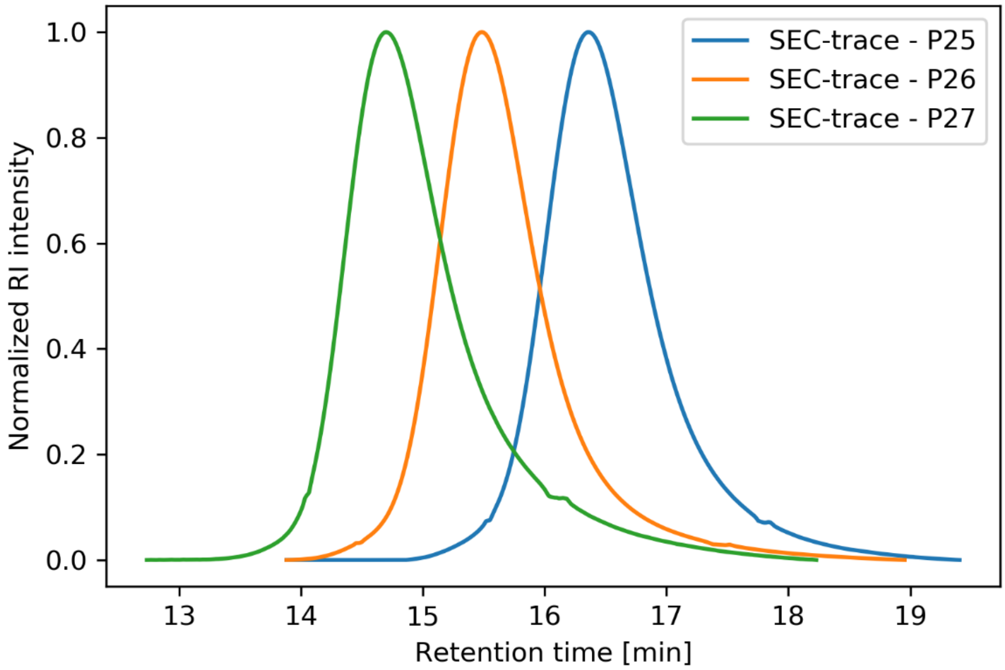

Figure S8: SEC-traces of Polymers **P25-P27**.

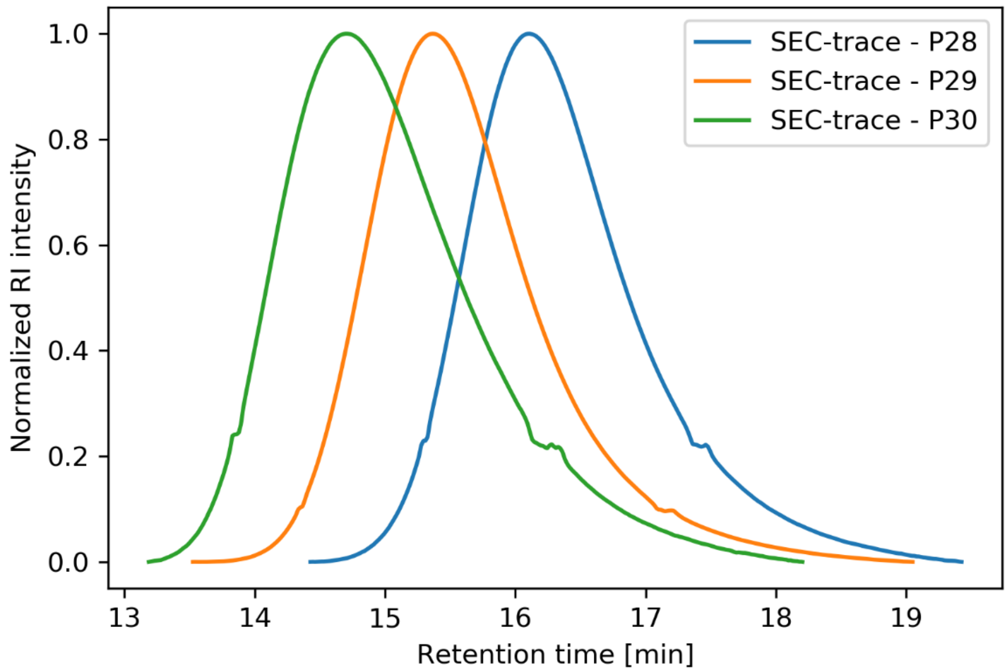

Figure S9: SEC-traces of Polymers **P28-P30**.

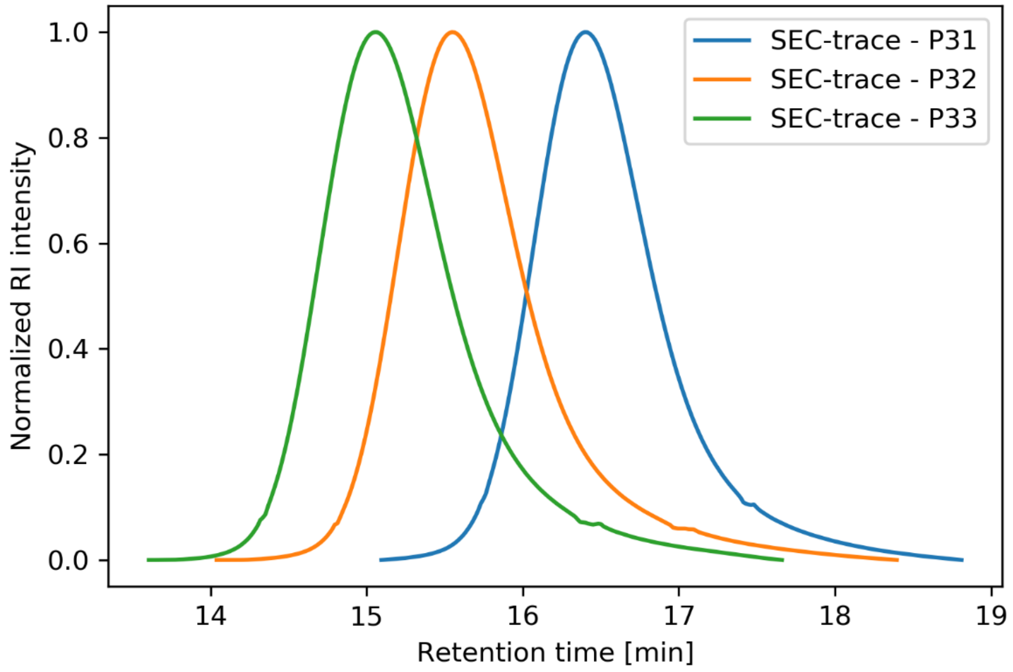

Figure S10: SEC-traces of Polymers **P31-P33**.

Nanoparticle formulation

The formulation of the nanoparticles *via* high-throughput nanoprecipitation was performed using a FasTrans liquid handling robot (Analytik Jena AG, Jena, Germany). The formulation was performed in a 96 well format using clear, flat bottom 96 well plates made of polypropylene from Greiner Bio-One GmbH, Frickenhausen, Germany. For each polymer, an initial polymer stock solution with a concentration of 30 mg mL^{-1} was prepared in THF, from which a dilution series with concentrations of c = 1, 3, 5, 8, 10, 15, 20 and 30 mg mL^{-1} was prepared with the liquid handling robot. Afterwards, nanoprecipitation was carried out by pipetting 5 to 50 µL of the respective polymer solution into 200 µL purified water (Thermo Scientific, GenPure ultrapure water system) or water containing 0.25% (w/v) PVA (see **Table S3**). The pipetted volume of the polymer solution was varied in order to keep the final nanoparticle concentration below 1 mg mL^{-1}. With this constant nanoparticle concentration, concentration dependent destabilizing effects and enhanced aggregation of the nanoparticles was avoided. Furthermore, misleading DLS measurements due to high particle concentrations, which often results in multiple scattering patterns and shifted size reports, were further prevented.

Table S3: Details for the nanoprecipitation of polymers into purified water or water containing 0.25% (w/v) PVA.

$c_{polymer\ in\ THF}$ [mg mL^{-1}]	$V_{water/\ PVA}$ [µL]	$V_{Polymer\ solution}$ [µL]	$M_{Polymer}$ [µg]	Ratio (water:solvent)	$c_{Nanoparticles}$ [µg $µL^{-1}$]
1	200	50	50	4.0	0.25
3	200	50	150	4.0	0.75
5	200	30	150	6.7	0.75
8	200	18.7	149.6	10.7	0.748
10	200	15	150	13.3	0.75
15	200	10	150	20.0	0.75
20	200	7.5	150	26.7	0.75
30	200	5	150	40.0	0.75

After the nanoprecipitation process (dropping of organic polymer solution into the aqueous phase), the suspension was mixed by double aspiration and release. Each formulation was performed three times. For the evaporation of the solvent, the 96 well plate was left under an

exhaust system for at least for 2 h. Afterwards, the formulations were inspected by eye and checked for the formation of aggregates visible by white flakes or threads or complete thick turbidity. The well with visible aggregation were marked as “aggregated / not valid” and not further characterized *via* DLS.

Dynamic light scattering measurements

The Zetasizer Nano ZS (Malvern Instruments, Malvern, Worcestershire, United Kingdom) was used for dynamic light scattering (DLS) measurements. It utilizes a laser wavelength of λ = 633 nm and measures the counts backscattered at an angle of 173°. Prior DLS analysis, 100 µL of the nanoparticle suspensions were further diluted with 100 µL water and transferred into a microcuvette (Brand, Wertheim, Germany). Three repeated measurements at 25 °C with 10 seconds measurement time were performed. The Z-Average value (harmonic averaged *particle diameter*, intensity weighted) and the polydispersity index (PDI value, width of the size distribution) were obtained utilizing the cumulant analysis method. At the end, nine data points were collected out of the three measurements for the three individual formulations per polymer and concentration.

Data preprocessing

Since not all formulations (n = 3753) worked in a suitable manner, non-useable data and outliers had to be removed before training of the model. For example, some measurement results showed particle sizes up to a few hundreds of µm, probably due to precipitation of the polymer material or impurities in the well. For the preprocessing, all measurements with particle sizes over a fixed threshold (>500 nm)[1] were removed from the sample set (n = 3454).

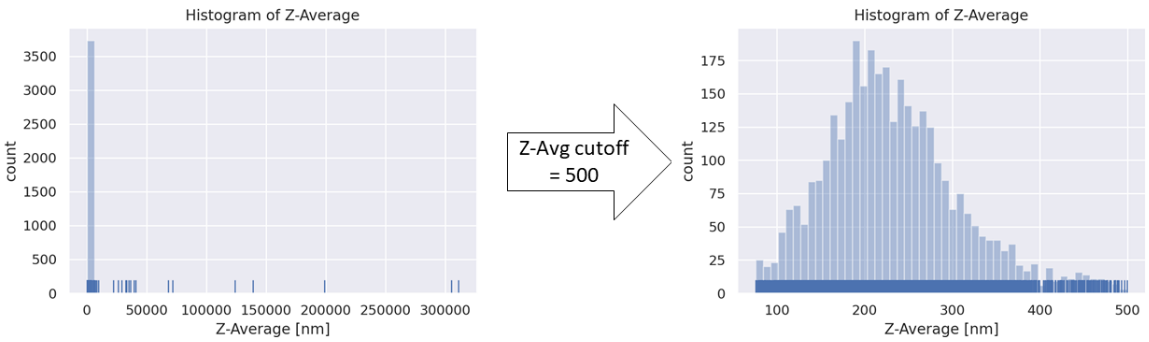

Figure S11: Change of particle size distribution after removing measurements with large particle sizes.

Afterwards, all samples with a dispersity larger than a fixed threshold (>0.3)[2] were removed from the remaining set resulting in a data set of nanoparticles that should meet the criteria of a quality formulation (n = 3093).

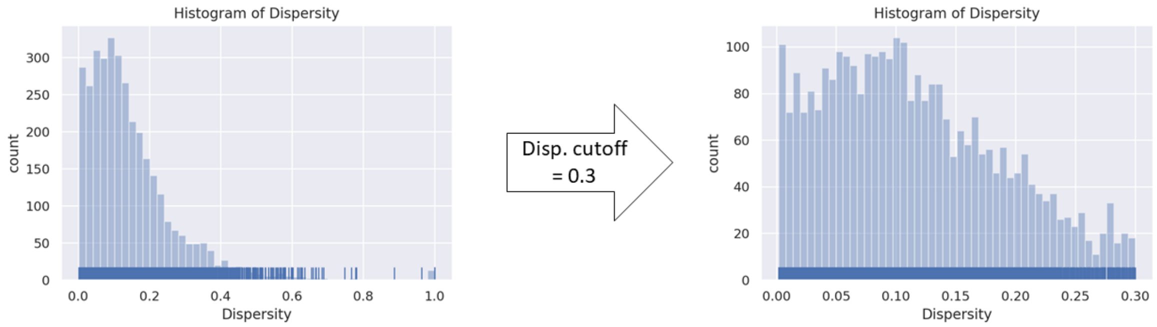

Figure S12: Change of dispersity distribution.

Since each formulation was performed nine times, outliers can be detected *via* Grubb's test, resulting in an outlier free dataset (n = 3052).

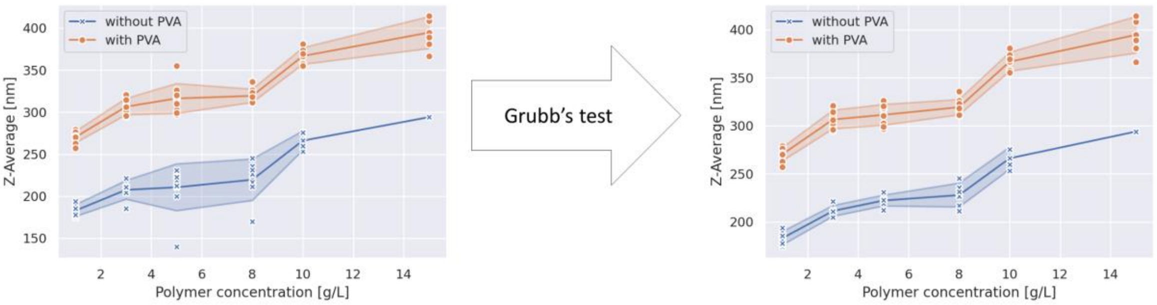

Figure S13: Exemplary change of the dataset (**P26**) *via* Grubb's test for outliers. In this example, for the polymer with PVA singe samples were removed at a concentration of 3 to 8 g/L.

For some formulations, the size variation within a formulation group varies by a few hundred nanometers. This difference in sizes may indicate that under the corresponding formulation conditions, a reproducible formulation is not possible, and, thus, these samples were removed as well (n = 3035).

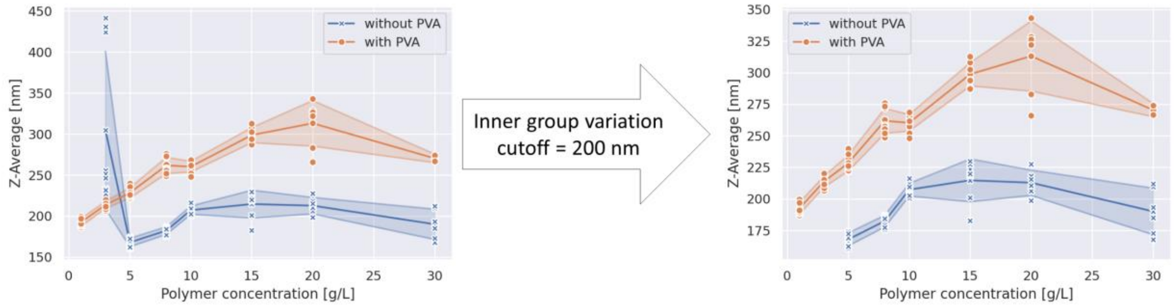

Figure S14: Exemplary change of the dataset (**P22**) when the inner group variation is higher than 200 nm. In this example, for the polymer without PVA at a concentration of 3 g/L.

Some sampling groups contain only one or two samples, which might be prone to not-detectable outliers or are remainder of groups with poor-quality formulation results. All formulation groups with a sample number smaller than three samples were removed (n = 3010) to prevent the model from training on such data.

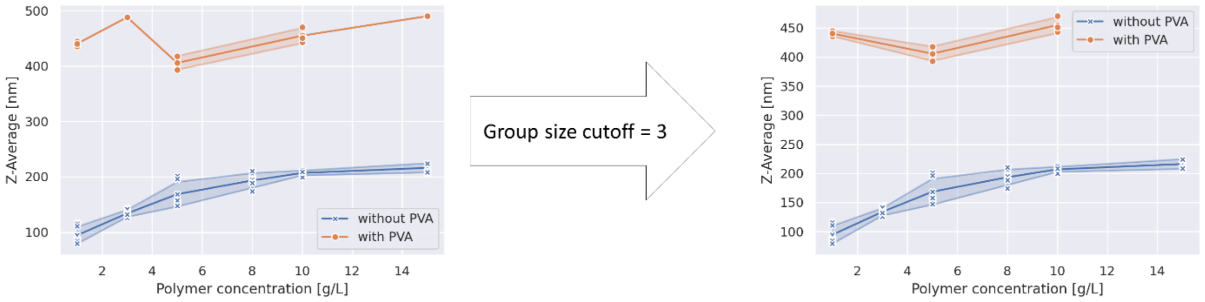

Figure S15: Exemplary change of the dataset (**P8**) when the group size is smaller than 3. In this example for the polymer with PVA, the sample groups at 3 g/L and 15 g/L were removed.

As a standard rule, particle sizes increase in diameter with increasing polymer concentrations.[3] Suppose the particle size at low concentrations is larger than the next formulations with increased concentration; it can be assumed that these formulations did not work, and no particles formed, but only artifacts were measured. Analogs, if particle sizes drop at higher concentrations, this can indicate precipitation of polymer, and only small residual particles of the polymer were measured. Samples that show one of these characteristics were excluded from the set (n = 2823).

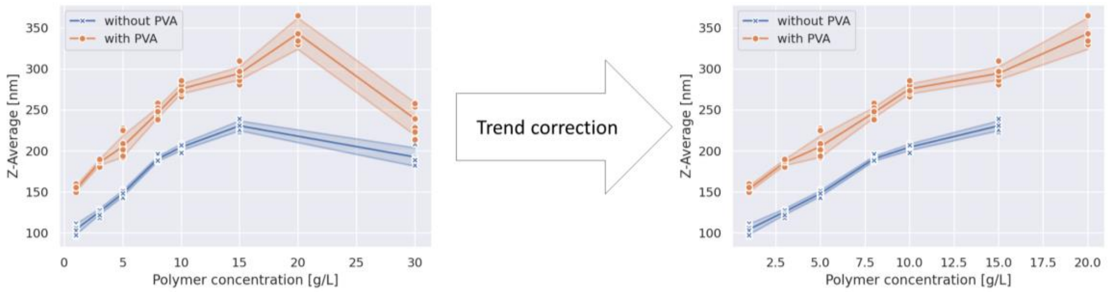

Figure S16: Exemplary change of the dataset (**P16**), after trend correction. In this example, for the formulations with as well as for the formulations without PVA, the samples with a polymer concentration greater than 20 g/L were removed.

Finally, all polymers with less than two valid concentrations were removed from the set since this indicates that these polymers are not suitable for nanoparticle formulation under the given conditions, and they were removed entirely from the training data (n = 2813).

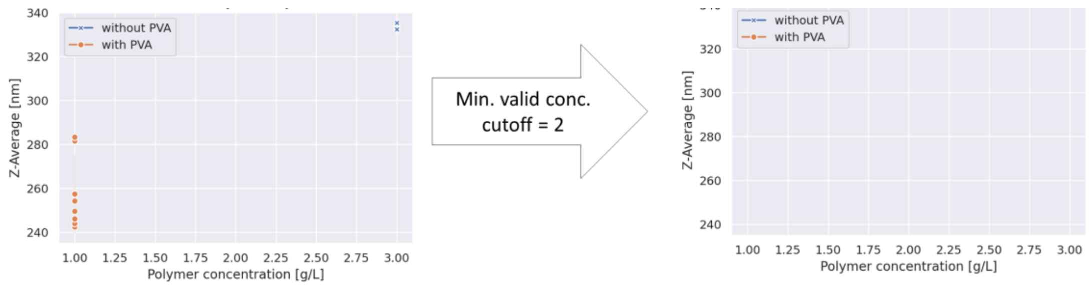

Figure S17: Exemplary change of the dataset (**P33**), after removal of formulation groups where the number of valid concentrations is smaller than two. In this example, all formulations for the specific polymer had to be removed.

Table S4: Overview of the complete remaining dataset after preprocessing.

**Polymer**	
**P1**	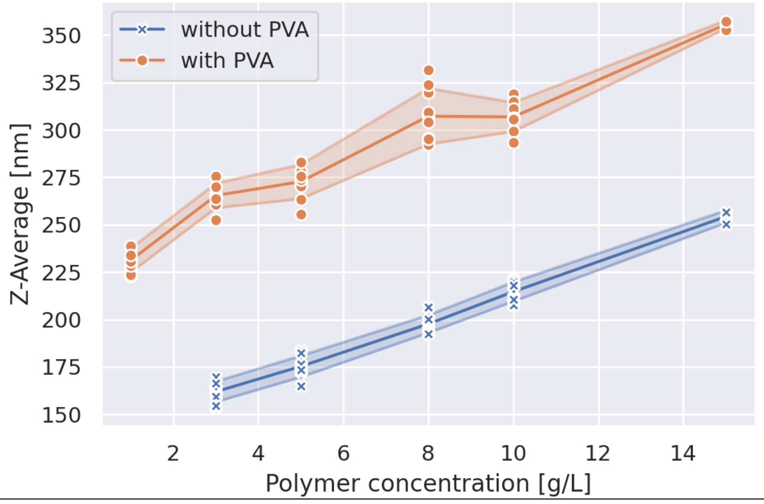
**P2**	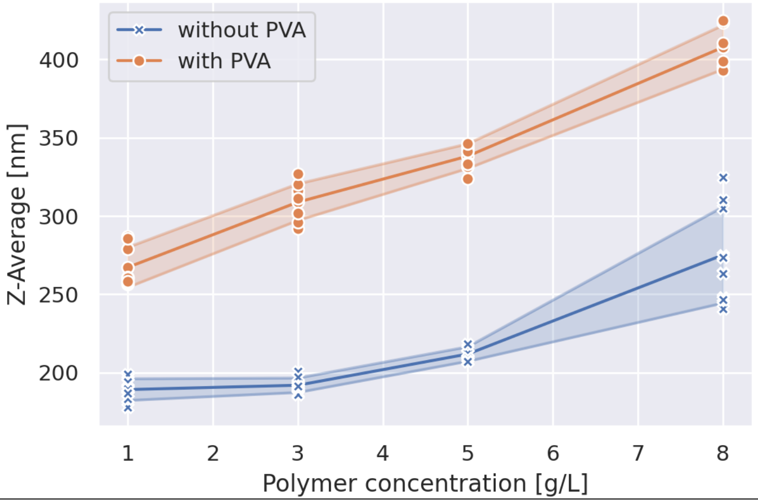
**P3**	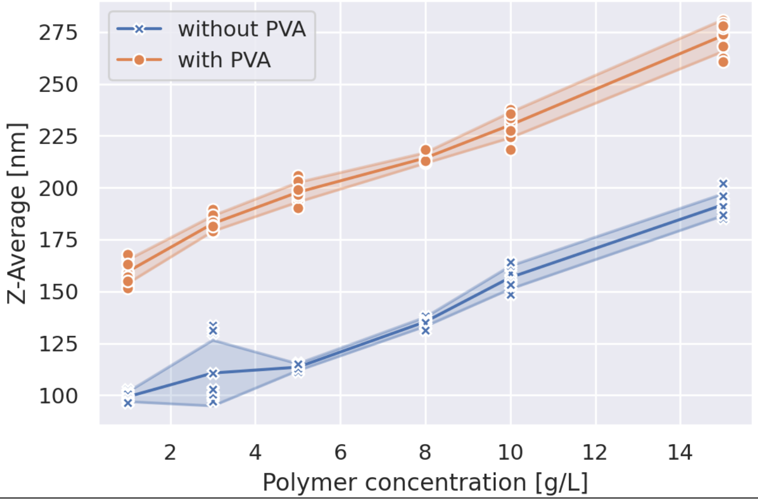
	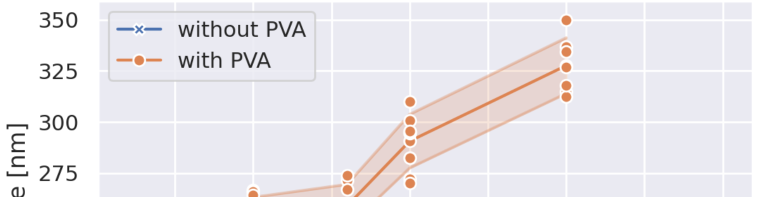

Statistical evaluation of the complete dataset

Table 1:Statistical parameters of the complete training data grouped by formulation parameters.

c	Additive	Conc. [g/L]	Sample count	Mean value [nm]	Standard deviation [nm]	Minimum value [nm]	25% Quantile [nm]	50% Quantile [nm]	75% Quantile [nm]	Max [nm]	Variance [nm]	Coefficient of variation	Bias corrected coefficient of variation	Standard error [nm]	Rel. standard error
P01	None	3	9	162.0	5.4	154.0	159.4	163.0	166.1	169.9	28.8	3.3%	3.4%	1.8	1.1%
		5	9	175.3	5.6	165.0	173.3	174.5	176.4	184.0	31.5	3.2%	3.3%	1.9	1.1%
		8	9	197.7	4.7	192.6	193.1	199.2	199.9	206.6	21.7	2.4%	2.4%	1.6	0.8%
		10	9	214.7	5.1	207.8	210.5	215.0	218.9	221.2	25.8	2.4%	2.4%	1.7	0.8%
		15	3	254.3	3.3	250.5	253.2	255.8	256.2	256.5	10.8	1.3%	1.4%	1.9	0.7%
	PVA	1	9	231.3	6.3	222.9	226.7	230.6	238.0	238.7	39.9	2.7%	2.8%	2.1	0.9%
		3	9	265.4	6.5	252.5	263.5	265.3	270.0	275.4	41.9	2.4%	2.5%	2.2	0.8%
		5	9	272.7	9.0	255.4	270.3	273.7	278.0	283.2	81.0	3.3%	3.4%	3.0	1.1%
		8	9	307.2	14.8	292.3	295.3	304.1	319.9	331.5	217.8	4.8%	4.9%	4.9	1.6%
		10	9	306.8	7.7	293.5	305.5	305.9	311.3	318.9	58.9	2.5%	2.6%	2.6	0.8%
		15	4	355.7	2.1	352.7	354.8	356.3	357.2	357.3	4.5	0.6%	0.6%	1.1	0.3%
P02	None	1	9	189.1	6.9	178.0	183.4	189.5	194.0	199.0	47.8	3.7%	3.8%	2.3	1.2%
		3	9	191.8	4.6	186.0	190.1	191.3	192.5	200.5	21.2	2.4%	2.5%	1.5	0.8%
		5	6	211.8	4.6	206.9	208.1	210.9	215.3	218.1	21.4	2.2%	2.3%	1.9	0.9%
		8	9	275.0	30.7	240.9	247.8	263.8	304.8	324.5	944.0	11.2%	11.5%	10.2	3.7%
	PVA	1	9	267.2	12.8	256.4	258.1	260.3	278.8	287.1	163.2	4.8%	4.9%	4.3	1.6%
		3	9	308.8	11.7	291.9	301.6	311.4	316.4	326.8	136.6	3.8%	3.9%	3.9	1.3%
		5	9	338.2	7.9	324.0	333.1	341.4	344.6	346.2	61.9	2.3%	2.4%	2.6	0.8%
		8	6	407.7	14.1	393.0	396.5	404.6	420.4	424.7	198.3	3.5%	3.6%	5.7	1.4%
P03	None	1	9	99.2	2.3	96.4	97.1	98.5	101.0	102.9	5.4	2.4%	2.4%	0.8	0.8%
		3	9	110.7	15.8	97.1	99.7	102.2	129.9	133.6	250.9	14.3%	14.7%	5.3	4.8%
		5	9	113.5	1.6	111.3	111.9	113.7	114.9	115.7	2.7	1.5%	1.5%	0.5	0.5%

		**8**	9	135.4	2.2	131.4	133.7	136.0	137.2	138.2	4.8	1.6%	1.7%	0.7	0.5%
		**10**	9	156.7	5.5	148.6	153.0	154.5	162.3	164.0	30.5	3.5%	3.6%	1.8	1.2%
		**15**	9	191.7	5.3	185.1	187.2	191.1	193.5	202.2	27.6	2.7%	2.8%	1.8	0.9%
	**PVA**	**1**	9	159.6	5.7	151.6	155.0	158.8	163.2	167.8	32.2	3.6%	3.7%	1.9	1.2%
		**3**	9	182.7	3.7	178.8	179.2	181.6	184.8	189.5	13.8	2.0%	2.1%	1.2	0.7%
		**5**	9	197.8	4.7	190.2	195.5	197.4	198.9	205.9	22.4	2.4%	2.5%	1.6	0.8%
		**8**	8	214.3	2.5	211.9	212.6	213.5	215.2	218.3	6.4	1.2%	1.2%	0.9	0.4%
		**10**	9	230.2	6.1	218.5	227.4	231.4	234.5	237.8	37.6	2.7%	2.7%	2.0	0.9%
		**15**	9	273.2	7.7	260.6	268.0	277.1	278.8	280.7	58.5	2.8%	2.9%	2.6	0.9%
**P04**	**None**	**3**	5	163.1	2.3	161.1	161.8	162.0	163.5	166.9	5.4	1.4%	1.5%	1.0	0.6%
		**5**	5	166.3	1.3	164.5	165.6	166.2	167.4	167.7	1.7	0.8%	0.8%	0.6	0.4%
		**8**	8	167.3	5.1	162.5	162.7	165.8	171.8	175.0	26.1	3.1%	3.1%	1.8	1.1%
		**10**	5	180.0	13.6	162.1	177.8	179.5	180.4	200.3	184.4	7.5%	7.9%	6.1	3.4%
		**20**	5	214.8	17.1	190.1	203.6	224.2	227.5	228.6	293.9	8.0%	8.4%	7.7	3.6%
	**PVA**	**1**	9	210.8	6.7	198.3	206.6	212.1	213.4	220.9	45.1	3.2%	3.3%	2.2	1.1%
		**3**	9	241.8	6.5	235.6	237.3	239.7	246.9	254.7	41.8	2.7%	2.7%	2.2	0.9%
		**5**	9	251.9	11.2	239.9	242.6	245.9	264.2	266.7	125.2	4.4%	4.6%	3.7	1.5%
		**8**	9	258.9	10.6	241.8	253.6	258.4	267.0	273.9	112.2	4.1%	4.2%	3.5	1.4%
		**10**	9	290.6	13.1	270.0	282.6	294.9	295.6	309.9	172.6	4.5%	4.6%	4.4	1.5%
		**15**	7	327.6	13.6	312.4	316.4	326.9	335.8	349.8	186.1	4.2%	4.3%	5.2	1.6%
**P05**	**None**	**3**	9	142.4	5.5	135.5	139.4	140.9	146.5	151.4	30.2	3.9%	4.0%	1.8	1.3%
		**5**	9	156.9	5.8	151.1	152.9	155.5	159.8	167.5	33.3	3.7%	3.8%	1.9	1.2%
		**8**	9	183.4	4.5	178.1	180.1	183.6	185.6	192.6	20.0	2.4%	2.5%	1.5	0.8%
		**10**	9	200.9	4.7	192.4	197.8	200.9	205.0	207.0	22.4	2.4%	2.4%	1.6	0.8%
		**15**	7	312.2	6.7	303.0	306.9	313.8	317.2	320.2	44.8	2.1%	2.2%	2.5	0.8%
	**PVA**	**1**	9	226.4	17.0	209.3	214.4	219.5	239.8	254.8	290.6	7.5%	7.7%	5.7	2.5%
		**3**	9	233.0	9.6	216.5	231.0	235.8	238.1	245.4	91.6	4.1%	4.2%	3.2	1.4%
		**5**	9	249.1	4.8	240.7	246.7	250.9	252.6	253.8	22.9	1.9%	2.0%	1.6	0.6%
		**8**	9	269.8	8.2	257.9	263.4	272.1	276.5	281.0	67.6	3.0%	3.1%	2.7	1.0%
		**10**	9	283.5	6.7	269.6	279.2	284.6	287.2	291.3	44.3	2.3%	2.4%	2.2	0.8%

**P06**	**None**	**1**	9	98.5	7.8	88.7	89.0	101.7	104.8	108.0	61.2	7.9%	8.2%	2.6	2.6%
		**3**	9	110.5	3.3	105.8	108.7	110.7	111.1	116.4	11.0	3.0%	3.1%	1.1	1.0%
		**5**	3	121.1	2.6	118.8	119.7	120.5	122.2	123.9	6.7	2.1%	2.3%	1.5	1.2%
		**8**	9	162.6	3.6	157.0	160.5	164.3	165.0	167.5	13.3	2.2%	2.3%	1.2	0.7%
		**10**	9	181.1	4.0	175.7	178.7	180.3	183.1	187.0	15.7	2.2%	2.2%	1.3	0.7%
		**15**	9	214.2	3.4	209.6	212.5	214.2	215.3	220.0	11.6	1.6%	1.6%	1.1	0.5%
	**PVA**	**1**	9	145.0	6.1	139.4	140.1	141.9	150.5	155.3	36.6	4.2%	4.3%	2.0	1.4%
		**3**	9	159.9	2.9	155.9	158.3	160.7	160.9	165.4	8.5	1.8%	1.9%	1.0	0.6%
		**5**	9	170.1	3.8	166.3	167.1	168.6	171.4	176.3	14.4	2.2%	2.3%	1.3	0.7%
		**8**	9	218.8	3.7	213.5	216.6	218.5	220.4	226.2	13.9	1.7%	1.8%	1.2	0.6%
		**10**	9	239.1	5.5	228.3	238.3	240.6	241.3	247.6	30.1	2.3%	2.4%	1.8	0.8%
		**15**	9	281.8	5.0	272.5	279.9	281.7	284.3	291.2	25.0	1.8%	1.8%	1.7	0.6%
**P07**	**None**	**1**	9	84.4	6.2	78.4	80.1	81.8	91.8	93.4	38.2	7.3%	7.5%	2.1	2.4%
		**3**	9	117.5	2.6	113.8	116.5	117.5	119.0	121.3	6.6	2.2%	2.3%	0.9	0.7%
		**5**	9	149.9	3.4	146.0	147.8	148.3	154.0	154.3	11.6	2.3%	2.3%	1.1	0.8%
		**8**	9	179.2	5.2	172.7	173.8	179.3	184.1	185.1	27.4	2.9%	3.0%	1.7	1.0%
		**10**	9	188.0	2.6	183.7	187.4	188.3	190.2	191.0	6.7	1.4%	1.4%	0.9	0.5%
		**15**	6	221.8	20.8	201.7	206.9	213.8	239.8	248.7	433.7	9.4%	9.8%	8.5	3.8%
	**PVA**	**1**	8	124.3	1.3	122.2	123.5	123.9	125.5	126.0	1.7	1.1%	1.1%	0.5	0.4%
		**3**	9	161.4	3.1	155.0	160.8	160.9	164.4	164.7	9.8	1.9%	2.0%	1.0	0.6%
		**5**	9	194.1	3.1	188.6	192.5	193.2	197.0	198.0	9.4	1.6%	1.6%	1.0	0.5%
		**8**	9	232.3	4.8	227.2	227.8	232.9	235.4	240.3	23.3	2.1%	2.1%	1.6	0.7%
		**10**	9	240.1	6.5	229.1	234.7	242.8	245.2	247.7	42.3	2.7%	2.8%	2.2	0.9%
		**15**	9	280.3	9.4	270.3	272.7	277.1	290.2	292.9	87.7	3.3%	3.4%	3.1	1.1%
		**20**	8	298.6	20.5	273.2	277.8	300.4	318.3	322.0	419.8	6.9%	7.1%	7.2	2.4%
**P08**	**None**	**1**	9	94.3	15.3	79.3	84.2	86.7	111.0	116.8	234.4	16.2%	16.7%	5.1	5.4%
		**3**	9	133.8	6.4	125.8	130.5	132.0	139.1	143.6	40.8	4.8%	4.9%	2.1	1.6%
		**5**	9	168.4	22.2	147.3	153.8	157.4	195.2	201.5	493.0	13.2%	13.6%	7.4	4.4%
		**8**	9	193.3	13.3	173.8	188.5	194.8	205.3	210.8	176.9	6.9%	7.1%	4.4	2.3%
		**10**	9	206.9	4.4	199.5	203.9	205.8	209.4	212.9	19.3	2.1%	2.2%	1.5	0.7%

		**15**	6	216.1	8.3	206.6	209.1	216.8	223.6	224.3	69.2	3.8%	4.0%	3.4	1.6%
	**PVA**	**5**	3	405.7	12.4	393.2	399.6	405.9	411.9	417.9	152.6	3.0%	3.3%	7.1	1.8%
		**10**	3	454.9	13.8	443.5	447.3	451.0	460.6	470.2	189.6	3.0%	3.3%	8.0	1.7%
**P09**	**None**	**1**	8	117.3	2.4	113.9	115.6	117.2	119.1	120.9	5.6	2.0%	2.1%	0.8	0.7%
		**3**	9	175.5	12.4	161.5	164.2	174.2	186.9	193.7	152.6	7.0%	7.2%	4.1	2.3%
		**5**	9	197.0	5.3	188.6	193.0	198.1	199.0	206.2	28.4	2.7%	2.8%	1.8	0.9%
		**8**	9	224.1	14.1	203.8	213.8	223.3	236.8	240.7	198.7	6.3%	6.5%	4.7	2.1%
		**10**	8	241.7	6.4	230.4	238.2	242.3	245.3	251.4	40.4	2.6%	2.7%	2.2	0.9%
	**PVA**	**1**	9	157.9	7.9	148.0	152.8	157.2	164.8	171.1	62.6	5.0%	5.1%	2.6	1.7%
		**3**	9	191.5	5.9	183.1	187.7	190.7	194.0	203.4	34.5	3.1%	3.2%	2.0	1.0%
		**5**	9	227.2	8.1	217.3	221.8	224.8	231.9	238.9	65.5	3.6%	3.7%	2.7	1.2%
		**8**	9	258.9	12.2	238.8	252.2	262.4	267.5	272.2	148.4	4.7%	4.8%	4.1	1.6%
		**10**	9	279.4	11.5	259.2	269.5	283.8	288.6	292.2	131.3	4.1%	4.2%	3.8	1.4%
		**15**	8	347.3	34.0	294.7	326.5	346.2	364.5	395.1	1155.2	9.8%	10.1%	12.0	3.5%
**P10**	**None**	**1**	9	79.1	2.3	75.8	77.2	80.4	80.7	82.0	5.3	2.9%	3.0%	0.8	1.0%
		**3**	9	127.0	6.3	117.6	121.3	129.9	131.1	135.5	40.1	5.0%	5.1%	2.1	1.7%
		**5**	9	147.7	6.4	140.9	142.0	146.6	149.8	160.6	41.4	4.4%	4.5%	2.1	1.5%
		**8**	8	182.9	2.5	180.4	181.3	181.7	184.5	187.1	6.1	1.3%	1.4%	0.9	0.5%
		**10**	9	196.9	9.4	181.8	193.3	198.5	200.1	213.5	88.2	4.8%	4.9%	3.1	1.6%
	**PVA**	**1**	9	144.9	8.1	137.5	139.0	140.5	154.1	156.9	65.0	5.6%	5.7%	2.7	1.9%
		**3**	9	168.5	4.9	159.0	164.6	170.7	172.1	172.5	23.7	2.9%	3.0%	1.6	1.0%
		**5**	9	205.4	9.8	187.5	197.0	211.5	213.1	214.1	96.6	4.8%	4.9%	3.3	1.6%
		**8**	9	226.8	16.3	201.5	209.4	235.9	238.1	242.8	266.3	7.2%	7.4%	5.4	2.4%
		**10**	9	250.0	8.7	240.1	241.9	248.8	256.0	265.6	75.8	3.5%	3.6%	2.9	1.2%
**P11**	**None**	**1**	9	129.3	3.6	125.2	126.7	127.5	131.5	136.2	13.0	2.8%	2.9%	1.2	0.9%
		**3**	9	165.0	3.0	160.2	162.9	165.4	166.4	170.1	8.9	1.8%	1.9%	1.0	0.6%
		**5**	9	198.4	7.6	189.1	191.4	198.8	203.9	210.4	57.4	3.8%	3.9%	2.5	1.3%
		**8**	9	215.7	11.2	204.2	207.8	211.5	217.9	234.0	125.6	5.2%	5.3%	3.7	1.7%
		**10**	6	249.5	13.9	230.6	242.8	247.1	256.9	270.5	194.3	5.6%	5.8%	5.7	2.3%
	**PVA**	**1**	9	175.7	5.2	169.1	173.0	174.2	180.2	184.6	26.6	2.9%	3.0%	1.7	1.0%

		**3**	9	196.4	6.2	185.4	192.0	198.9	200.7	202.4	38.3	3.2%	3.2%	2.1	1.1%
		**5**	9	226.7	12.5	213.6	218.1	222.9	234.5	249.5	155.8	5.5%	5.7%	4.2	1.8%
		**8**	9	249.5	11.5	236.9	240.6	247.9	252.4	272.9	131.7	4.6%	4.7%	3.8	1.5%
		**10**	8	267.1	4.2	261.5	264.4	265.8	270.3	273.9	17.8	1.6%	1.6%	1.5	0.6%
		**15**	7	343.1	23.8	305.6	329.9	338.7	364.6	368.3	565.2	6.9%	7.2%	9.0	2.6%
**P12**	**None**	**5**	9	219.5	4.6	212.5	216.1	219.7	222.3	227.4	21.0	2.1%	2.1%	1.5	0.7%
		**8**	9	221.5	22.9	195.9	209.0	210.5	248.4	255.5	526.2	10.4%	10.6%	7.6	3.5%
		**10**	5	226.4	8.3	214.4	224.5	225.1	231.7	236.4	69.3	3.7%	3.9%	3.7	1.6%
		**15**	4	245.7	41.2	204.5	223.6	238.2	260.3	301.8	1698.3	16.8%	17.8%	20.6	8.4%
	**PVA**	**1**	9	226.1	7.7	211.3	222.2	227.3	230.0	238.7	59.3	3.4%	3.5%	2.6	1.1%
		**3**	9	248.3	3.7	242.7	245.1	248.7	250.4	253.1	14.1	1.5%	1.6%	1.2	0.5%
		**5**	9	264.3	5.9	254.7	260.1	265.2	268.8	273.2	35.3	2.2%	2.3%	2.0	0.7%
		**8**	9	278.0	5.0	269.1	275.5	277.5	281.3	286.9	25.0	1.8%	1.8%	1.7	0.6%
		**10**	9	321.3	9.4	309.4	316.1	322.0	324.2	336.0	89.3	2.9%	3.0%	3.1	1.0%
		**15**	6	410.9	38.0	359.3	387.7	415.0	425.9	468.1	1447.0	9.3%	9.6%	15.5	3.8%
**P13**	**None**	**1**	9	122.3	6.0	115.6	117.8	120.9	128.7	130.9	35.5	4.9%	5.0%	2.0	1.6%
		**3**	9	153.1	7.1	143.9	145.3	153.9	157.2	161.9	50.0	4.6%	4.7%	2.4	1.5%
		**5**	9	171.3	2.1	167.3	169.8	171.4	173.0	173.7	4.6	1.3%	1.3%	0.7	0.4%
		**8**	9	212.6	13.3	198.3	201.9	212.3	219.6	238.9	175.9	6.2%	6.4%	4.4	2.1%
		**10**	6	224.1	0.9	222.8	223.8	224.2	224.6	225.3	0.7	0.4%	0.4%	0.3	0.2%
	**PVA**	**1**	9	205.8	3.0	202.3	203.4	204.5	209.0	209.4	8.8	1.4%	1.5%	1.0	0.5%
		**3**	9	233.4	5.3	225.0	230.4	234.7	235.9	243.1	28.5	2.3%	2.3%	1.8	0.8%
		**5**	8	260.5	5.6	254.6	256.5	258.5	265.2	269.5	30.9	2.1%	2.2%	2.0	0.8%
		**8**	6	288.6	4.9	282.8	285.3	288.7	290.3	296.5	23.7	1.7%	1.8%	2.0	0.7%
		**10**	8	308.6	6.4	300.5	304.4	307.2	312.0	320.5	41.5	2.1%	2.2%	2.3	0.7%
**P14**	**None**	**1**	9	105.5	3.5	100.0	102.4	107.1	107.9	109.7	12.3	3.3%	3.4%	1.2	1.1%
		**3**	9	123.4	2.4	120.4	121.4	124.2	125.0	126.9	5.8	2.0%	2.0%	0.8	0.7%
		**5**	9	165.2	31.9	139.9	144.5	146.0	202.6	216.4	1018.1	19.3%	19.9%	10.6	6.4%
		**8**	9	181.8	5.6	172.8	178.0	182.7	185.6	189.6	31.0	3.1%	3.1%	1.9	1.0%
		**10**	9	189.6	7.3	174.7	186.9	187.4	195.0	198.9	53.5	3.9%	4.0%	2.4	1.3%

		**15**	6	236.2	10.4	226.5	228.1	233.5	241.9	253.0	109.1	4.4%	4.6%	4.3	1.8%
		**20**	4	249.1	33.0	219.8	233.5	240.1	255.6	296.5	1092.2	13.3%	14.1%	16.5	6.6%
	**PVA**	**1**	9	187.5	5.8	180.0	180.8	190.7	191.7	194.2	33.4	3.1%	3.2%	1.9	1.0%
		**3**	9	207.2	4.4	201.1	203.5	207.8	210.6	213.7	19.1	2.1%	2.2%	1.5	0.7%
		**5**	9	228.2	6.2	220.5	223.3	227.2	233.6	238.8	38.9	2.7%	2.8%	2.1	0.9%
		**8**	6	247.3	4.3	242.4	243.5	247.8	251.0	251.7	18.2	1.7%	1.8%	1.7	0.7%
		**10**	8	258.8	3.3	253.3	256.1	259.9	261.4	262.2	10.6	1.3%	1.3%	1.2	0.4%
		**15**	8	304.7	2.5	299.9	304.0	304.9	306.0	307.8	6.4	0.8%	0.9%	0.9	0.3%
**P15**	**None**	**1**	9	130.9	8.6	122.6	124.9	126.2	141.0	142.8	73.9	6.6%	6.8%	2.9	2.2%
		**3**	9	145.3	5.6	136.1	140.2	146.2	148.1	153.6	31.5	3.9%	4.0%	1.9	1.3%
		**5**	9	161.8	4.7	155.3	158.1	162.6	163.5	170.6	22.3	2.9%	3.0%	1.6	1.0%
		**8**	8	183.9	3.0	179.8	181.4	183.8	186.6	187.9	9.2	1.6%	1.7%	1.1	0.6%
		**10**	9	196.2	6.1	185.9	192.9	198.7	200.5	202.8	37.5	3.1%	3.2%	2.0	1.0%
		**15**	9	215.9	4.6	210.0	211.8	216.3	220.2	221.8	21.5	2.1%	2.2%	1.5	0.7%
		**20**	8	253.3	12.4	229.0	250.4	256.1	262.1	265.4	154.5	4.9%	5.1%	4.4	1.7%
	**PVA**	**1**	9	206.4	9.3	195.2	198.2	206.2	212.0	222.7	86.8	4.5%	4.6%	3.1	1.5%
		**3**	9	238.9	10.0	221.5	232.2	241.7	246.7	251.0	99.9	4.2%	4.3%	3.3	1.4%
		**5**	9	256.1	6.1	245.7	253.2	256.0	261.3	263.1	37.0	2.4%	2.4%	2.0	0.8%
		**8**	6	267.0	1.4	264.8	266.2	267.5	268.0	268.5	2.1	0.5%	0.6%	0.6	0.2%
		**10**	9	289.4	8.1	277.7	282.8	292.6	294.0	302.4	65.6	2.8%	2.9%	2.7	0.9%
		**15**	9	306.0	9.8	290.7	302.3	309.9	311.0	321.9	96.6	3.2%	3.3%	3.3	1.1%
		**20**	7	354.0	12.2	332.9	349.3	354.8	360.9	370.1	148.1	3.4%	3.6%	4.6	1.3%
		**30**	5	362.7	22.8	338.8	346.8	356.8	375.6	395.3	522.0	6.3%	6.6%	10.2	2.8%
**P16**	**None**	**1**	9	104.2	6.3	96.0	97.1	106.0	110.1	111.4	39.8	6.1%	6.2%	2.1	2.0%
		**3**	9	125.6	3.6	118.3	124.4	127.3	127.9	128.6	12.7	2.8%	2.9%	1.2	0.9%
		**5**	9	147.9	3.6	142.6	145.2	148.2	151.0	153.1	13.1	2.4%	2.5%	1.2	0.8%
		**8**	9	190.3	2.9	187.0	188.4	190.1	190.8	196.3	8.2	1.5%	1.5%	1.0	0.5%
		**10**	9	204.3	3.9	197.4	204.4	205.2	206.7	209.0	15.1	1.9%	2.0%	1.3	0.6%
		**15**	6	230.5	5.9	222.5	226.8	231.1	233.4	239.0	35.2	2.6%	2.7%	2.4	1.1%
	**PVA**	**1**	9	153.5	3.3	149.5	150.5	153.2	155.3	159.7	11.0	2.2%	2.2%	1.1	0.7%

		**3**	9	185.7	3.6	180.4	182.9	186.7	189.5	190.0	13.2	2.0%	2.0%	1.2	0.7%
		**5**	9	205.2	13.0	191.6	197.3	201.0	209.0	227.5	168.0	6.3%	6.5%	4.3	2.1%
		**8**	9	246.5	6.3	238.1	241.1	246.3	248.3	258.4	39.6	2.6%	2.6%	2.1	0.9%
		**10**	9	275.5	6.0	266.0	271.2	275.1	280.5	285.5	36.1	2.2%	2.2%	2.0	0.7%
		**15**	9	294.4	7.9	280.8	292.3	295.1	297.0	309.5	62.7	2.7%	2.8%	2.6	0.9%
		**20**	3	342.9	19.1	329.8	332.0	334.1	349.5	364.8	364.3	5.6%	6.0%	11.0	3.2%
**P17**	**None**	**5**	9	185.8	6.5	177.5	182.2	184.2	191.1	197.2	41.7	3.5%	3.6%	2.2	1.2%
		**8**	8	212.4	5.1	204.1	209.9	212.1	214.7	221.6	26.0	2.4%	2.5%	1.8	0.8%
		**10**	7	214.3	15.9	194.6	204.5	216.9	218.4	242.7	252.5	7.4%	7.7%	6.0	2.8%
	**PVA**	**1**	9	214.8	7.8	201.5	210.2	216.6	218.2	226.8	61.3	3.6%	3.7%	2.6	1.2%
		**3**	9	227.7	7.4	216.7	223.2	227.5	231.2	242.5	55.3	3.3%	3.4%	2.5	1.1%
		**5**	9	253.9	7.5	244.0	249.5	250.6	259.4	264.7	55.6	2.9%	3.0%	2.5	1.0%
		**8**	8	269.3	3.4	264.9	266.4	269.8	272.4	272.8	11.5	1.3%	1.3%	1.2	0.4%
		**10**	9	285.7	6.2	277.5	281.9	283.6	290.1	296.4	38.4	2.2%	2.2%	2.1	0.7%
		**15**	8	304.5	12.4	288.7	297.9	302.5	313.1	323.2	154.8	4.1%	4.2%	4.4	1.4%
**P18**	**None**	**8**	8	255.5	16.9	238.2	244.3	250.0	264.4	282.8	285.0	6.6%	6.8%	6.0	2.3%
		**10**	3	419.6	27.7	390.5	406.7	422.8	434.2	445.6	766.5	6.6%	7.1%	16.0	3.8%
	**PVA**	**1**	9	282.8	7.7	269.5	278.8	280.0	288.0	295.7	59.3	2.7%	2.8%	2.6	0.9%
		**3**	9	323.3	4.6	317.6	319.5	321.7	325.7	331.2	21.6	1.4%	1.5%	1.5	0.5%
		**5**	9	336.4	28.8	302.4	304.4	333.9	362.2	377.4	831.1	8.6%	8.8%	9.6	2.9%
		**8**	9	355.8	8.1	340.1	352.6	355.9	359.1	368.9	65.0	2.3%	2.3%	2.7	0.8%
		**10**	9	375.4	17.0	344.5	364.8	377.1	384.7	404.1	290.5	4.5%	4.7%	5.7	1.5%
**P19**	**None**	**1**	9	100.9	7.3	91.0	91.7	104.5	106.5	108.1	54.0	7.3%	7.5%	2.4	2.4%
		**3**	9	118.8	4.2	110.9	115.8	119.8	121.6	124.6	18.0	3.6%	3.7%	1.4	1.2%
		**5**	9	137.7	4.3	130.3	136.2	138.8	140.8	142.3	18.1	3.1%	3.2%	1.4	1.0%
		**8**	9	160.4	7.3	151.6	155.7	157.9	167.9	170.4	53.4	4.6%	4.7%	2.4	1.5%
		**10**	9	182.3	9.0	169.8	175.2	183.8	187.2	195.1	81.2	4.9%	5.1%	3.0	1.6%
		**15**	7	219.5	8.4	204.5	216.8	222.4	222.6	231.1	71.1	3.8%	4.0%	3.2	1.5%
	**PVA**	**1**	8	162.4	2.8	158.2	160.9	161.8	164.5	166.5	8.1	1.8%	1.8%	1.0	0.6%
		**3**	9	176.5	10.7	160.6	166.8	179.1	181.6	191.5	114.4	6.1%	6.2%	3.6	2.0%

		**5**	9	196.8	4.4	189.8	194.9	196.0	198.7	203.8	19.0	2.2%	2.3%	1.5	0.7%
		**8**	9	222.8	3.7	216.6	220.9	223.6	225.1	228.1	13.5	1.6%	1.7%	1.2	0.5%
		**10**	9	232.5	10.6	216.2	228.0	233.3	238.3	250.0	111.5	4.5%	4.7%	3.5	1.5%
		**15**	9	274.1	9.0	263.5	265.8	274.5	280.8	289.7	81.6	3.3%	3.4%	3.0	1.1%
**P20**	**None**	**3**	9	125.2	0.6	124.5	124.8	125.1	125.4	126.2	0.4	0.5%	0.5%	0.2	0.2%
		**5**	9	147.2	5.1	139.6	144.0	147.4	152.6	153.9	25.8	3.5%	3.5%	1.7	1.2%
		**8**	9	175.4	4.1	171.4	172.0	174.0	177.8	181.5	16.5	2.3%	2.4%	1.4	0.8%
		**10**	9	182.6	5.5	174.9	179.9	181.8	186.2	192.6	30.0	3.0%	3.1%	1.8	1.0%
		**15**	9	211.5	5.7	203.2	206.8	212.5	213.6	220.6	32.6	2.7%	2.8%	1.9	0.9%
	**PVA**	**1**	9	195.3	7.0	185.4	188.4	198.5	199.5	205.8	49.5	3.6%	3.7%	2.3	1.2%
		**3**	9	211.0	3.5	206.0	208.3	210.6	212.9	216.8	12.1	1.6%	1.7%	1.2	0.5%
		**5**	9	230.8	5.3	220.9	229.2	230.7	234.3	237.7	28.3	2.3%	2.4%	1.8	0.8%
		**8**	9	249.3	7.8	236.1	245.2	248.8	252.7	260.9	60.4	3.1%	3.2%	2.6	1.0%
		**10**	9	258.9	5.2	252.6	254.8	259.6	261.7	267.0	27.1	2.0%	2.1%	1.7	0.7%
		**15**	9	292.1	17.8	265.7	285.3	286.6	307.7	324.0	318.6	6.1%	6.3%	5.9	2.0%
**P21**	**None**	**5**	9	170.2	15.8	155.8	157.0	162.2	189.5	193.0	249.0	9.3%	9.5%	5.3	3.1%
		**8**	9	171.0	3.7	164.6	169.8	171.1	173.3	177.1	13.7	2.2%	2.2%	1.2	0.7%
		**10**	9	253.0	5.2	247.9	249.4	250.3	256.0	261.8	26.5	2.0%	2.1%	1.7	0.7%
		**15**	4	339.6	20.4	319.3	324.1	338.3	353.8	362.3	416.1	6.0%	6.4%	10.2	3.0%
	**PVA**	**1**	9	194.9	5.4	187.9	188.2	196.8	197.2	201.2	29.2	2.8%	2.8%	1.8	0.9%
		**3**	9	213.5	3.9	209.8	210.5	213.1	214.6	221.4	15.4	1.8%	1.9%	1.3	0.6%
		**5**	9	224.3	3.4	217.9	222.8	225.6	225.7	229.8	11.5	1.5%	1.6%	1.1	0.5%
		**8**	9	238.9	2.5	234.2	237.6	239.5	240.7	242.0	6.4	1.1%	1.1%	0.8	0.4%
		**10**	9	284.7	11.0	266.1	280.4	288.0	288.6	304.4	120.9	3.9%	4.0%	3.7	1.3%
**P22**	**None**	**5**	9	167.8	5.1	160.9	162.6	168.3	172.4	174.3	26.2	3.0%	3.1%	1.7	1.0%
		**8**	9	182.4	4.6	175.7	178.6	184.3	186.6	187.3	21.3	2.5%	2.6%	1.5	0.8%
		**10**	9	207.3	5.0	200.7	203.3	208.1	209.4	216.2	25.4	2.4%	2.5%	1.7	0.8%
		**15**	7	214.7	16.9	182.7	209.7	220.0	225.5	229.7	286.6	7.9%	8.2%	6.4	3.0%
	**PVA**	**1**	9	192.6	4.6	186.9	189.5	190.8	197.1	199.7	21.5	2.4%	2.5%	1.5	0.8%
		**3**	9	213.6	4.4	206.7	211.6	214.8	215.4	220.1	19.0	2.0%	2.1%	1.5	0.7%

		**5**	9	228.3	6.3	222.0	223.4	226.0	232.5	239.9	40.3	2.8%	2.9%	2.1	0.9%
		**8**	9	261.8	9.8	248.8	255.4	258.3	272.1	276.1	96.9	3.8%	3.9%	3.3	1.3%
		**10**	9	260.3	6.6	248.1	257.5	261.9	265.0	268.5	43.0	2.5%	2.6%	2.2	0.8%
		**15**	8	298.7	9.2	287.3	292.5	298.4	303.9	312.8	85.1	3.1%	3.2%	3.3	1.1%
		**20**	7	313.2	27.8	266.0	302.6	322.8	327.5	343.2	770.4	8.9%	9.2%	10.5	3.3%
**P23**	**None**	**5**	9	224.4	3.2	218.9	221.4	225.7	225.9	228.3	10.1	1.4%	1.5%	1.1	0.5%
		**8**	9	238.9	11.5	223.1	225.3	243.1	247.0	252.7	132.5	4.8%	5.0%	3.8	1.6%
		**10**	8	258.7	14.0	243.2	247.6	254.7	271.8	278.3	196.1	5.4%	5.6%	5.0	1.9%
		**15**	9	262.7	19.7	231.5	256.8	258.4	273.6	295.4	387.4	7.5%	7.7%	6.6	2.5%
**P24**	**None**	**3**	9	84.1	1.8	81.9	82.7	83.4	85.2	86.6	3.2	2.1%	2.2%	0.6	0.7%
		**5**	9	107.1	2.2	103.6	105.6	107.8	108.2	110.4	4.7	2.0%	2.1%	0.7	0.7%
		**8**	6	114.5	4.2	110.6	111.1	113.5	117.2	120.9	18.0	3.7%	3.9%	1.7	1.5%
		**10**	9	136.4	6.2	126.8	130.1	136.9	140.1	144.3	38.2	4.5%	4.7%	2.1	1.5%
		**15**	6	147.7	3.6	142.5	146.2	147.1	150.0	152.4	12.7	2.4%	2.5%	1.5	1.0%
		**20**	9	173.2	3.9	167.6	171.2	172.9	175.8	180.7	15.4	2.3%	2.3%	1.3	0.8%
		**30**	8	209.8	2.7	205.1	209.1	209.9	210.3	214.9	7.5	1.3%	1.3%	1.0	0.5%
	**PVA**	**1**	9	139.5	2.2	135.8	137.7	140.8	141.4	141.9	5.0	1.6%	1.7%	0.7	0.5%
		**3**	9	147.5	4.3	142.4	143.9	147.7	150.2	155.8	18.2	2.9%	3.0%	1.4	1.0%
		**5**	9	166.4	3.2	161.4	163.5	167.2	168.8	171.1	10.0	1.9%	2.0%	1.1	0.6%
		**8**	9	186.9	3.6	181.9	183.6	187.4	190.3	191.4	13.2	1.9%	2.0%	1.2	0.6%
		**10**	9	196.5	6.0	186.0	194.4	195.1	200.8	204.5	36.2	3.1%	3.1%	2.0	1.0%
		**15**	9	216.0	3.5	210.5	213.5	216.1	219.0	220.4	12.1	1.6%	1.7%	1.2	0.5%
		**20**	9	236.7	4.7	228.7	233.6	235.9	239.9	244.3	22.0	2.0%	2.0%	1.6	0.7%
		**30**	9	265.4	8.2	257.1	257.4	263.1	269.7	279.2	67.7	3.1%	3.2%	2.7	1.0%
**P25**	**None**	**1**	6	183.7	5.3	177.7	179.6	183.8	186.4	191.7	27.8	2.9%	3.0%	2.2	1.2%
		**3**	6	247.3	20.2	224.4	229.2	248.7	262.8	271.8	408.3	8.2%	8.5%	8.2	3.3%
		**5**	6	259.1	25.5	231.6	238.2	257.7	282.4	285.2	648.7	9.8%	10.2%	10.4	4.0%
		**8**	4	344.1	45.8	310.1	311.5	329.2	361.8	408.0	2093.8	13.3%	14.1%	22.9	6.6%
		**10**	6	305.6	28.8	273.4	284.9	300.1	325.6	346.0	829.6	9.4%	9.8%	11.8	3.8%
		**20**	3	337.0	33.9	308.0	318.4	328.8	351.6	374.3	1149.8	10.1%	10.9%	19.6	5.8%

	PVA	1	9	240.0	14.3	226.9	230.7	233.1	251.3	264.6	205.7	6.0%	6.1%	4.8	2.0%
		3	9	283.4	4.5	274.3	281.6	283.5	286.6	288.3	20.2	1.6%	1.6%	1.5	0.5%
		5	9	335.7	15.4	314.4	323.4	335.8	347.1	360.3	237.5	4.6%	4.7%	5.1	1.5%
		8	9	409.3	19.9	370.9	406.5	412.9	417.5	435.5	396.6	4.9%	5.0%	6.6	1.6%
		10	3	429.3	24.5	408.7	415.7	422.7	439.6	456.4	601.2	5.7%	6.2%	14.2	3.3%
P26	None	1	8	183.0	7.0	174.3	177.5	182.6	186.7	193.9	48.3	3.8%	3.9%	2.5	1.3%
		3	6	211.3	5.6	204.5	208.7	211.1	211.8	221.3	31.6	2.7%	2.8%	2.3	1.1%
		5	7	222.2	5.6	212.3	220.8	222.8	223.9	230.8	31.4	2.5%	2.6%	2.1	1.0%
		8	6	227.9	12.4	211.4	219.3	229.0	234.6	245.3	154.0	5.4%	5.7%	5.1	2.2%
		10	4	266.1	11.5	253.1	258.1	267.7	275.7	276.1	132.3	4.3%	4.6%	5.8	2.2%
	PVA	1	9	270.4	6.9	257.2	269.0	271.1	276.4	279.0	47.7	2.6%	2.6%	2.3	0.9%
		3	9	306.5	9.7	294.5	297.9	306.2	314.5	320.8	94.2	3.2%	3.3%	3.2	1.1%
		5	8	311.4	10.8	297.0	301.8	313.8	319.0	326.1	116.3	3.5%	3.6%	3.8	1.2%
		8	9	319.4	7.9	310.6	315.5	317.3	323.1	335.9	62.5	2.5%	2.5%	2.6	0.8%
		10	7	366.7	9.8	355.2	357.7	369.6	372.9	380.8	96.6	2.7%	2.8%	3.7	1.0%
		15	6	394.6	19.0	366.3	382.9	398.8	408.8	414.2	359.4	4.8%	5.0%	7.7	2.0%
P27	None	1	8	198.2	13.2	175.3	190.3	203.6	205.8	214.7	173.6	6.6%	6.9%	4.7	2.3%
		3	8	203.1	6.6	192.4	200.8	201.6	206.2	215.0	43.2	3.2%	3.3%	2.3	1.1%
		5	9	208.7	9.1	192.6	206.5	209.0	217.6	218.9	82.9	4.4%	4.5%	3.0	1.5%
		8	9	247.5	8.9	234.6	241.6	246.5	250.9	265.9	79.7	3.6%	3.7%	3.0	1.2%
		10	9	263.4	8.6	251.8	254.2	264.6	269.7	273.4	73.6	3.3%	3.3%	2.9	1.1%
	PVA	1	9	252.5	12.7	233.7	239.4	256.4	262.8	267.0	160.8	5.0%	5.2%	4.2	1.7%
		3	9	286.2	8.4	278.3	279.4	284.4	292.5	302.2	70.5	2.9%	3.0%	2.8	1.0%
		5	9	302.6	5.1	292.5	300.0	304.2	304.5	309.1	26.3	1.7%	1.7%	1.7	0.6%
		8	9	340.4	11.6	321.1	334.7	342.0	343.3	361.2	134.0	3.4%	3.5%	3.9	1.1%
		10	9	372.1	6.9	359.7	368.6	372.2	377.0	383.1	48.3	1.9%	1.9%	2.3	0.6%
P28	None	8	8	222.6	27.6	189.5	203.9	222.3	232.9	274.7	760.1	12.4%	12.8%	9.7	4.4%
		10	9	238.1	13.8	216.8	226.7	240.4	246.4	256.5	190.1	5.8%	6.0%	4.6	1.9%
	PVA	1	9	241.3	6.0	233.3	237.0	241.5	245.5	251.0	36.6	2.5%	2.6%	2.0	0.8%
		3	9	261.1	9.2	248.5	254.8	260.2	267.5	277.4	84.0	3.5%	3.6%	3.1	1.2%

		**5**	9	296.8	14.5	278.5	287.4	289.7	312.5	314.7	211.4	4.9%	5.0%	4.8	1.6%
		**8**	9	313.8	10.5	302.1	306.6	308.6	318.9	330.5	111.0	3.4%	3.5%	3.5	1.1%
		**10**	9	348.1	12.1	332.7	339.9	347.2	352.3	373.1	145.3	3.5%	3.6%	4.0	1.2%
		**15**	7	384.2	9.8	370.8	377.0	382.8	393.0	395.8	96.5	2.6%	2.6%	3.7	1.0%
**P29**	**None**	**5**	3	265.8	9.7	255.8	261.1	266.4	270.8	275.1	93.4	3.6%	3.9%	5.6	2.1%
		**8**	9	278.5	22.4	250.5	264.7	267.7	301.3	310.4	501.5	8.0%	8.3%	7.5	2.7%
		**10**	9	369.1	16.0	344.5	365.6	367.2	383.3	390.7	255.3	4.3%	4.4%	5.3	1.4%
	**PVA**	**1**	9	204.5	15.3	182.6	187.4	207.3	216.6	224.2	234.4	7.5%	7.7%	5.1	2.5%
		**3**	9	243.9	6.1	235.6	238.4	243.7	249.7	251.9	37.6	2.5%	2.6%	2.0	0.8%
		**5**	9	291.4	18.0	256.6	281.4	297.8	303.4	308.0	325.6	6.2%	6.4%	6.0	2.1%
		**8**	9	346.0	14.3	325.3	339.5	341.1	357.6	368.2	203.5	4.1%	4.2%	4.8	1.4%
		**10**	5	455.1	8.1	444.3	451.1	454.2	461.8	464.2	65.3	1.8%	1.9%	3.6	0.8%
**P30**	**None**	**1**	9	196.4	9.4	182.2	191.2	193.2	202.6	210.7	88.0	4.8%	4.9%	3.1	1.6%
		**3**	9	234.7	9.0	222.2	229.4	235.9	241.1	249.4	81.3	3.8%	3.9%	3.0	1.3%
		**5**	9	267.1	5.3	257.0	263.6	267.5	272.1	272.7	27.9	2.0%	2.0%	1.8	0.7%
	**PVA**	**1**	9	227.1	9.9	210.2	219.7	228.6	235.7	238.7	97.9	4.4%	4.5%	3.3	1.5%
		**3**	9	257.5	5.3	249.8	252.4	258.6	260.1	267.2	28.1	2.1%	2.1%	1.8	0.7%
		**5**	7	302.9	8.6	291.3	295.5	306.2	309.0	313.6	74.1	2.8%	2.9%	3.3	1.1%
**P31**	**None**	**1**	9	98.5	5.2	91.6	94.5	98.3	102.0	106.4	27.2	5.3%	5.4%	1.7	1.8%
		**3**	9	111.0	6.1	104.5	106.7	108.6	117.0	122.1	37.4	5.5%	5.7%	2.0	1.8%
		**5**	9	115.7	2.7	113.0	113.4	115.9	116.9	121.1	7.4	2.4%	2.4%	0.9	0.8%
		**8**	9	134.2	1.4	131.5	133.6	134.4	134.6	136.0	2.1	1.1%	1.1%	0.5	0.4%
		**10**	9	139.1	2.4	136.6	137.5	138.4	139.6	143.5	5.7	1.7%	1.8%	0.8	0.6%
		**15**	9	155.8	6.2	145.2	154.2	157.6	160.9	161.7	37.9	3.9%	4.1%	2.1	1.3%
		**20**	9	173.5	4.7	163.7	171.5	173.3	177.4	178.7	22.4	2.7%	2.8%	1.6	0.9%
		**30**	9	205.1	10.5	191.3	194.8	207.0	214.3	220.0	110.6	5.1%	5.3%	3.5	1.7%
	**PVA**	**1**	9	174.2	3.4	170.1	171.3	173.6	177.8	179.2	11.8	2.0%	2.0%	1.1	0.7%
		**3**	8	188.4	2.7	185.1	186.4	188.5	189.7	193.5	7.5	1.4%	1.5%	1.0	0.5%
		**5**	9	193.9	6.1	181.9	189.3	197.1	197.6	200.5	37.6	3.2%	3.3%	2.0	1.1%
		**8**	7	207.9	1.5	206.2	206.8	207.8	208.5	210.5	2.2	0.7%	0.7%	0.6	0.3%

		**10**	9	210.4	3.1	205.4	208.8	210.5	211.0	216.1	9.9	1.5%	1.5%	1.0	0.5%
		**15**	9	224.5	6.6	216.9	220.3	222.0	227.2	235.2	43.2	2.9%	3.0%	2.2	1.0%
		**20**	8	236.9	6.2	229.9	231.9	236.2	241.6	245.3	38.9	2.6%	2.7%	2.2	0.9%
		**30**	9	269.9	4.5	263.2	266.0	271.6	273.2	274.8	20.1	1.7%	1.7%	1.5	0.6%
**P32**	**None**	**5**	8	133.9	1.4	131.5	133.3	133.7	134.5	136.3	1.9	1.0%	1.1%	0.5	0.4%
		**8**	9	150.4	10.5	140.5	142.3	145.0	161.0	167.3	110.5	7.0%	7.2%	3.5	2.3%
		**10**	9	152.6	2.0	149.4	150.3	153.7	153.8	154.6	4.2	1.3%	1.4%	0.7	0.4%
		**15**	9	161.7	3.8	155.7	159.7	161.5	163.4	167.4	14.2	2.3%	2.4%	1.3	0.8%
		**20**	6	182.5	4.8	176.0	179.2	183.0	186.4	187.4	23.0	2.6%	2.7%	2.0	1.1%
		**30**	9	202.5	6.6	191.5	198.9	206.6	207.5	207.9	43.0	3.2%	3.3%	2.2	1.1%
	**PVA**	**1**	9	221.0	5.7	211.2	220.6	222.1	225.0	226.5	32.1	2.6%	2.6%	1.9	0.9%
		**3**	6	322.0	10.3	307.1	315.6	325.4	326.4	335.2	106.4	3.2%	3.3%	4.2	1.3%
		**5**	5	281.8	7.3	274.0	274.8	284.2	285.0	291.1	53.1	2.6%	2.7%	3.3	1.2%
		**8**	7	265.6	7.2	256.4	260.7	265.7	268.8	277.9	52.0	2.7%	2.8%	2.7	1.0%
		**10**	8	265.2	8.1	253.5	259.8	266.0	270.9	275.9	65.0	3.0%	3.1%	2.9	1.1%
		**15**	7	248.9	5.5	241.8	245.2	247.3	253.6	255.8	30.3	2.2%	2.3%	2.1	0.8%
		**20**	9	254.3	9.5	242.0	244.5	255.8	262.7	266.7	89.3	3.7%	3.8%	3.2	1.2%
		**30**	9	275.7	8.9	261.1	270.6	277.0	282.9	288.9	78.5	3.2%	3.3%	3.0	1.1%

An explanation for reduced cyclic representations

Given a linear infinite graph $G = (N, E)$, with $N = [n_1, n_2 \dots n_n]$, $|N| = \infty$ and $E_k = (n_k, n_{k+1})$ where all nodes N hold a feature vector with m features $F_n = [f_1^n, f_2^n, \dots, f_m^n]$ where $f \in \mathbb{R}$. If the feature vectors repeat ever p Nodes ($F_{n+p} = F_n$), a simplified cyclic representation of the graph $G' = (N', E')$, with $N' = [n_1, n_2 \dots n_p]$, $|N'| = p$, $E_k = (n_k, n_{k+1})$ and $E_p = (n_p, n_1)$ can be created.

In the graph convolution for each node, a new feature vector F_n^{GC} is created, whose values are calculated by passing the combined feature vectors of the previous, the next, and the node itself (*via* a function c, *e.g.*, averaging $c(a, b, c) = \frac{a+b+c}{3}$) multiplied with a weight vector-matrix W into an activation function:

$$F_n^C = \sigma(c(F_{n-1}, F_{n,} F_{n+1}) * W) \quad (1)$$

Since W is independently from the nodes, $F_n^{GC} = F_{n+p}^{GC}$ for the infinite periodic graph representation. For the reduced cyclic representation, the same is true, since the feature vector prior to the first equals the last one due to the cyclic connection of the first and the last node $F_{-1} = F_p$ and, thus, equation 1 gives the same results for the circular and the linear representation.

As a practical proof of the concept, a simple graph convolutional network (see notebooks/circular_proof.ipynb in software repository) was designed and the resulting output was a 128 values long array. The network was not trained and all weights and biases were left at the randomly initiated state. The resulting fingerprints have no intrinsic meaning, but the Euclidian distance between two fingerprints correlates with the similarity of the inputs (**Figure S18**). The Euclidian distance for a cyclical representation of an arbitrary linear polymer (PMMA was used in the example) to a full linear graphical representation of the same polymer converges to 0 with an increasing number of repeating units. As a result, the

cyclic representation is equivalent to an infinite long linear chain. Furthermore, the computation times for the generation of the fingerprints were recorded and the cyclic representation has a significant increase in performance compared to the linear one, whose computation time increases exponentially with the number of repeating units.

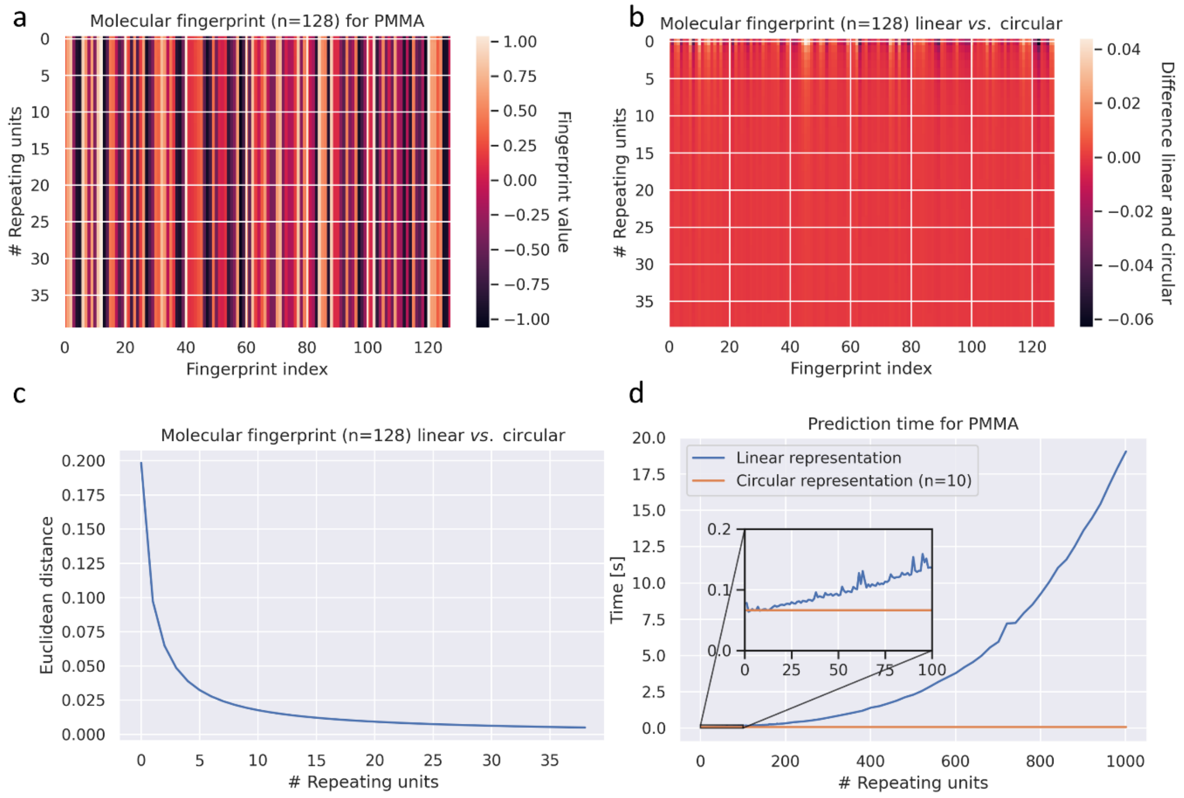

Figure S18: **a**: Random fingerprint for PMMA with different numbers of repeating units. The absolute values of the fingerprints are nearly unchanged with increasing mass. **b**: If subtracted from cyclic representation, it is clearly visible that at lower masses, the fingerprint differs from the fingerprint of the cyclic representation, but they converge with an increasing number of repeating units. **c**: The Euclidian distance of the fingerprints shows this in a clear manner. **d**: The significant benefit of the cyclic representation is the significant decrease in computation time, especially for higher numbers of repeating units.

Conversion of structural data to a node-featurized graph representation

To bring the used representing chemical structures into a data structure that can be used as input for the model, initially, simplified molecular-input line-entry system (SMILES) representations were used to create a machine-readable representation of the molecules.[4]

Therefore, diradical SMILES representations for each repeating unit were created in such a manner that a concatenation of multiple instances of the same string would result in a molecular structure in which the instances of the repeating units are connected at the radical positions. For example, the canonical SMILES representation of methyl methacrylate “C=C(C)C(=O)OC” can be written as the diradical repeating unit representation as “[CH2][C](C)C(=O)OC” which is also canonical but is not directly repeatable as a string since “[CH2][C](C)C(=O)OC[CH2][C](C)C(=O)OC“ would result in a structure, in which the next repeating unit is connected to the methyl side chain of the previous. Therefore, every repeating unit was represented in such a non-canonical manner that the backbone between the two radicals is the base string for the SMILES, and every connected atom or group of atoms has to be written in brackets to ensure a proper concatenation. For methyl methacrylate, the proper repeating unit representation would be “[CH2][C](C)**(**C(=O)OC**)**”.

To ensure a pseudo endless repeating of the units, a reasonably large number of units were concatenated. The first radical was indexed with a valid integer number, not present in the SMILES string. The same integer was appended to the complete string to create and ring closure, creating the macrocycle:

“[CH2]1[C](C)(C(=O)OC)“+”[CH2][C](C)(C(=O)OC)”*(n-1) +”1”

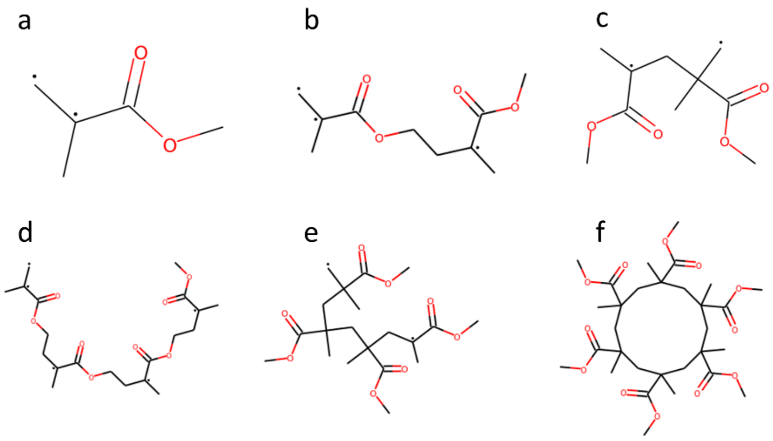

Figure S19: **a**: Structural representation from canonical SMILES *[CH2][C](C)C(=O)OC* and side-group separated SMILES *[CH2][C](C)(C(=O)OC)* **b:** Representation for two times repeated canonical SMILES *[CH2][C](C)C(=O)OC[CH2][C](C)C(=O)OC* and **c:** for side-group separated SMILES *[CH2][C](C)(C(=O)OC)[CH2][C](C)(C(=O)OC).* **d,** **e**: Extended to four times the respective SMILES *[CH2][C](C)C(=O)OC[CH2][C](C)C(=O)OC[CH2][C](C)C(=O)OC[CH2][C](C)C(=O)OC,* *[CH2][C](C)(C(=O)OC)[CH2][C](C)(C(=O)OC)[CH2][C](C)(C(=O)OC)[CH2][C](C)(C(=O)OC)* and **f**: the corresponding cyclic representation with 6 repeating units *[CH2]1[C](C)(C(=O)OC)[CH2][C](C)(C(=O)OC)[CH2][C](C)(C(=O)OC)[CH2][C](C)(C(=O)OC)[CH2][C](C)(C(=O)OC)[CH2][C](C)(C(=O)OC)1.*

Table S5: Featurization methods used to convert the molecule into a node featurized graph representation. For one-hot encoded features, the resulting vector has the same length as the set of possibilities, shown in the description, in which all elements are 0, except the one that matches the input atom, which is 1. For boolean features, a single value is generated, which is either 0 or 1, depending on whether the description matches the atom or not. For float and integer values, the result is a single value, corresponding to the value of the atom as described.

Featurizer name	Type	Length	Description
atom_symbol_hcnopsclbr_other_one_hot	one-hot encoded	9	Vector encoding of the atomic symbol in {‘H',’C',’N',’O',' P',' S',' Cl',' Br', other}
atom_degree_one_hot	one-hot encoded	8	Vector encoding of the number of connected atoms in the range [0, 7]
atom_implicit_valence_one_hot	one-hot encoded	8	Vector encoding of implicit connected hydrogen atoms in the range [0, 7]
atom_explicit_valence_one_hot	one-hot encoded	8	Vector encoding of implicit connected hydrogen atoms in the range [0, 7]
atom_formal_charge	float value	1	The formal charge of the atom
atom_partial_charge	float value	1	The partial (Gasteiger) charge of the atom
atom_hybridization_one_hot	one-hot encoded	8	Vector encoding of the atomic hybridization in{s, sp, sp^2, sp^3, sp^3d, sp^3d^2, other , unspecified }
atom_is_aromatic	boolean value	1	Whether the atom is part of an aromatic system or not
atom_total_num_H_one_hot	one-hot encoded	9	Vector encoding of the total connected hydrogen Atoms in in the range [0, 8]
atom_mass	float value	1	The mass of the atom (divided by 100 to keep the values closer to the range [0,1])
atom_num_radical_electrons	integer value	1	The number of radical electrons of the atom

Featurization of the cyclic polymer representations

The resulting macrocycle was then transformed into a rdkit.Mol object using the RDKit software.[5]

For each atom, a feature vector was created using different featurization methods (**Table S5**), and all vectors were concatenated to a feature matrix of the molecule in which the number of rows equals the number of atoms. The bonds were encoded using a $(2b) \times 2$ matrix, in which b is the number of bonds in the molecule. For the bth bond in the atom, the $2b^{\text{th}}$ row was set to the index of the source atom and of the target atom as defined in the Mol object. The $(2b + 1)^{\text{th}}$ row was set *vice versa*. This was to ensure that during graph convolution, both directions are used since the convolution works with directional graphs. Both matrices were stored in a PyTorch Geometric[6] Data object, which was used as input to the graph convolutional layers.

Simple example for featurization: For the polymer poly(methyl methacrylate) the SMILES of the repeating unit is "[CH2]1[C](C)(C(=O)OC)" and the corresponding 10-repating unit cyclic representation is generated as "[CH2]1[C](C)(C(=O)OC)[CH2][C](C)(C(=O)OC)[CH2][C](C)(C(=O)OC)[CH2][C](C)(C(=O)OC)[CH2][C](C)(C(=O)OC)[CH2][C](C)(C(=O)OC)[CH2][C](C)(C(=O)OC)[CH2][C](C)(C(=O)OC)[CH2][C](C)(C(=O)OC)[CH2][C](C)(C(=O)OC)1"

Which graphically provides:

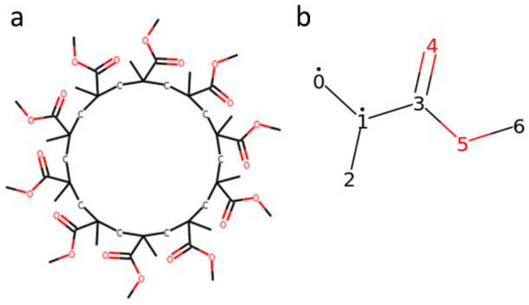

Figure S20: **a**: Structural representation of the cyclic representation of PMMA with n = 10. **b**: Enumerated structural representation of the repeating unit.

The featurization will be exemplified with the carbon of the methyl group connected to carbonyl group (**Figure S20*b*** atom 6):

atom_symbol_hcnopsclbr_other_one_hot: [0,1,0,0,0,0,0,0,0,0], *atom_degree_one_hot*: [0,1,0,0,0,0,0,0], *atom_degree_one_hot*: [0,1,0,0,0,0,0,0], *atom_implicit_valence_one_hot*: [0,0,0,1,0,0,0,0], *atom_formal_charge*: [0], *atom_partial_charge*: [0.08060857], *atom_hybridization_one_hot*: [0,0,1,0,0,0,0,0], *atom_is_aromatic*: [0], *atom_total_num_H_one_hot*: [0,0,0,1,0,0,0,0,0], *atom_mass*: [0.12011], *atom_num_radical_electrons*: [0].

And as a concatenated feature vector:

[0,1,0,0,0,0,0,0,0,0,1,0,0,0,0,0,0,0,1,0,0,0,0,0,0,0,0,1,0,0,0,0,0,0.08060857,0,0,1,0,0,0,0,0,0, 0,0,0,1,0,0,0,0,0,0.12011,0]

The featurization is repeated for every atom (including hydrogens not depicted in the structure) in the structural input, resulting in a feature vector f_a. All feature vectors are packed together to the feature matrix $H_0 \in M_{\#Atoms\times|f|}$. The feature matrix is multiplied with the symmetrically reduced adjacency matrix and the randomly initiated weight matrix. A bias is added to the resulting matrix, and the sum is passed into an activation function, resulting in a new set of features, which can be used as input for the next layer or pooled to the final output.

$$H_{n+1} = \sigma\left(\tilde{D}^{-\frac{1}{2}}\tilde{A}\tilde{D}^{-\frac{1}{2}}H_n W_n + b_n\right) \quad (2)$$

The model design. The model was implemented using PyTorch[7] as the base framework and PyTorch Geometric[6] as an additional library for graph convolution. The base model consists of an arbitrarily large number of graph convolutional layers, each with a specific feature output length (the length of the feature vector for each node after the graph convolution). Since each convolution kernel is applied to each node and its directly attached neighbors, it takes n convolutional layers to pass any feature information over n edges. For chemical input, this means the awareness of atoms within a molecule among themselves can be directly influenced *via* the number of convolutional layers. The final nanoparticle size prediction model has six layers since this should be sufficient to have a good awareness of the whole pseudo-polymeric input structure. After convolution, pooling was performed over the resulting new feature vectors, meaning a single representation value was selected based on all feature vectors for each index. For our model, we chose a combination of max-pooling and weighted sum-pooling. For weight-pooling, the resulting vector has the same shape as each feature vector, and each element is the highest element of all feature vectors at the respective index. For the weighted sum-pooling, the scalar weight of each feature vector is calculated *via* a trainable perception. The weight is then multiplied to the correlating feature vector, and all weighted vectors are added together. This vector-wise procedure can also be formulated as a multiplication of the weight-vector with the transposed feature-matrix.

$$pool_{max} = \begin{bmatrix} \max(f_{1,1}, f_{1,2}, \dots, f_{1,n}) \\ \max(f_{2,1}, f_{2,2}, \dots, f_{2,n}) \\ \vdots \\ \max(f_{m,1}, f_{m,2}, \dots, f_{m,n}) \end{bmatrix} \quad (3)$$

$$pool_{wsum} = w(F) * F^T \quad (4)$$

Where F^T is the transposed feature matrix and w is the weight vector

$$w(F) = sigmoid(a_w * F + b) \tag{5}$$

Where w is the resulting weight vector, a_w are the trainable weight vector of the perceptron, F is the feature matrix, and b is a trainable bias vector.

The resulting pooling vectors are concentrated together, resulting in a final vector with the length equal to the final feature size of the last convolutional layer times the number of pooling methods. Finally, the concatenated pooling vector is fed into a fully-connected layer with a defined output size. This output can be interpreted as a fingerprint of the structural input.

Together used with the additional input data, the absence or presence of PVA, the degree of polymerization of the polymer, and the concentration of the polymer, the fingerprint is feed into a multi-layer fully connected neuronal network. The network consists of two hidden layers with output sizes of 32 and 16. The final output layer returns a single output value, which is the predicted particle size. After each layer, a dropout of 0.1 was performed during the training of the network to prevent overfitting. A dropout larger than 0.1 results in under learning, and the training metrics are more than two times worse than the validation and never converge.

Hyperparameter optimization

The initial hyperparameter optimization was performed *via* the Optuna-Framework and the implemented default pruning algorithm.[8] For this, in each optimization step, the model was initialized with the respective hyperparameter, trained for a maximum of 200 epochs. The set of hyperparameters was optimized to result in a minimum mean absolute percentage error. An overview of the tunable hyperparameters, the range or set of each, and the optimized parameters are listed in **Table 2**.

Table 2: Hyperparameter optimized for the models used in this study.

Hyperparameter	Possible range	Optimized parameter
**Number of GC layer**	Integer [4,9]	6
**Pooling methods**	{min, mean, max, sum, weighted sum}	{max, weighted sum}
**GC-fingerprint size**	{16,32,64,128}	64
**FCNN layer reduction**	Integer [2,9]	2
**FCNN number of layers**	Integer [1,5]	2
**Dropout-rate**	{0,0.1,0.2,0.3}	0.1

Further parameters like learning rate (0.001) as well as the used featurizer are fixed parameter, to keep the space of optimizable parameter in a reasonable large size.

The complete model, including a pretrained version and customizable configuration to create new models matching new criteria or to design different model structures, can be found at https://github.com/JulianKimmig/nanoparticle_size_prediction.

Pretrained model overview

In the following section figures of the training and prediction results of a few models are presented, which can be found in the repository. All overviews consist of:

a) A graph of the evaluation parameters during training against the training epochs.
b) A plot of the predicted particle sizes against the measured one, grouped by the SMILES of the repeating unit. Here, polymers, where the model is not good at predicting the particle size, can be identified.
c) A plot of the predicted particle sizes against the measured one, grouped by the quantiles of all data, sorted by the error. Here one can see that mostly only a small percentage (high quantiles) of the data shows insufficient prediction. Furthermore, the

plot contains the test data as a separate group, including a fully removed polymer class, to evaluate the models prediction capabilities.

d) Two plots of randomly selected data points where the measured and predicted particle sizes are plotted with the error in a bar graph, once relative to the measured values and once with absolute values.
e) A plot of the testing data as a line plot, sorted by where the group with the strongest influence is highlighted in blue and the remainder in orange. The group with the strongest influence is determined by selecting all samples where the polymers are the same as the polymer in the sample with the largest error. Subsequently, the mean error of the subgroups, in which the degree of polymerization, the polymer concentration, or the additive is the same as in the sample with the largest error is determined, and the one with the highest mean error is marked as the worst-performing group.
f) The same as e but in order of appearance in the dataset.
g) The same as c but without the group identified as the worst-performing.
h) A collection of prediction grids, one for each polymer in the test- and training data. Here, particle sizes were predicted for the given polymer with a number of repeating units from 10 to 300 in concentrations from 0 to 30 g/L, with and without polyvinyl alcohol as the surfactant. The color at each point indicates the predicted particle size. Additionally, all available measurement data points in the prediction range were plotted at points, again with a color correlating to the particle size. Thus, it is easily identifiable in which regions the particle size predictions differ highly from the true data.

The model parameters for each model can be found in the respective folder in the repository under /pretrained/np_model_*X*

Pretrained model: np_model_0

In this model, poly(methyl methacrylate) (**P1-P6**) was excluded from the training data. Since PMMA was the only polymer with a degree of polymerization in the range of 1000, the model has no data to be trained on for higher degrees and thus totally overestimates the influence of higher chain lengths on the particle size. This shows nicely that for a good prediction, the model should be trained on data with broad coverage of the parameter space and that prediction is only truly reliable within the boundaries of the training data.

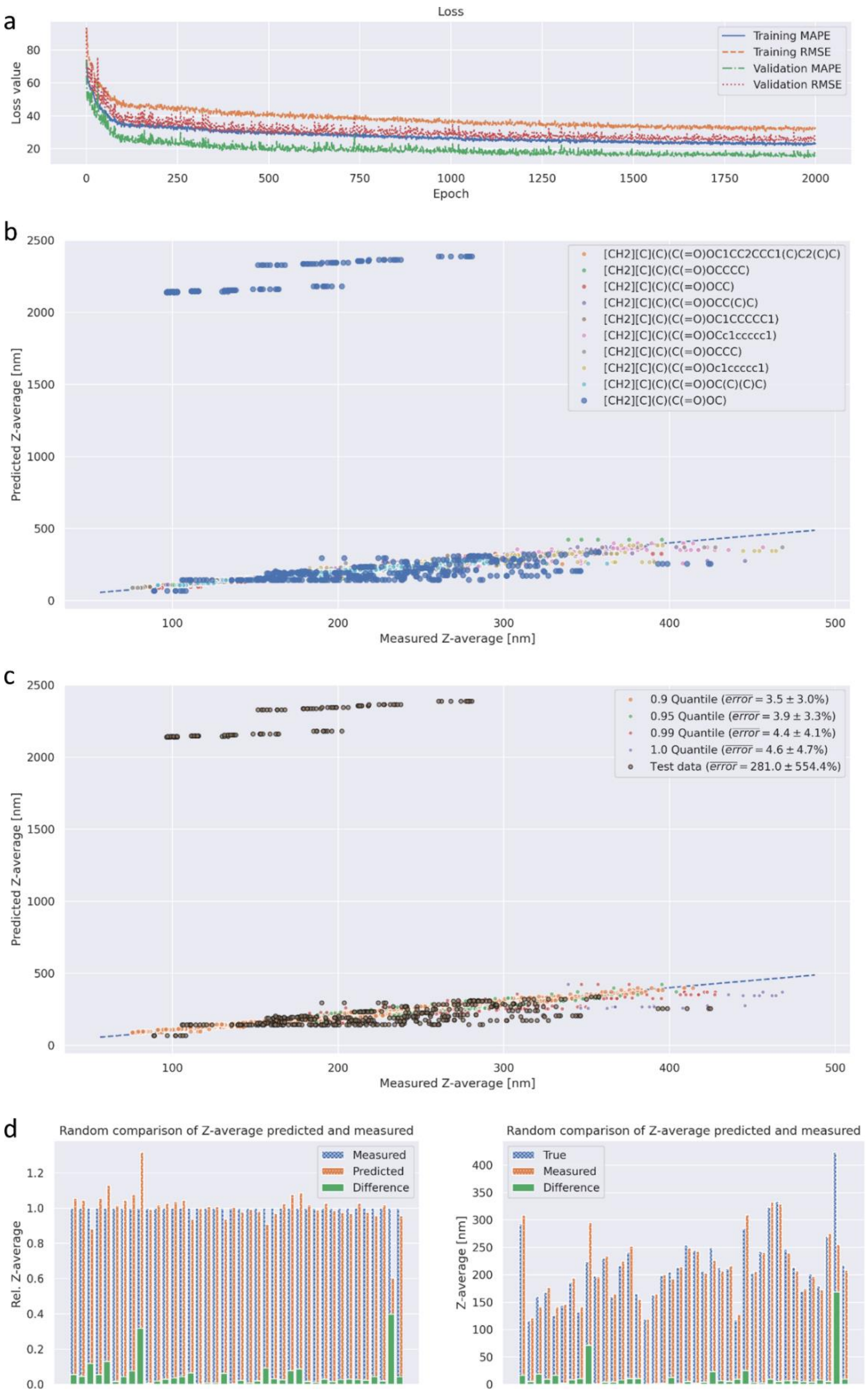
a
Loss
Training MAPE
Training RMSE
Validation MAPE
Validation RMSE
Loss value
Epoch
b
[CH2][C](C)(C(=O)OC1CC2CCC1(C)C2(C)C)
[CH2][C](C)(C(=O)OCCCC)
[CH2][C](C)(C(=O)OCC)
[CH2][C](C)(C(=O)OCC(C)C)
[CH2][C](C)(C(=O)OC1CCCCC1)
[CH2][C](C)(C(=O)OCc1ccccc1)
[CH2][C](C)(C(=O)OCCC)
[CH2][C](C)(C(=O)Oc1ccccc1)
[CH2][C](C)(C(=O)OC(C)(C)C)
[CH2][C](C)(C(=O)OC)
Predicted Z-average [nm]
Measured Z-average [nm]
c
0.9 Quantile ($\overline{error}$ = 3.5 ± 3.0%)
0.95 Quantile ($\overline{error}$ = 3.9 ± 3.3%)
0.99 Quantile ($\overline{error}$ = 4.4 ± 4.1%)
1.0 Quantile ($\overline{error}$ = 4.6 ± 4.7%)
Test data ($\overline{error}$ = 281.0 ± 554.4%)
Predicted Z-average [nm]
Measured Z-average [nm]
d
Random comparison of Z-average predicted and measured
Measured
Predicted
Difference
Rel. Z-average
Random comparison of Z-average predicted and measured
True
Measured
Difference
Z-average [nm]

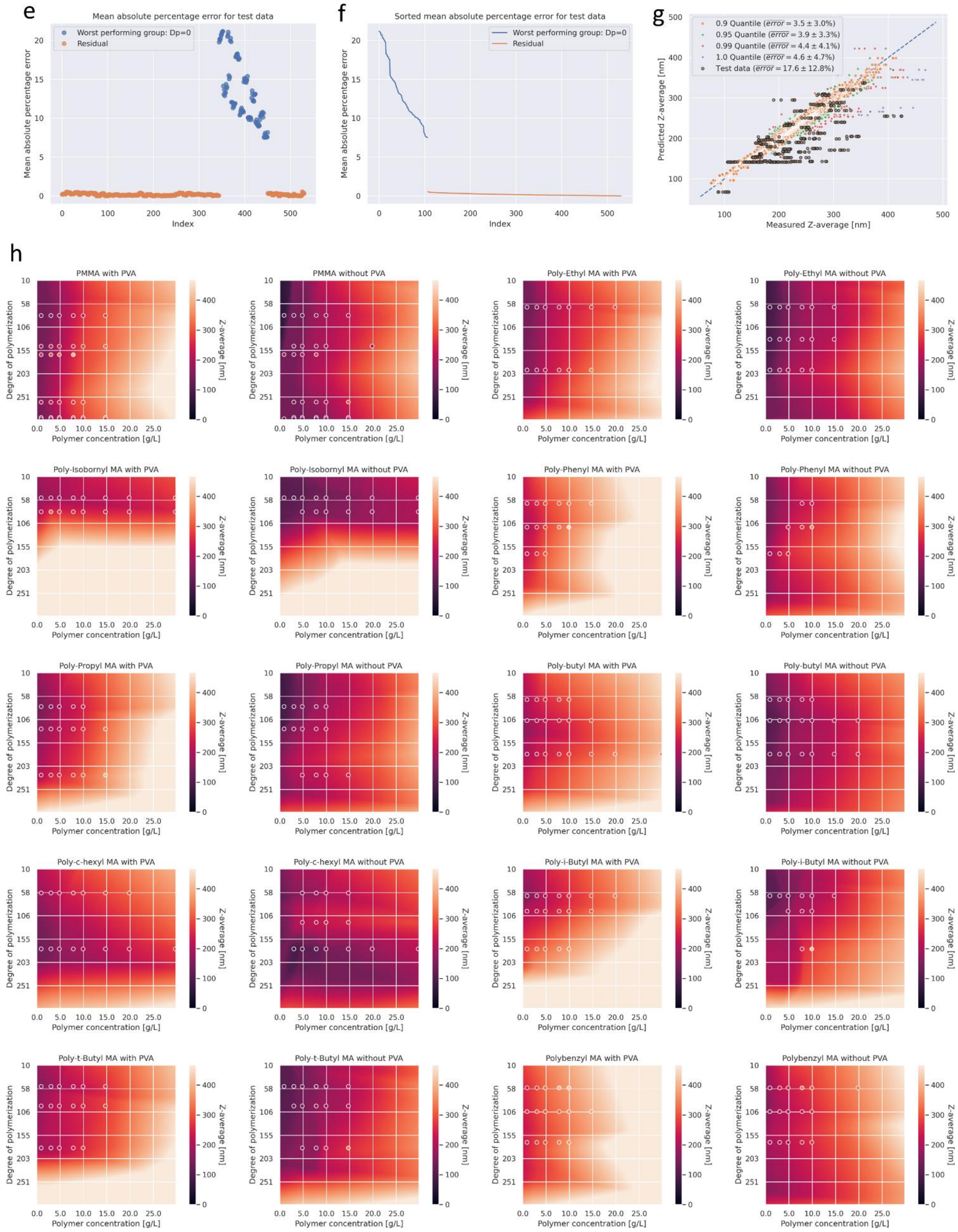

e
Mean absolute percentage error for test data
Worst performing group: Dp=0
Residual
Mean absolute percentage error
Index
f
Sorted mean absolute percentage error for test data
Worst performing group: Dp=0
Residual
Mean absolute percentage error
Index
g
0.9 Quantile ($\overline{error}$ = 3.5 ± 3.0%)
0.95 Quantile ($\overline{error}$ = 3.9 ± 3.3%)
0.99 Quantile ($\overline{error}$ = 4.4 ± 4.1%)
1.0 Quantile ($\overline{error}$ = 4.6 ± 4.7%)
Test data ($\overline{error}$ = 17.6 ± 12.8%)
Predicted Z-average [nm]
Measured Z-average [nm]
h
PMMA with PVA
PMMA without PVA
Poly-Ethyl MA with PVA
Poly-Ethyl MA without PVA
Poly-Isobornyl MA with PVA
Poly-Isobornyl MA without PVA
Poly-Phenyl MA with PVA
Poly-Phenyl MA without PVA
Poly-Propyl MA with PVA
Poly-Propyl MA without PVA
Poly-butyl MA with PVA
Poly-butyl MA without PVA
Poly-c-hexyl MA with PVA
Poly-c-hexyl MA without PVA
Poly-i-Butyl MA with PVA
Poly-i-Butyl MA without PVA
Poly-t-Butyl MA with PVA
Poly-t-Butyl MA without PVA
Polybenzyl MA with PVA
Polybenzyl MA without PVA
Degree of polymerization
Polymer concentration [g/L]
Z-average [nm]

Pretrained model: np_model_1

In this model, poly(ethyl methacrylate) (**P7-P9**) was excluded from the training data. As can be seen in e and f, the prediction error for PEMA is completely within the range of the training data.

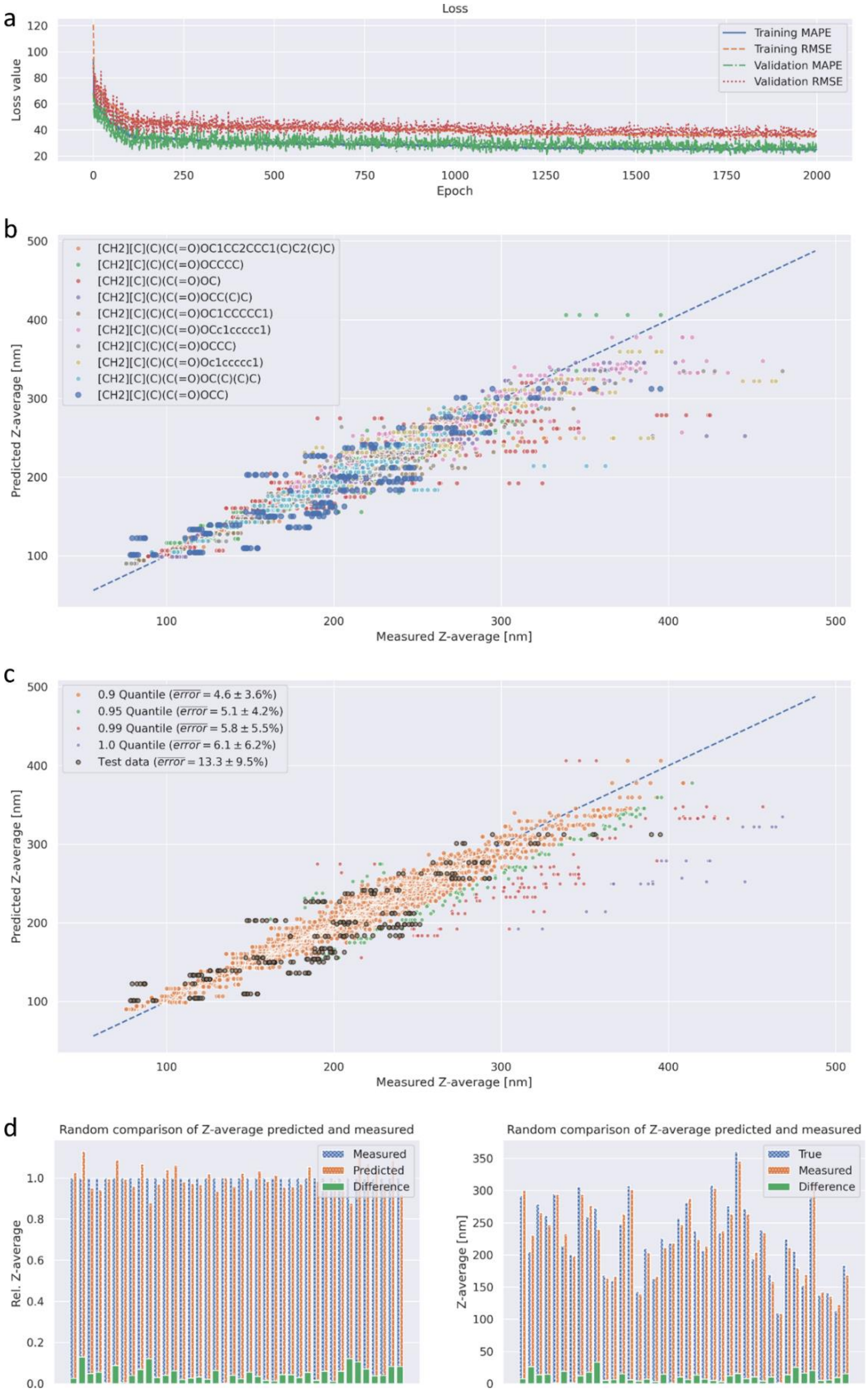

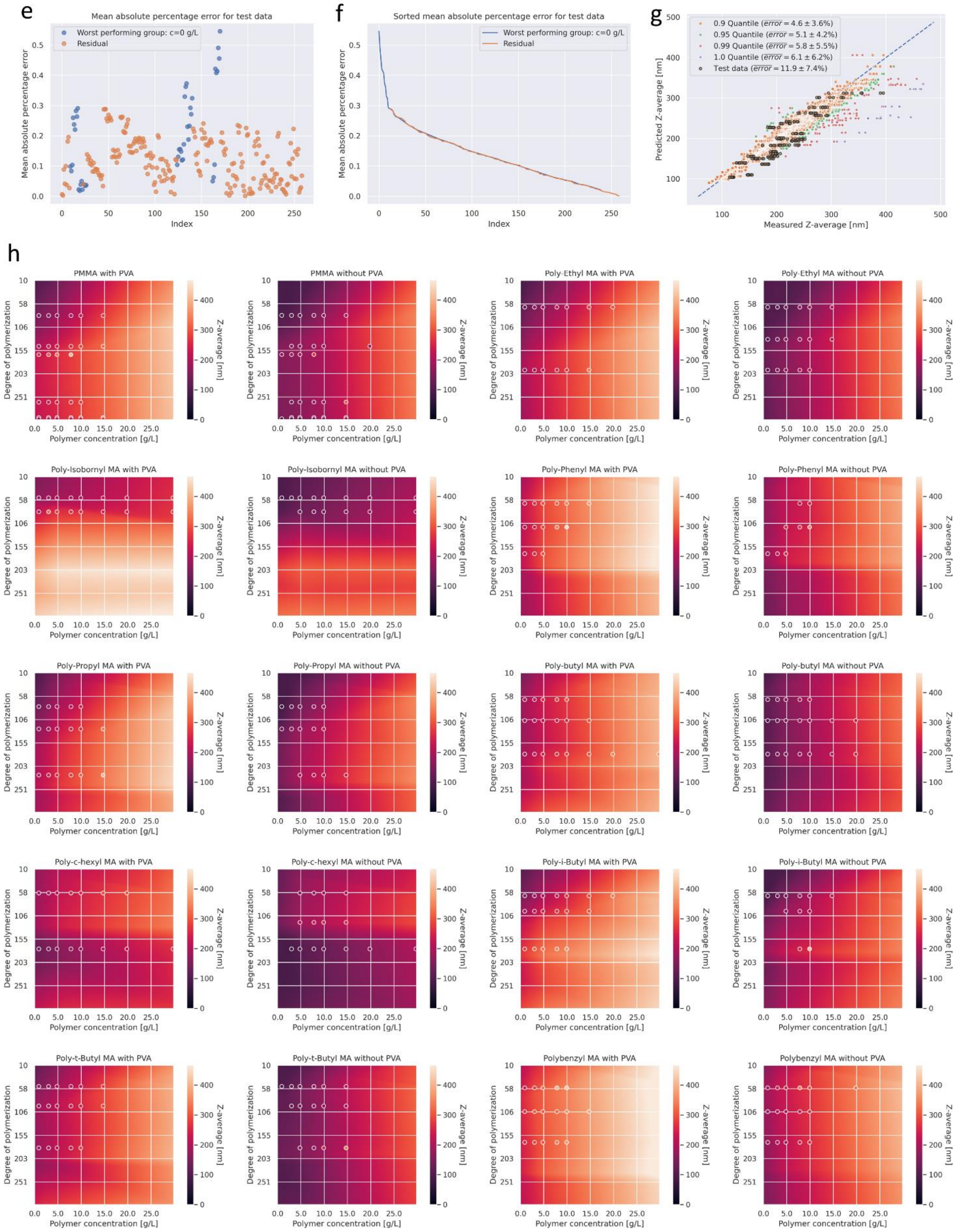

e
Mean absolute percentage error for test data
Worst performing group: c=0 g/L
Residual
Mean absolute percentage error
Index
f
Sorted mean absolute percentage error for test data
Worst performing group: c=0 g/L
Residual
Mean absolute percentage error
Index
g
0.9 Quantile ($\overline{error} = 4.6 \pm 3.6\%$)
0.95 Quantile ($\overline{error} = 5.1 \pm 4.2\%$)
0.99 Quantile ($\overline{error} = 5.8 \pm 5.5\%$)
1.0 Quantile ($\overline{error} = 6.1 \pm 6.2\%$)
Test data ($\overline{error} = 11.9 \pm 7.4\%$)
Predicted Z-average [nm]
Measured Z-average [nm]
h
PMMA with PVA
PMMA without PVA
Poly-Ethyl MA with PVA
Poly-Ethyl MA without PVA
Poly-Isobornyl MA with PVA
Poly-Isobornyl MA without PVA
Poly-Phenyl MA with PVA
Poly-Phenyl MA without PVA
Poly-Propyl MA with PVA
Poly-Propyl MA without PVA
Poly-butyl MA with PVA
Poly-butyl MA without PVA
Poly-c-hexyl MA with PVA
Poly-c-hexyl MA without PVA
Poly-i-Butyl MA with PVA
Poly-i-Butyl MA without PVA
Poly-t-Butyl MA with PVA
Poly-t-Butyl MA without PVA
Polybenzyl MA with PVA
Polybenzyl MA without PVA
Degree of polymerization
Polymer concentration [g/L]
Z-average [nm]

Pretrained model: np_model_2

In this model, poly(propyl methacrylate) **(P10-P12**) was excluded from the training data. As can be seen in e and f, the prediction error for PPMA is completely within the range of the training data.

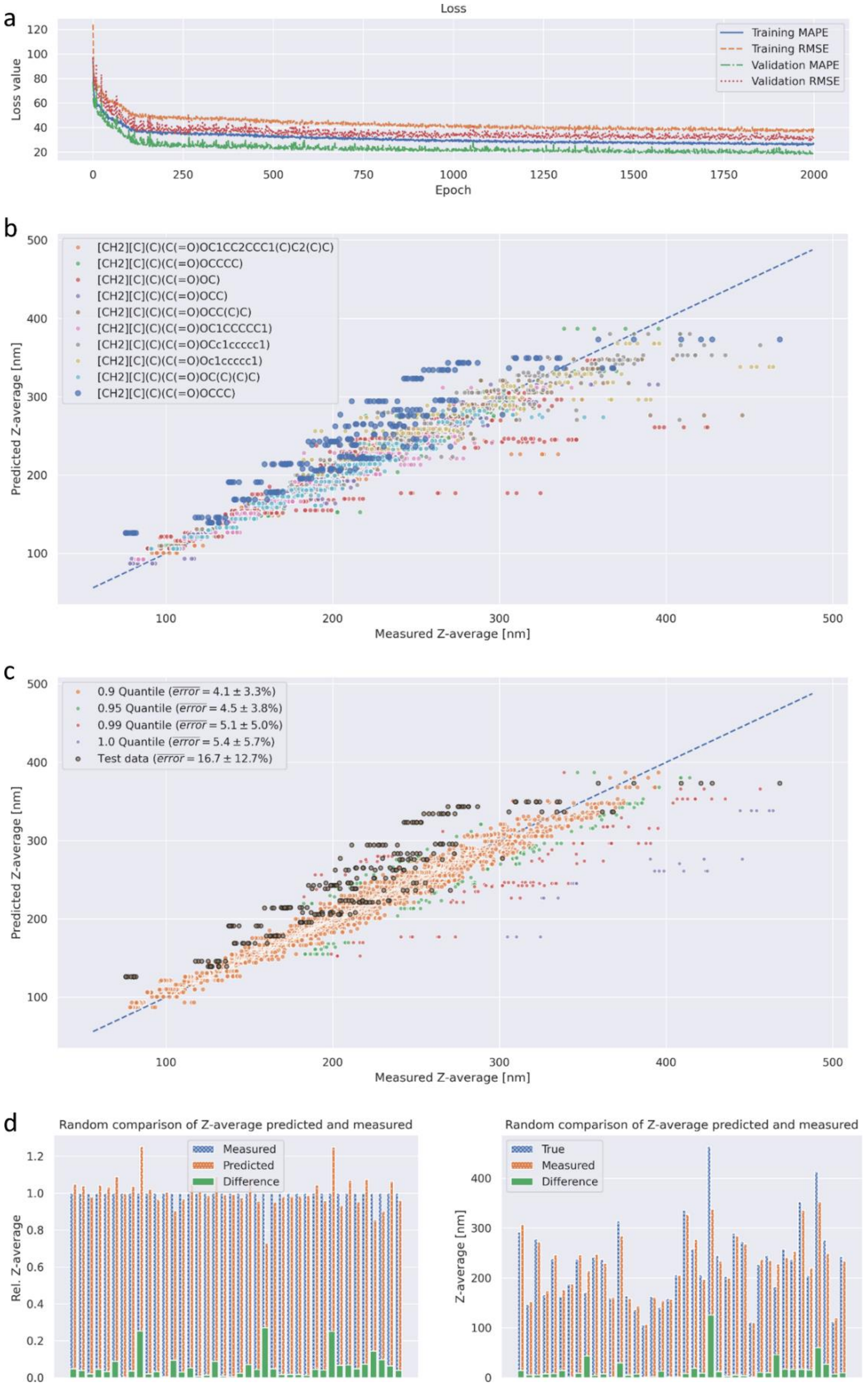

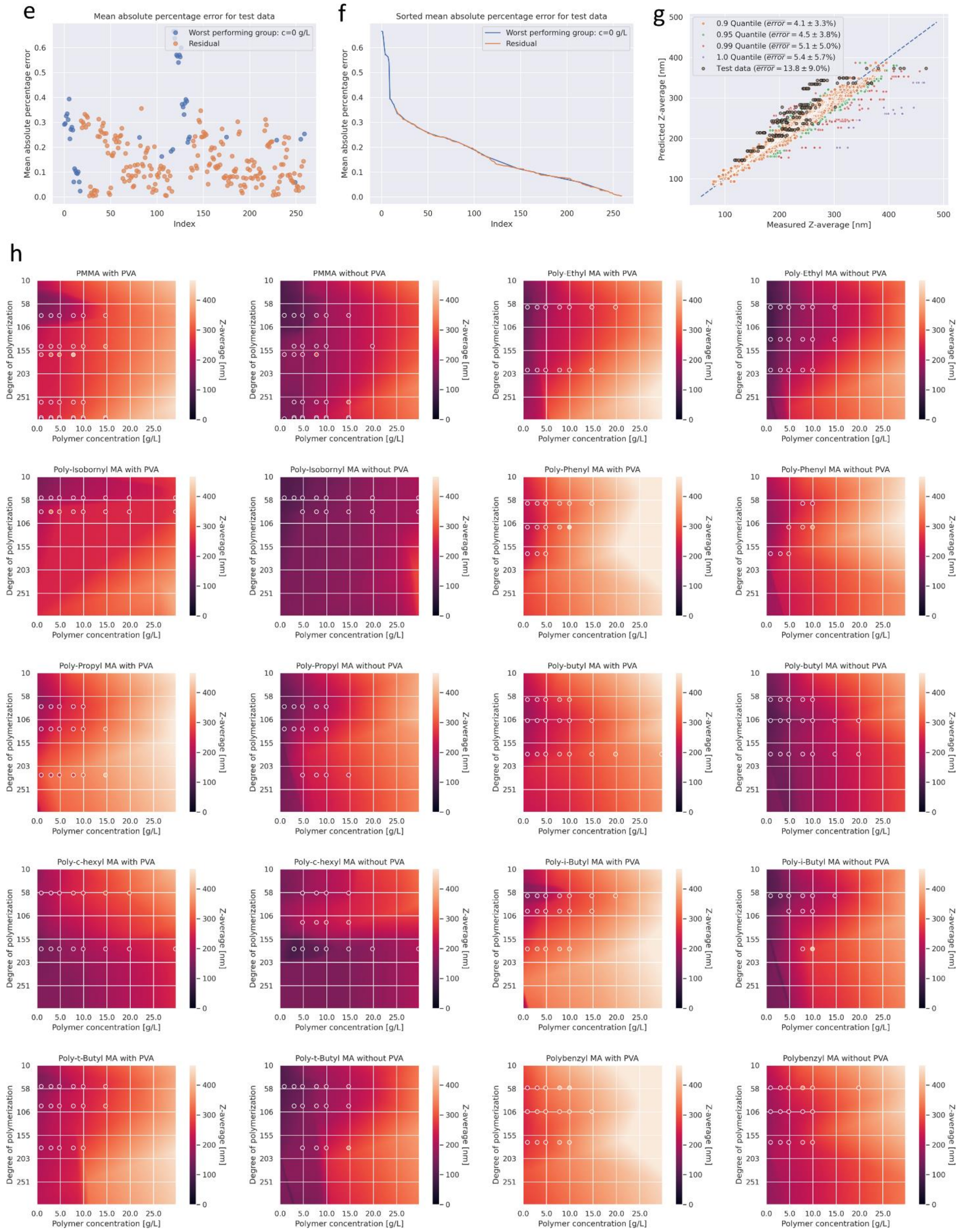
e
Mean absolute percentage error for test data
Worst performing group: c=0 g/L
Residual
Mean absolute percentage error
Index
f
Sorted mean absolute percentage error for test data
Worst performing group: c=0 g/L
Residual
Mean absolute percentage error
Index
g
0.9 Quantile ($\overline{error} = 4.1 \pm 3.3\%$)
0.95 Quantile ($\overline{error} = 4.5 \pm 3.8\%$)
0.99 Quantile ($\overline{error} = 5.1 \pm 5.0\%$)
1.0 Quantile ($\overline{error} = 5.4 \pm 5.7\%$)
Test data ($\overline{error} = 13.8 \pm 9.0\%$)
Predicted Z-average [nm]
Measured Z-average [nm]
h
PMMA with PVA
PMMA without PVA
Poly-Ethyl MA with PVA
Poly-Ethyl MA without PVA
Poly-Isobornyl MA with PVA
Poly-Isobornyl MA without PVA
Poly-Phenyl MA with PVA
Poly-Phenyl MA without PVA
Poly-Propyl MA with PVA
Poly-Propyl MA without PVA
Poly-butyl MA with PVA
Poly-butyl MA without PVA
Poly-c-hexyl MA with PVA
Poly-c-hexyl MA without PVA
Poly-i-Butyl MA with PVA
Poly-i-Butyl MA without PVA
Poly-t-Butyl MA with PVA
Poly-t-Butyl MA without PVA
Polybenzyl MA with PVA
Polybenzyl MA without PVA
Degree of polymerization
Polymer concentration [g/L]
Z-average [nm]

Pretrained model: np_model_3

In this model, poly(butyl methacrylate) (**P13-P15**) was excluded from the training data. As can be seen in e and f, the prediction error for PBMA is completely within the range of the training data. The separation in e is due to the measurement order; however, the groups in f show no separation.

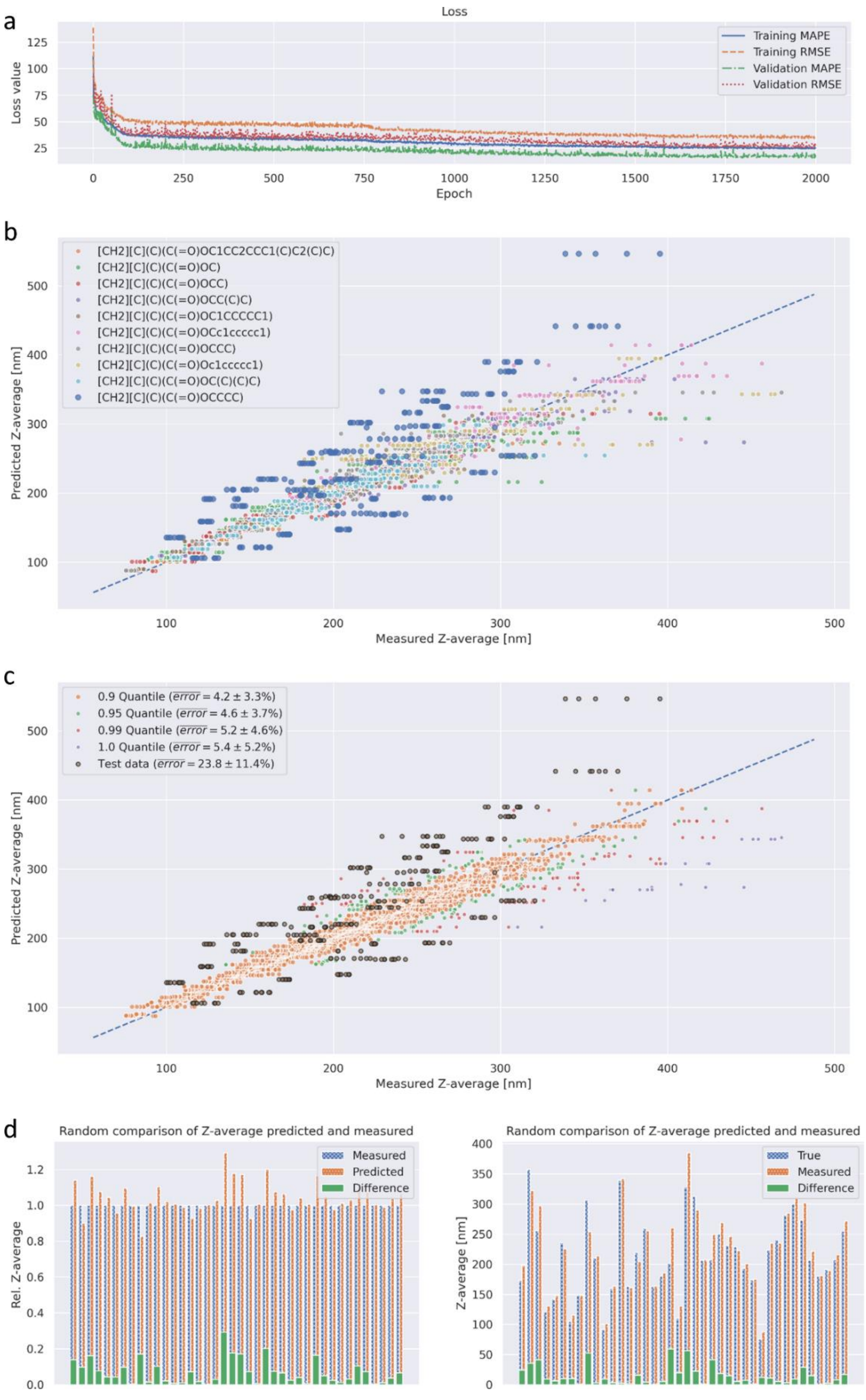
a
Loss
Training MAPE
Training RMSE
Validation MAPE
Validation RMSE
Loss value
Epoch
b
[CH2][C](C)(C(=O)OC1CC2CCC1(C)C2(C)C)
[CH2][C](C)(C(=O)OC)
[CH2][C](C)(C(=O)OCC)
[CH2][C](C)(C(=O)OCC(C)C)
[CH2][C](C)(C(=O)OC1CCCCC1)
[CH2][C](C)(C(=O)OCc1ccccc1)
[CH2][C](C)(C(=O)OCCC)
[CH2][C](C)(C(=O)Oc1ccccc1)
[CH2][C](C)(C(=O)OC(C)(C)C)
[CH2][C](C)(C(=O)OCCCC)
Predicted Z-average [nm]
Measured Z-average [nm]
c
0.9 Quantile ($\overline{error}$ = 4.2 ± 3.3%)
0.95 Quantile ($\overline{error}$ = 4.6 ± 3.7%)
0.99 Quantile ($\overline{error}$ = 5.2 ± 4.6%)
1.0 Quantile ($\overline{error}$ = 5.4 ± 5.2%)
Test data ($\overline{error}$ = 23.8 ± 11.4%)
Predicted Z-average [nm]
Measured Z-average [nm]
d
Random comparison of Z-average predicted and measured
Measured
Predicted
Difference
Rel. Z-average
Random comparison of Z-average predicted and measured
True
Measured
Difference
Z-average [nm]

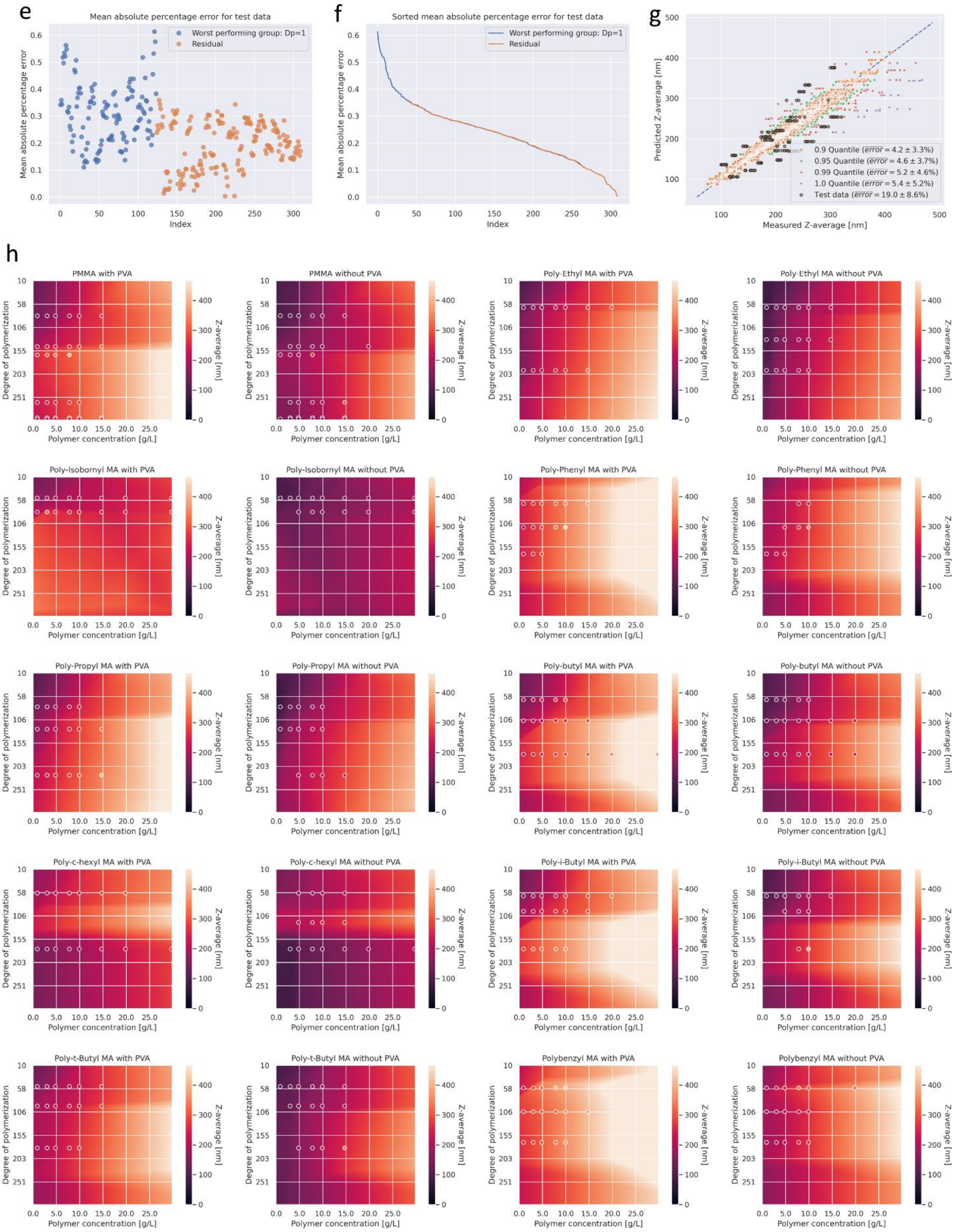
e
Mean absolute percentage error for test data
Worst performing group: Dp=1
Residual
Mean absolute percentage error
Index
f
Sorted mean absolute percentage error for test data
Worst performing group: Dp=1
Residual
Mean absolute percentage error
Index
g
Predicted Z-average [nm]
Measured Z-average [nm]
0.9 Quantile ($\overline{error}$ = 4.2 ± 3.3%)
0.95 Quantile ($\overline{error}$ = 4.6 ± 3.7%)
0.99 Quantile ($\overline{error}$ = 5.2 ± 4.6%)
1.0 Quantile ($\overline{error}$ = 5.4 ± 5.2%)
Test data ($\overline{error}$ = 19.0 ± 8.6%)
h
PMMA with PVA
PMMA without PVA
Poly-Ethyl MA with PVA
Poly-Ethyl MA without PVA
Poly-Isobornyl MA with PVA
Poly-Isobornyl MA without PVA
Poly-Phenyl MA with PVA
Poly-Phenyl MA without PVA
Poly-Propyl MA with PVA
Poly-Propyl MA without PVA
Poly-butyl MA with PVA
Poly-butyl MA without PVA
Poly-c-hexyl MA with PVA
Poly-c-hexyl MA without PVA
Poly-i-Butyl MA with PVA
Poly-i-Butyl MA without PVA
Poly-t-Butyl MA with PVA
Poly-t-Butyl MA without PVA
Polybenzyl MA with PVA
Polybenzyl MA without PVA
Degree of polymerization
Polymer concentration [g/L]
Z-average [nm]

Pretrained model: np_model_4

In this model, poly(*iso*-butyl methacrylate) (**P16-P18**) was excluded from the training data. As can be seen in e and f, the prediction error for P*i*BMA is completely within the range of the training data.

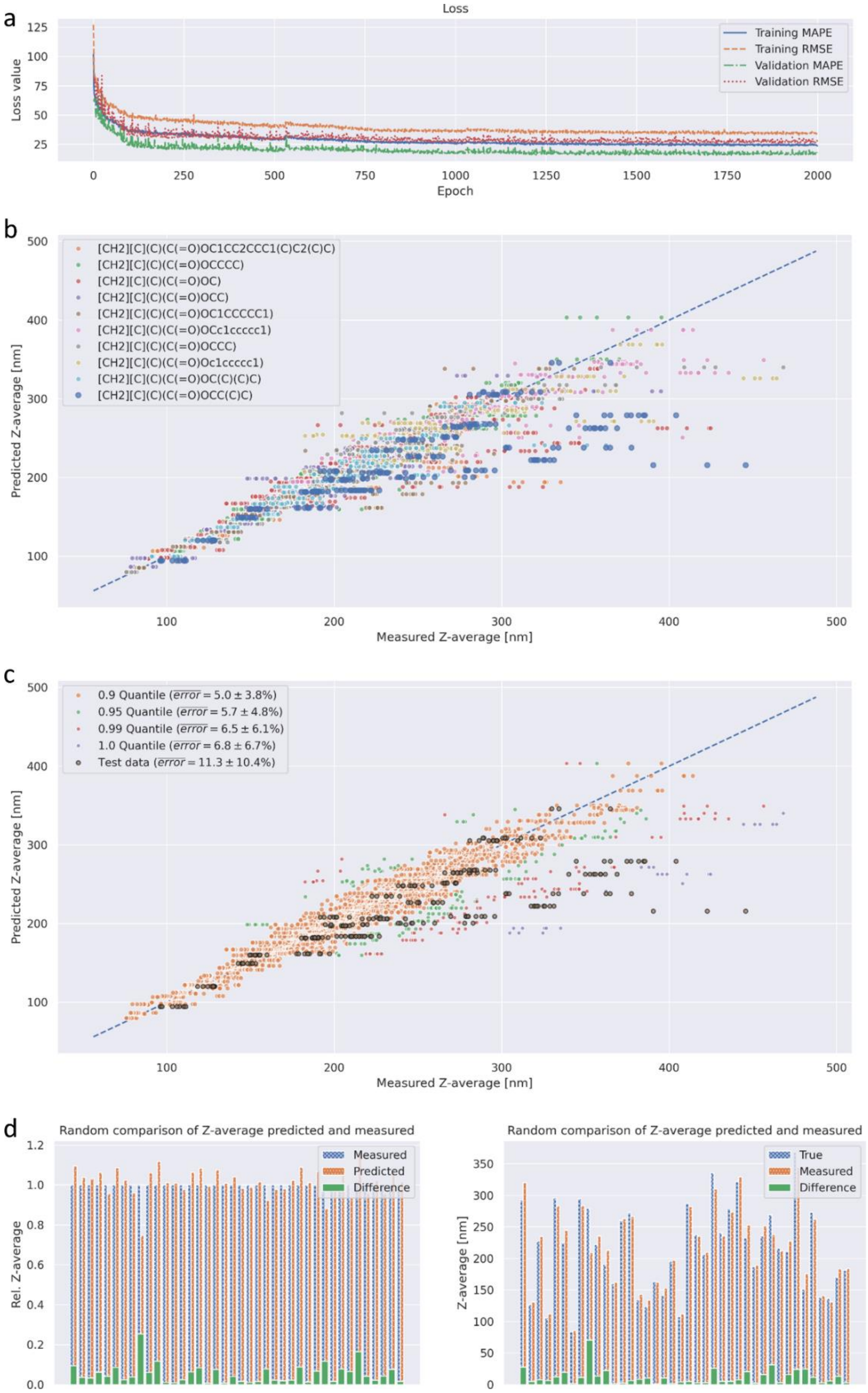

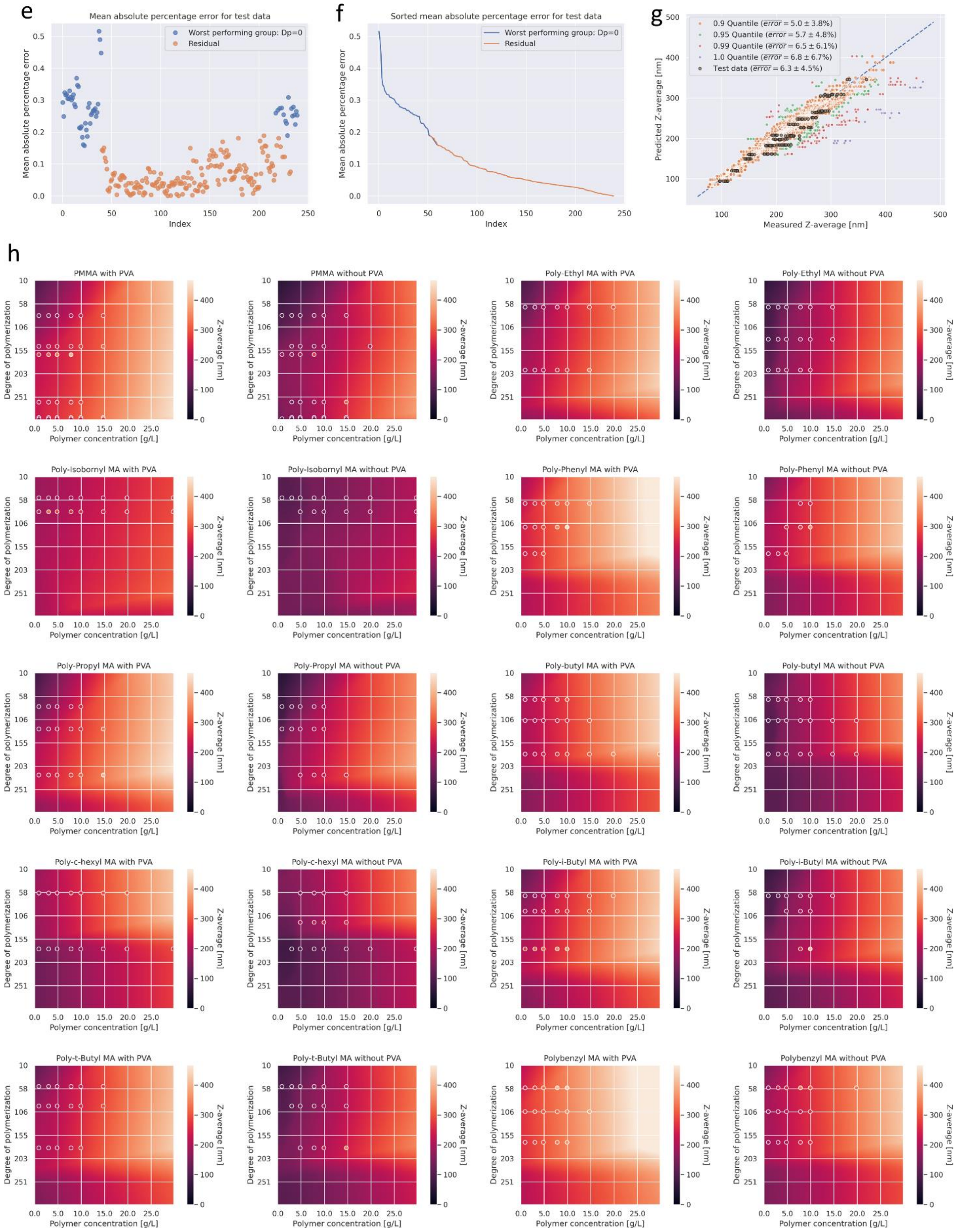
e
Mean absolute percentage error for test data
Worst performing group: Dp=0
Residual
Mean absolute percentage error
Index
f
Sorted mean absolute percentage error for test data
Worst performing group: Dp=0
Residual
Mean absolute percentage error
Index
g
0.9 Quantile ($\overline{error} = 5.0 \pm 3.8\%$)
0.95 Quantile ($\overline{error} = 5.7 \pm 4.8\%$)
0.99 Quantile ($\overline{error} = 6.5 \pm 6.1\%$)
1.0 Quantile ($\overline{error} = 6.8 \pm 6.7\%$)
Test data ($\overline{error} = 6.3 \pm 4.5\%$)
Predicted Z-average [nm]
Measured Z-average [nm]
h
PMMA with PVA
PMMA without PVA
Poly-Ethyl MA with PVA
Poly-Ethyl MA without PVA
Poly-Isobornyl MA with PVA
Poly-Isobornyl MA without PVA
Poly-Phenyl MA with PVA
Poly-Phenyl MA without PVA
Poly-Propyl MA with PVA
Poly-Propyl MA without PVA
Poly-butyl MA with PVA
Poly-butyl MA without PVA
Poly-c-hexyl MA with PVA
Poly-c-hexyl MA without PVA
Poly-i-Butyl MA with PVA
Poly-i-Butyl MA without PVA
Poly-t-Butyl MA with PVA
Poly-t-Butyl MA without PVA
Polybenzyl MA with PVA
Polybenzyl MA without PVA
Degree of polymerization
Polymer concentration [g/L]
Z-average [nm]

Pretrained model: np_model_5

In this model, poly(phenyl methacrylate) (**P28-P30**) was excluded from the training data. As can be seen in e and f, the prediction error for PPhMA is entirely within the range of the training data.

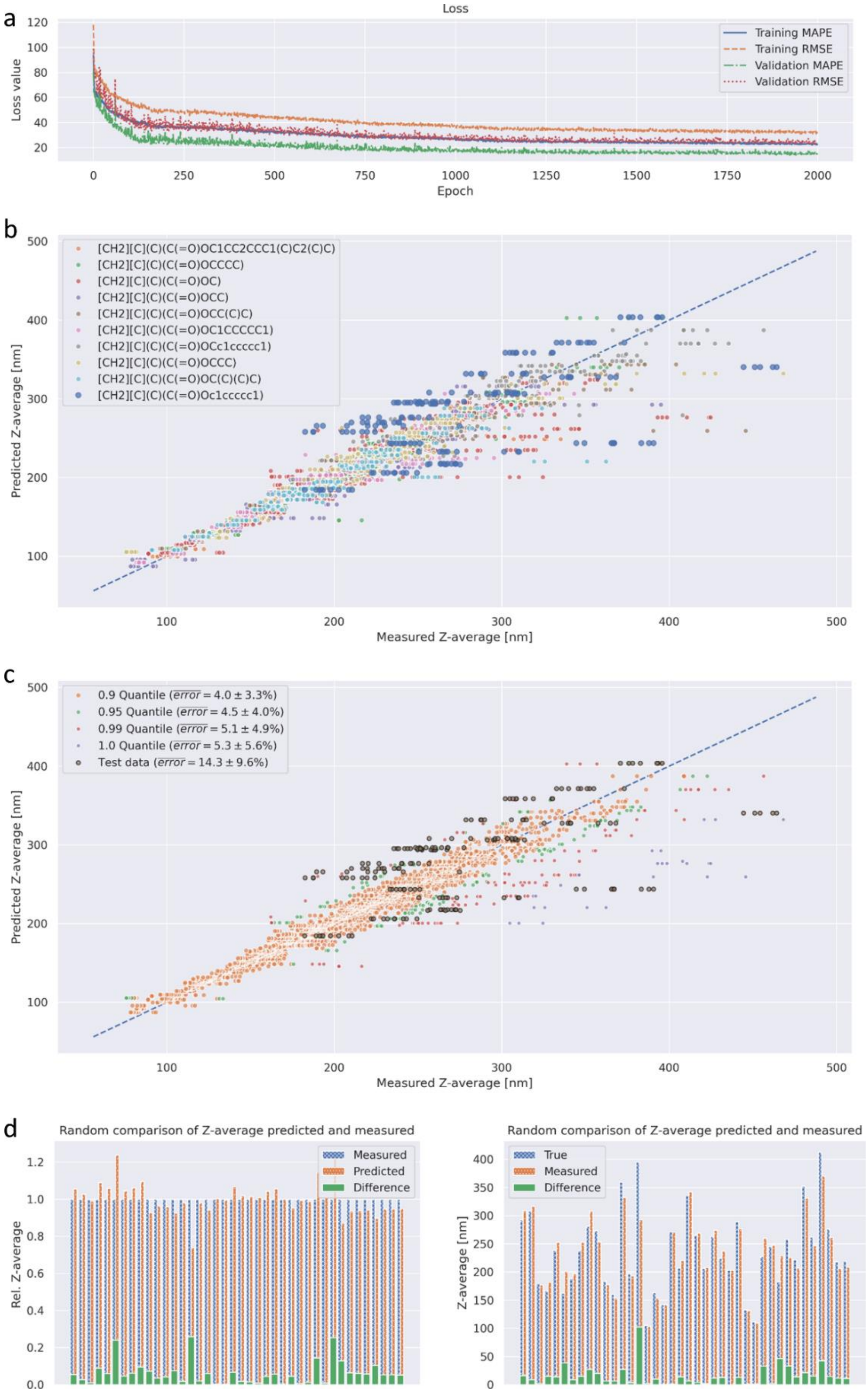

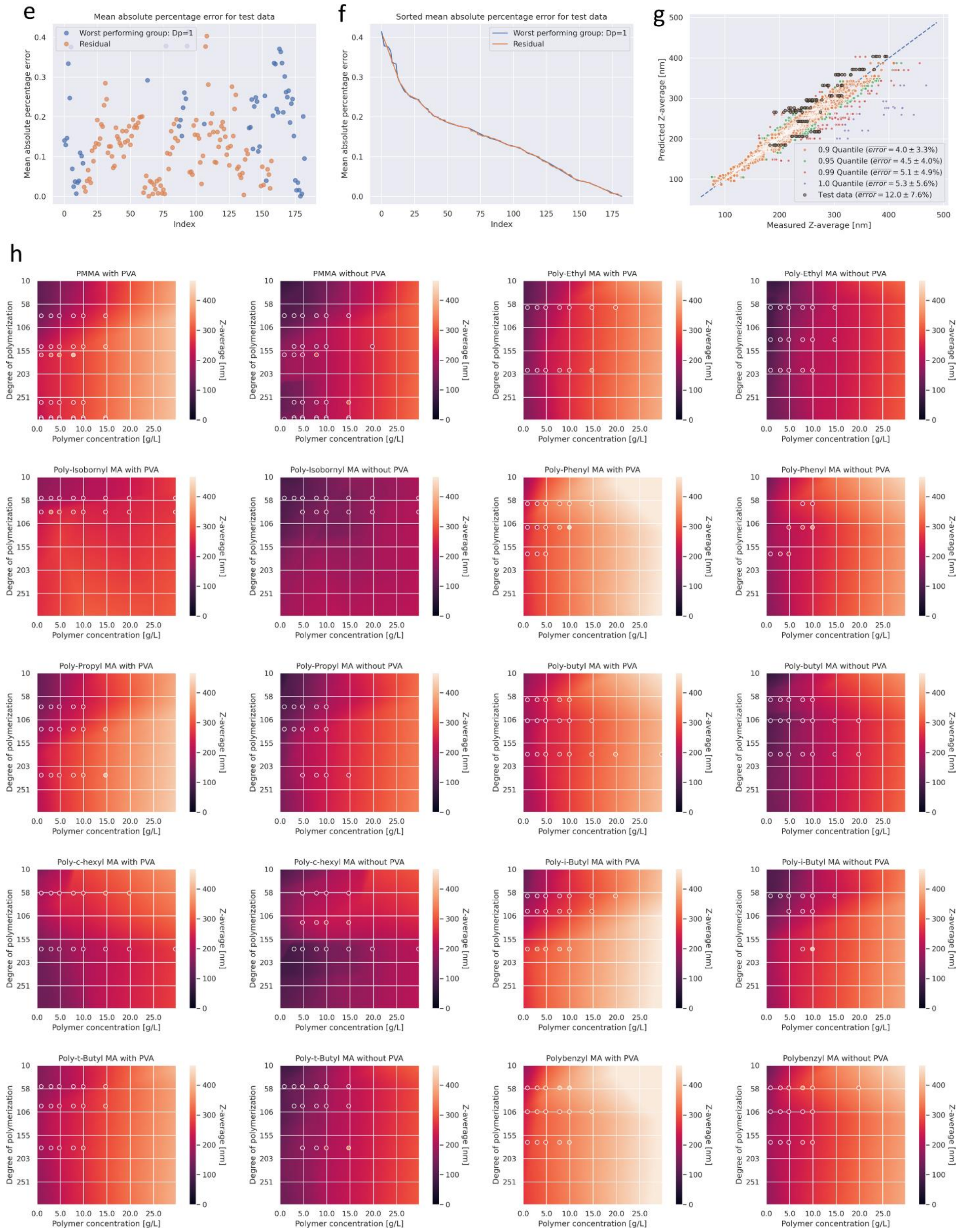
e
Mean absolute percentage error for test data
Worst performing group: Dp=1
Residual
Mean absolute percentage error
Index
f
Sorted mean absolute percentage error for test data
Worst performing group: Dp=1
Residual
Mean absolute percentage error
Index
g
Predicted Z-average [nm]
Measured Z-average [nm]
0.9 Quantile (error = 4.0 ± 3.3%)
0.95 Quantile (error = 4.5 ± 4.0%)
0.99 Quantile (error = 5.1 ± 4.9%)
1.0 Quantile (error = 5.3 ± 5.6%)
Test data (error = 12.0 ± 7.6%)
h
PMMA with PVA
PMMA without PVA
Poly-Ethyl MA with PVA
Poly-Ethyl MA without PVA
Poly-Isobornyl MA with PVA
Poly-Isobornyl MA without PVA
Poly-Phenyl MA with PVA
Poly-Phenyl MA without PVA
Poly-Propyl MA with PVA
Poly-Propyl MA without PVA
Poly-butyl MA with PVA
Poly-butyl MA without PVA
Poly-c-hexyl MA with PVA
Poly-c-hexyl MA without PVA
Poly-i-Butyl MA with PVA
Poly-i-Butyl MA without PVA
Poly-t-Butyl MA with PVA
Poly-t-Butyl MA without PVA
Polybenzyl MA with PVA
Polybenzyl MA without PVA
Degree of polymerization
Polymer concentration [g/L]
Z-average [nm]

Pretrained model: np_model_6

In this model, poly(cyclohexyl methacrylate) (**P22-P24**) was excluded from the training data. As can be seen in e and f, the prediction error for PcHexMA shows a slight separation of both groups but still with a good overlap. As a result, the prediction might still be usable but might differ from the measurement results.

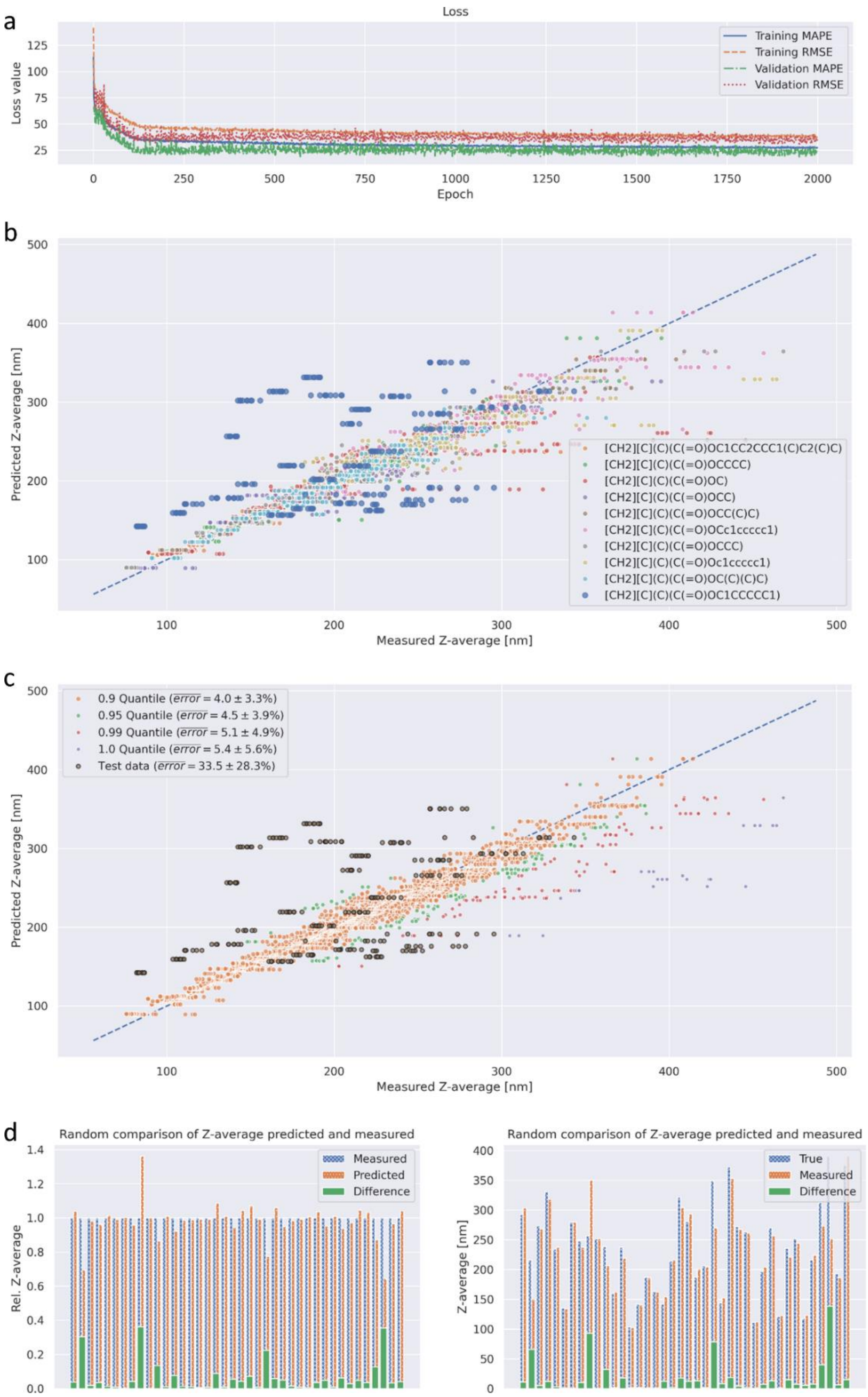
a
Loss
Training MAPE
Training RMSE
Validation MAPE
Validation RMSE
Loss value
Epoch
b
[CH2][C](C)(C(=O)OC1CC2CCC1(C)C2(C)C)
[CH2][C](C)(C(=O)OCCCC)
[CH2][C](C)(C(=O)OC)
[CH2][C](C)(C(=O)OCC)
[CH2][C](C)(C(=O)OCC(C)C)
[CH2][C](C)(C(=O)OCc1ccccc1)
[CH2][C](C)(C(=O)OCCC)
[CH2][C](C)(C(=O)Oc1ccccc1)
[CH2][C](C)(C(=O)OC(C)(C)C)
[CH2][C](C)(C(=O)OC1CCCCC1)
Predicted Z-average [nm]
Measured Z-average [nm]
c
0.9 Quantile ($\overline{error}$ = 4.0 ± 3.3%)
0.95 Quantile ($\overline{error}$ = 4.5 ± 3.9%)
0.99 Quantile ($\overline{error}$ = 5.1 ± 4.9%)
1.0 Quantile ($\overline{error}$ = 5.4 ± 5.6%)
Test data ($\overline{error}$ = 33.5 ± 28.3%)
Predicted Z-average [nm]
Measured Z-average [nm]
d
Random comparison of Z-average predicted and measured
Measured
Predicted
Difference
Rel. Z-average
Random comparison of Z-average predicted and measured
True
Measured
Difference
Z-average [nm]

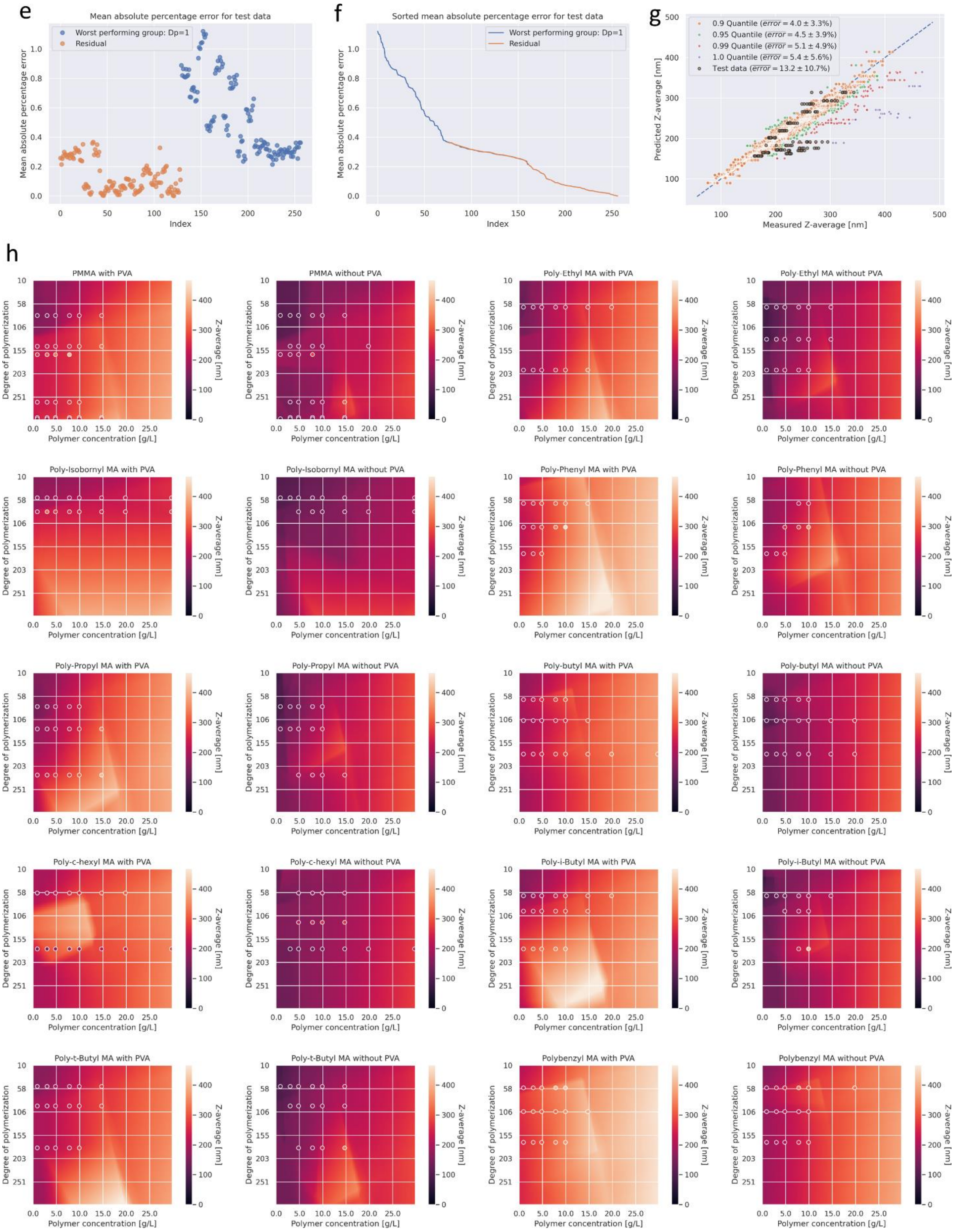
e
Mean absolute percentage error for test data
Worst performing group: Dp=1
Residual
Mean absolute percentage error
Index
f
Sorted mean absolute percentage error for test data
Worst performing group: Dp=1
Residual
Mean absolute percentage error
Index
g
0.9 Quantile ($\overline{error} = 4.0 \pm 3.3\%$)
0.95 Quantile ($\overline{error} = 4.5 \pm 3.9\%$)
0.99 Quantile ($\overline{error} = 5.1 \pm 4.9\%$)
1.0 Quantile ($\overline{error} = 5.4 \pm 5.6\%$)
Test data ($\overline{error} = 13.2 \pm 10.7\%$)
Predicted Z-average [nm]
Measured Z-average [nm]
h
PMMA with PVA
PMMA without PVA
Poly-Ethyl MA with PVA
Poly-Ethyl MA without PVA
Poly-Isobornyl MA with PVA
Poly-Isobornyl MA without PVA
Poly-Phenyl MA with PVA
Poly-Phenyl MA without PVA
Poly-Propyl MA with PVA
Poly-Propyl MA without PVA
Poly-butyl MA with PVA
Poly-butyl MA without PVA
Poly-c-hexyl MA with PVA
Poly-c-hexyl MA without PVA
Poly-i-Butyl MA with PVA
Poly-i-Butyl MA without PVA
Poly-t-Butyl MA with PVA
Poly-t-Butyl MA without PVA
Polybenzyl MA with PVA
Polybenzyl MA without PVA
Degree of polymerization
Polymer concentration [g/L]
Z-average [nm]

Pretrained model: np_model_7

In this model, poly(*tert*-butyl methacrylate) (**P19-P21**) was excluded from the training data. As can be seen in e and f, the prediction error for P*t*BMA is completely within the range of the training data.

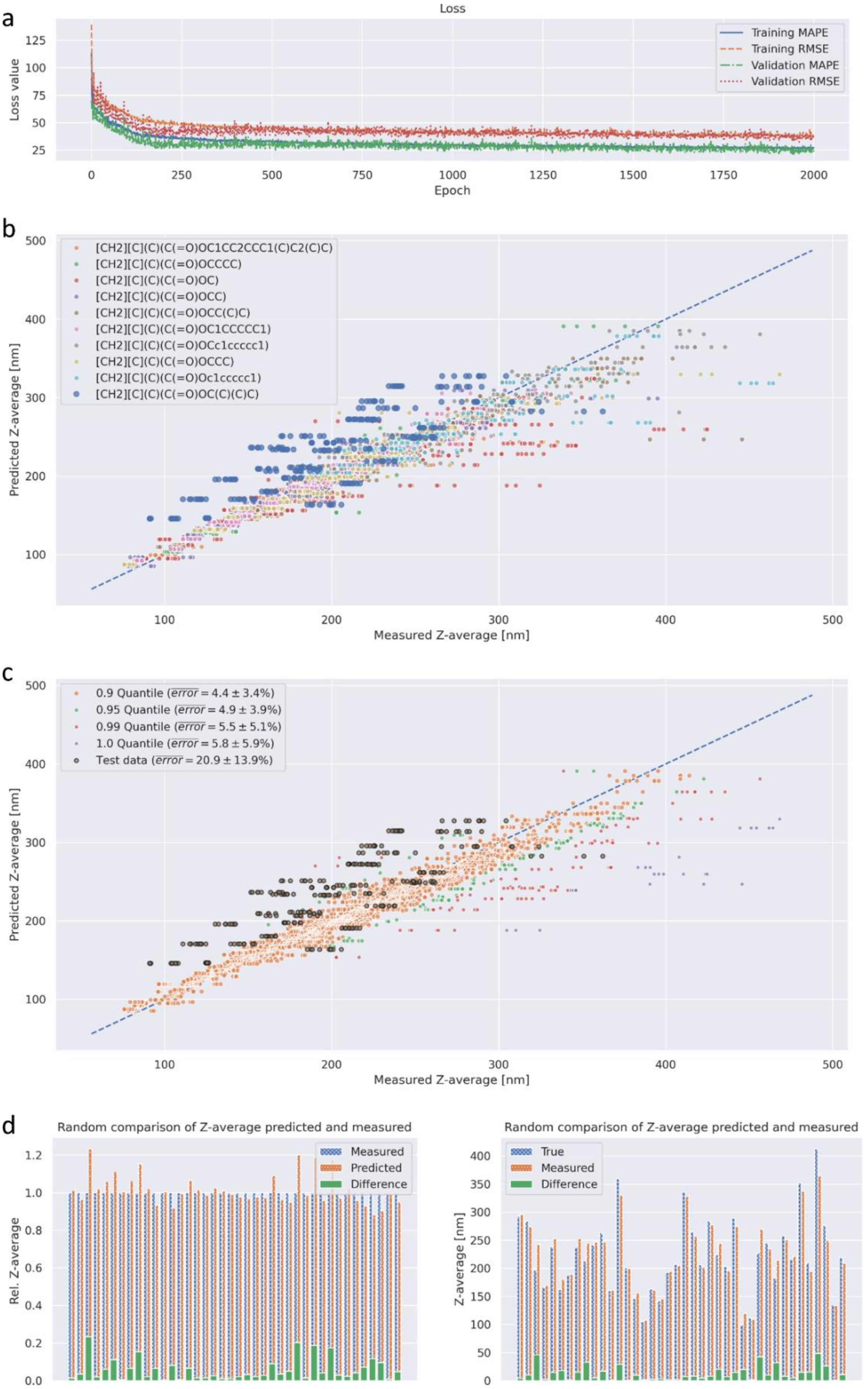

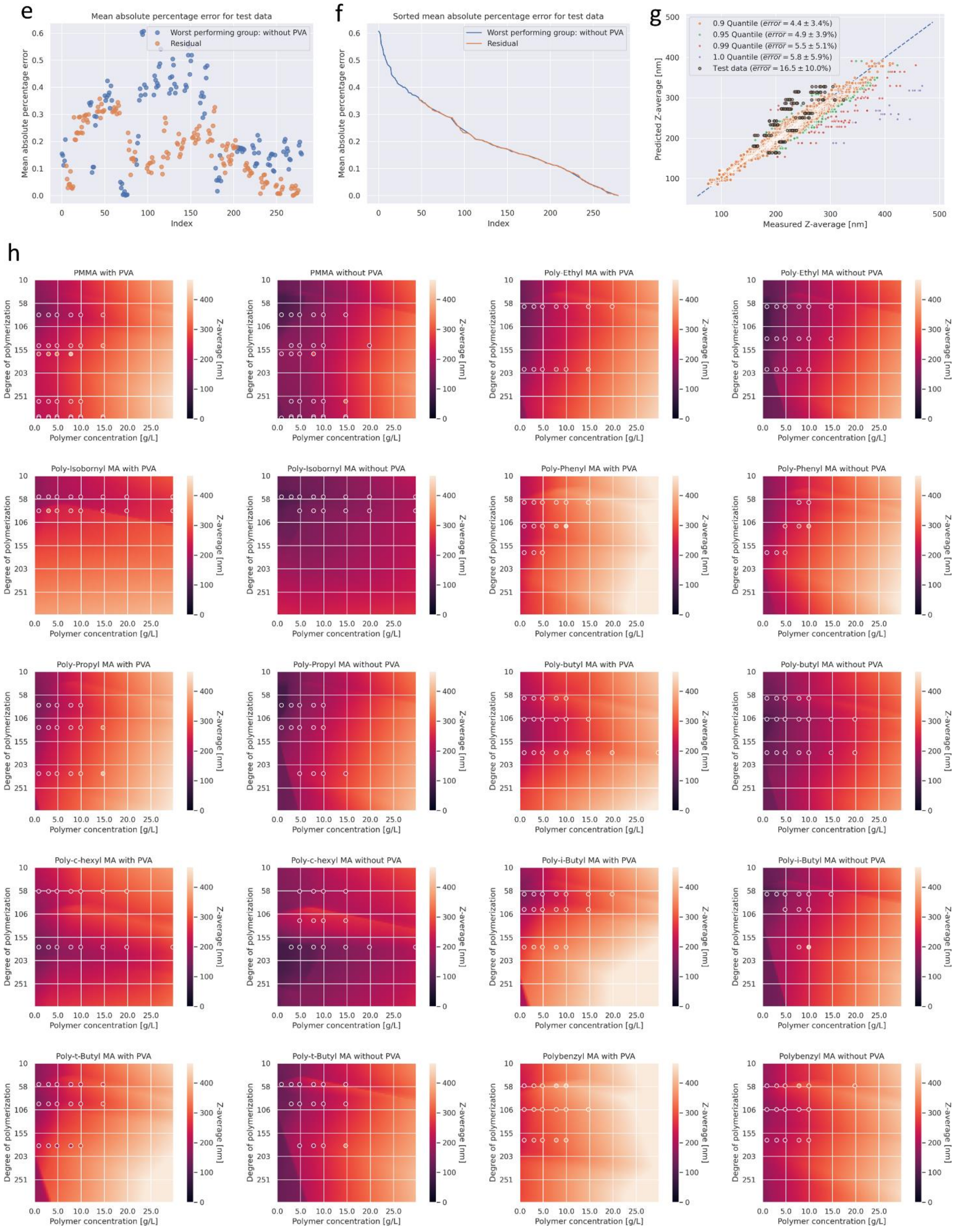
e
Mean absolute percentage error for test data
Worst performing group: without PVA
Residual
Mean absolute percentage error
Index
f
Sorted mean absolute percentage error for test data
Worst performing group: without PVA
Residual
Mean absolute percentage error
Index
g
0.9 Quantile ($\overline{error}$ = 4.4 ± 3.4%)
0.95 Quantile ($\overline{error}$ = 4.9 ± 3.9%)
0.99 Quantile ($\overline{error}$ = 5.5 ± 5.1%)
1.0 Quantile ($\overline{error}$ = 5.8 ± 5.9%)
Test data ($\overline{error}$ = 16.5 ± 10.0%)
Predicted Z-average [nm]
Measured Z-average [nm]
h
PMMA with PVA
PMMA without PVA
Poly-Ethyl MA with PVA
Poly-Ethyl MA without PVA
Poly-Isobornyl MA with PVA
Poly-Isobornyl MA without PVA
Poly-Phenyl MA with PVA
Poly-Phenyl MA without PVA
Poly-Propyl MA with PVA
Poly-Propyl MA without PVA
Poly-butyl MA with PVA
Poly-butyl MA without PVA
Poly-c-hexyl MA with PVA
Poly-c-hexyl MA without PVA
Poly-i-Butyl MA with PVA
Poly-i-Butyl MA without PVA
Poly-t-Butyl MA with PVA
Poly-t-Butyl MA without PVA
Polybenzyl MA with PVA
Polybenzyl MA without PVA
Degree of polymerization
Polymer concentration [g/L]
Z-average [nm]

Pretrained model: np_model_8

In this model, poly(benzyl methacrylate) (**P25-P27**) was excluded from the training data. As can be seen in e and f, the prediction error for PBzMA is completely within the range of the training data.

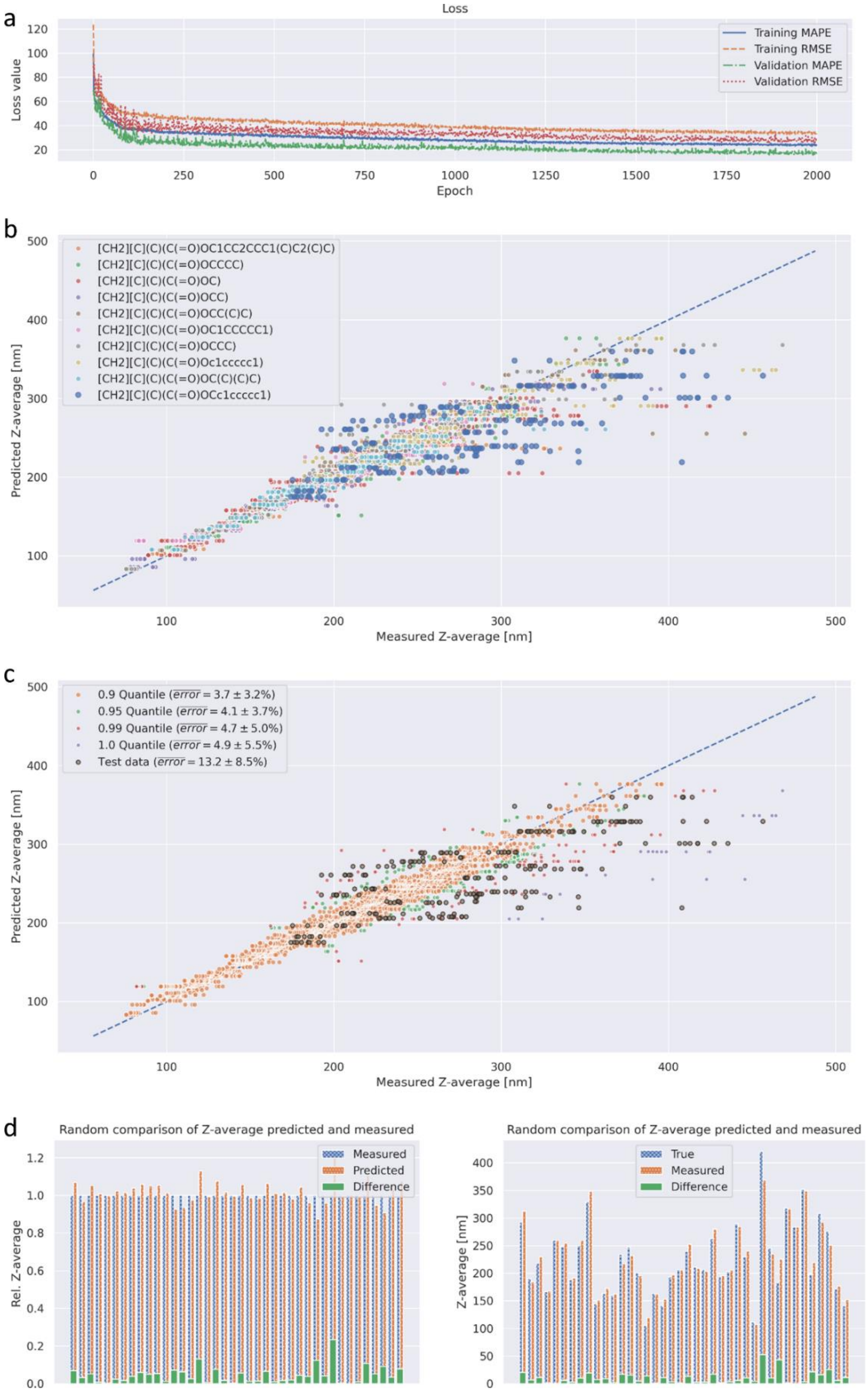

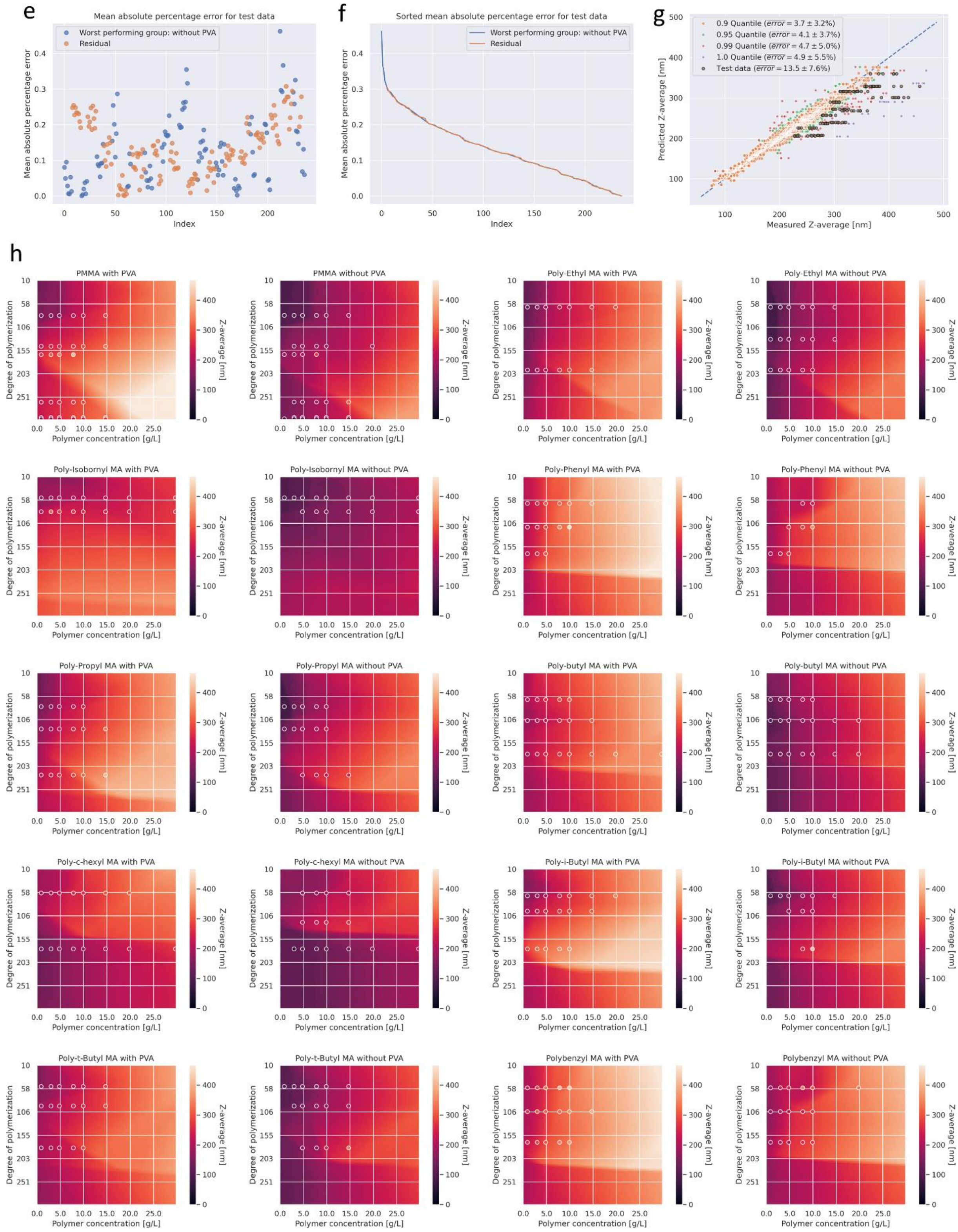
e
Mean absolute percentage error for test data
Worst performing group: without PVA
Residual
Mean absolute percentage error
Index
f
Sorted mean absolute percentage error for test data
Worst performing group: without PVA
Residual
Mean absolute percentage error
Index
g
0.9 Quantile ($\overline{error}$ = 3.7 ± 3.2%)
0.95 Quantile ($\overline{error}$ = 4.1 ± 3.7%)
0.99 Quantile ($\overline{error}$ = 4.7 ± 5.0%)
1.0 Quantile ($\overline{error}$ = 4.9 ± 5.5%)
Test data ($\overline{error}$ = 13.5 ± 7.6%)
Predicted Z-average [nm]
Measured Z-average [nm]
h
PMMA with PVA
PMMA without PVA
Poly-Ethyl MA with PVA
Poly-Ethyl MA without PVA
Poly-Isobornyl MA with PVA
Poly-Isobornyl MA without PVA
Poly-Phenyl MA with PVA
Poly-Phenyl MA without PVA
Poly-Propyl MA with PVA
Poly-Propyl MA without PVA
Poly-butyl MA with PVA
Poly-butyl MA without PVA
Poly-c-hexyl MA with PVA
Poly-c-hexyl MA without PVA
Poly-i-Butyl MA with PVA
Poly-i-Butyl MA without PVA
Poly-t-Butyl MA with PVA
Poly-t-Butyl MA without PVA
Polybenzyl MA with PVA
Polybenzyl MA without PVA
Degree of polymerization
Polymer concentration [g/L]
Z-average [nm]

Pretrained model: np_model_9

In this model, poly(isobornyl methacrylate) (**P30-P33**) was excluded from the training data. As can be seen in e and f, the prediction error cannot be separated into distinguished groups but is overall relatively high. This means the model has, in general, a problem to predict particle sizes for poly(isobornyl methacrylate) nanoparticles, probably due to the structure, which is unique within the training data. To get a better performing model, more training data with different polymer structures would be necessary.

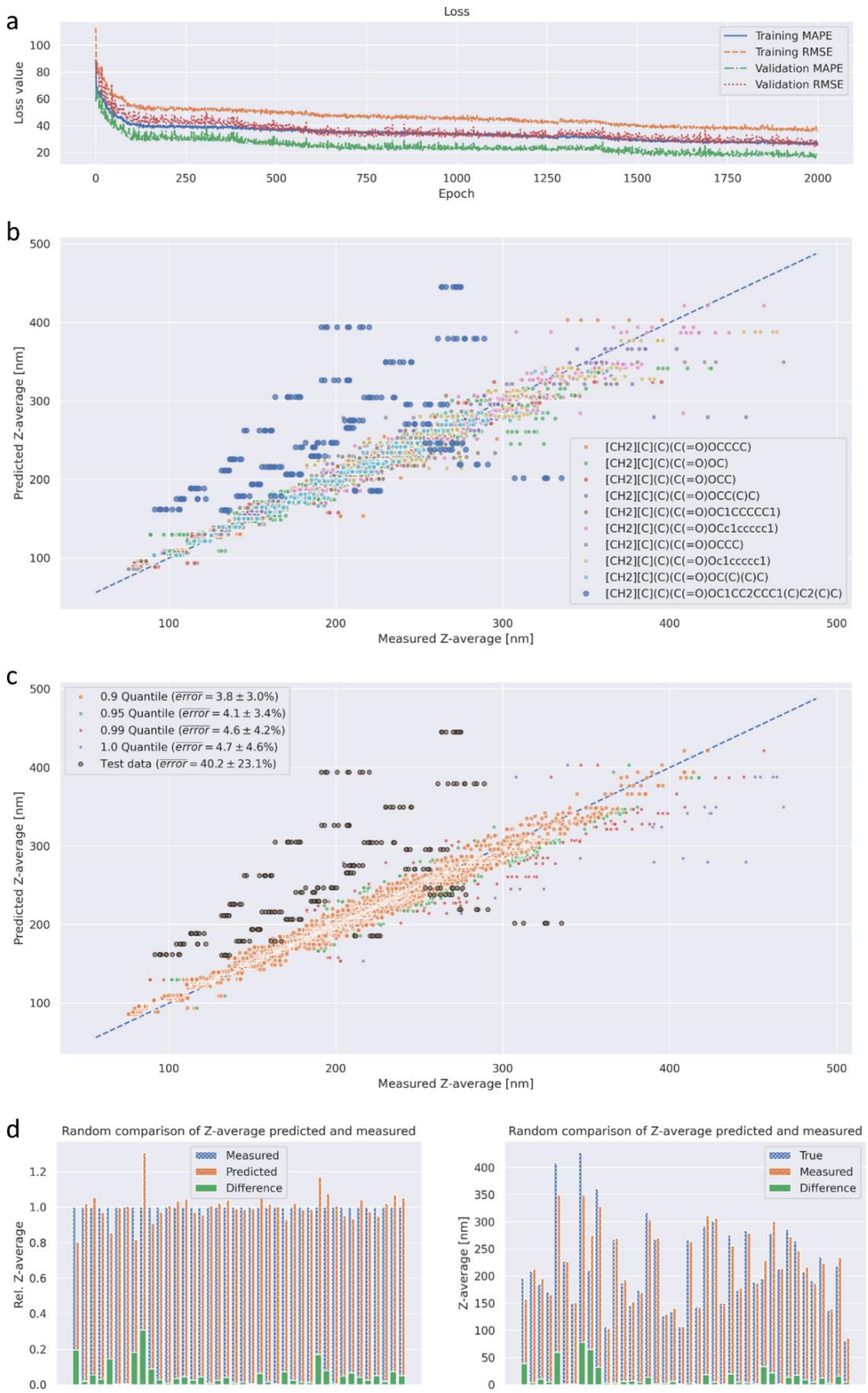
a
Loss
Loss value
Epoch
Training MAPE
Training RMSE
Validation MAPE
Validation RMSE
b
Predicted Z-average [nm]
Measured Z-average [nm]
[CH2][C](C)(C(=O)OCCCC)
[CH2][C](C)(C(=O)OC)
[CH2][C](C)(C(=O)OCC)
[CH2][C](C)(C(=O)OCC(C)C)
[CH2][C](C)(C(=O)OC1CCCCC1)
[CH2][C](C)(C(=O)OCc1ccccc1)
[CH2][C](C)(C(=O)OCCC)
[CH2][C](C)(C(=O)Oc1ccccc1)
[CH2][C](C)(C(=O)OC(C)(C)C)
[CH2][C](C)(C(=O)OC1CC2CCC1(C)C2(C)C)
c
0.9 Quantile ($\overline{error} = 3.8 \pm 3.0\%$)
0.95 Quantile ($\overline{error} = 4.1 \pm 3.4\%$)
0.99 Quantile ($\overline{error} = 4.6 \pm 4.2\%$)
1.0 Quantile ($\overline{error} = 4.7 \pm 4.6\%$)
Test data ($\overline{error} = 40.2 \pm 23.1\%$)
Predicted Z-average [nm]
Measured Z-average [nm]
d
Random comparison of Z-average predicted and measured
Rel. Z-average
Measured
Predicted
Difference
Random comparison of Z-average predicted and measured
Z-average [nm]
True
Measured
Difference

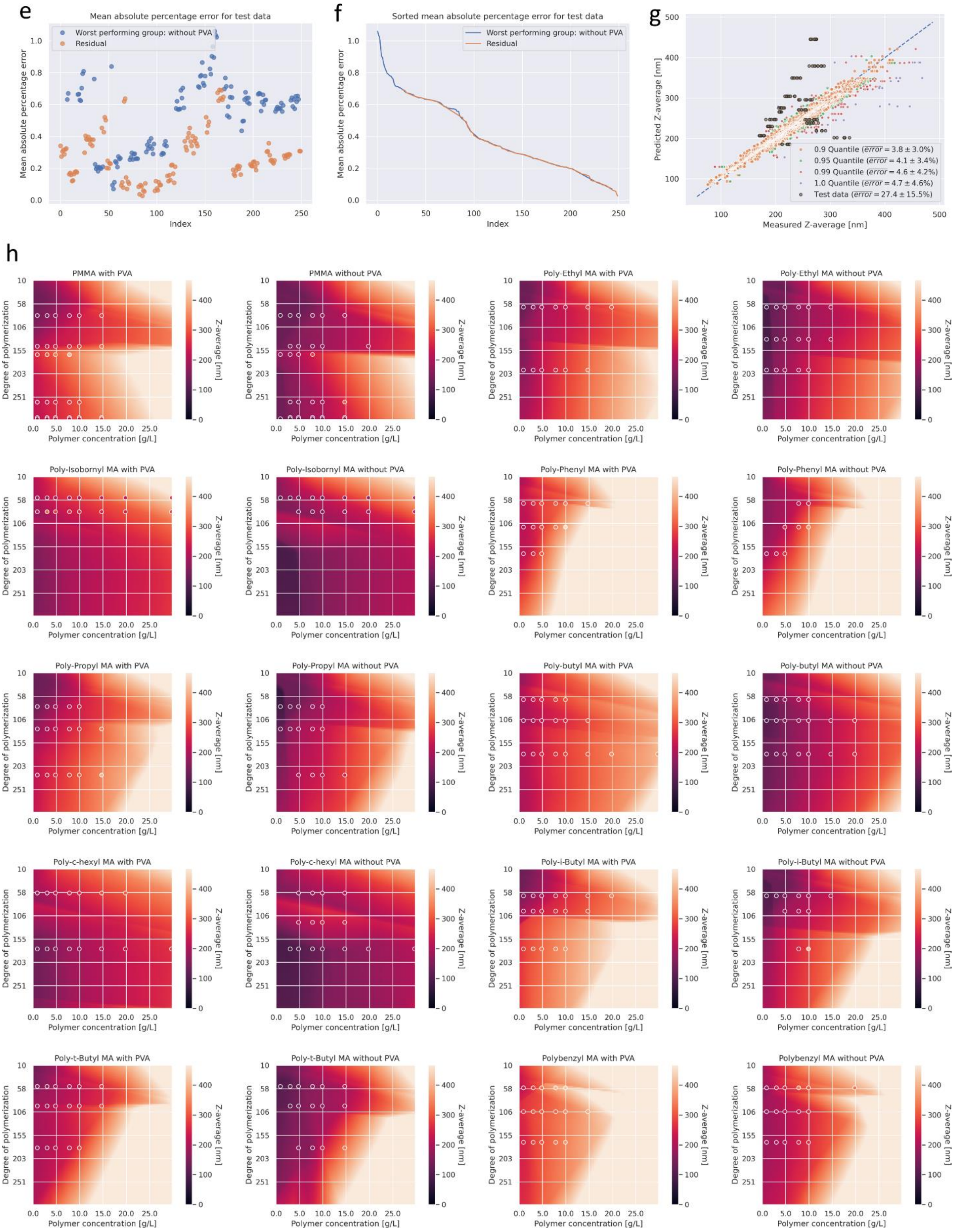
e
Mean absolute percentage error for test data
Worst performing group: without PVA
Residual
Mean absolute percentage error
Index
f
Sorted mean absolute percentage error for test data
Worst performing group: without PVA
Residual
Mean absolute percentage error
Index
g
0.9 Quantile (error = 3.8 ± 3.0%)
0.95 Quantile (error = 4.1 ± 3.4%)
0.99 Quantile (error = 4.6 ± 4.2%)
1.0 Quantile (error = 4.7 ± 4.6%)
Test data (error = 27.4 ± 15.5%)
Predicted Z-average [nm]
Measured Z-average [nm]
h
PMMA with PVA
PMMA without PVA
Poly-Ethyl MA with PVA
Poly-Ethyl MA without PVA
Poly-Isobornyl MA with PVA
Poly-Isobornyl MA without PVA
Poly-Phenyl MA with PVA
Poly-Phenyl MA without PVA
Poly-Propyl MA with PVA
Poly-Propyl MA without PVA
Poly-butyl MA with PVA
Poly-butyl MA without PVA
Poly-c-hexyl MA with PVA
Poly-c-hexyl MA without PVA
Poly-i-Butyl MA with PVA
Poly-i-Butyl MA without PVA
Poly-t-Butyl MA with PVA
Poly-t-Butyl MA without PVA
Polybenzyl MA with PVA
Polybenzyl MA without PVA
Degree of polymerization
Polymer concentration [g/L]
Z-average [nm]

Pretrained model: np_model_10

In this case the model was trained with a fully connected network with only a single hidden layer. As a result, the output is not as accurate but seems to be much smother. Ideally this smoothness should also be reachable by deeper networks with more trainable parameters, but much more datapoints are needed to achieve this.

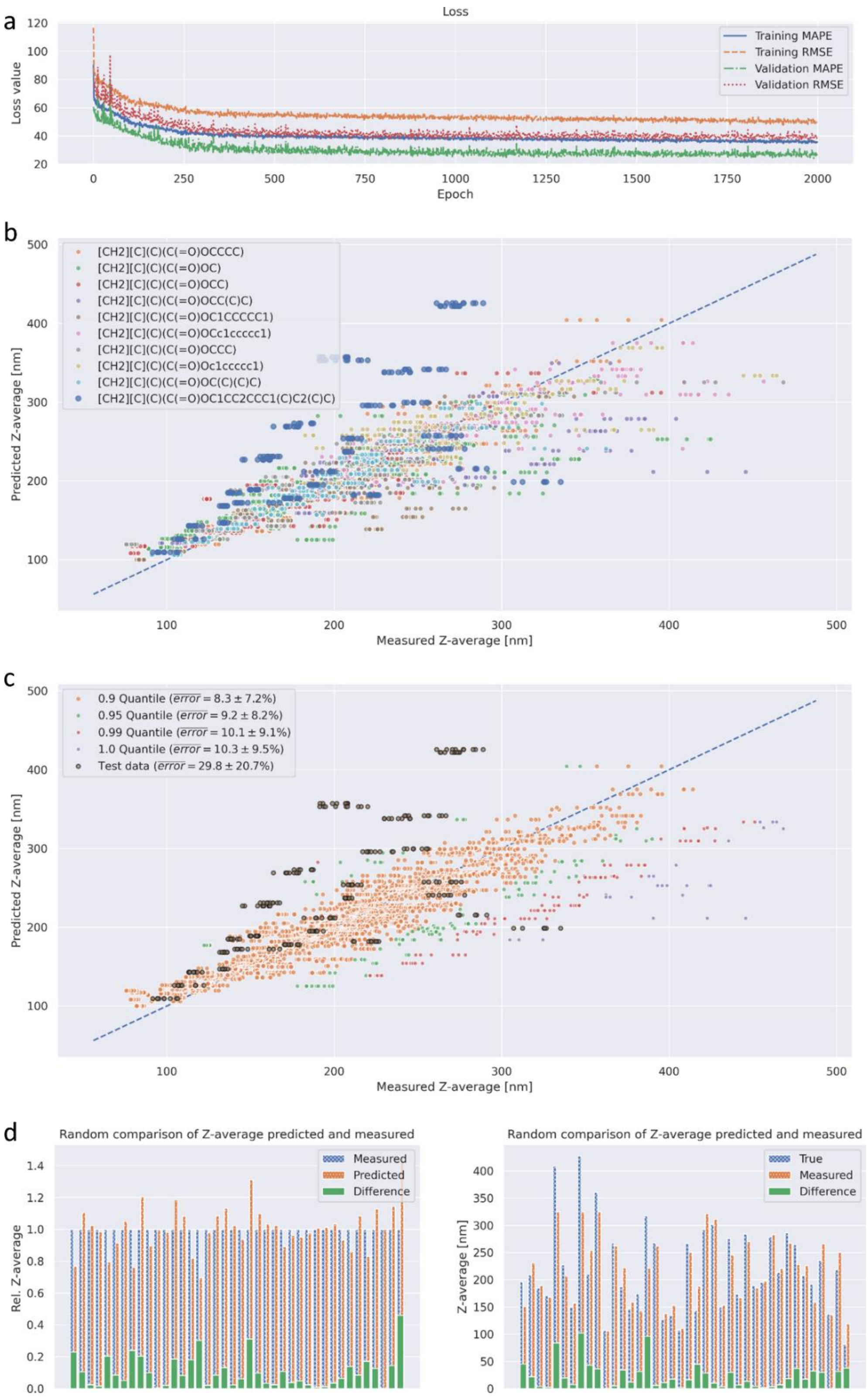
a
Loss
Training MAPE
Training RMSE
Validation MAPE
Validation RMSE
Loss value
Epoch
b
[CH2][C](C)(C(=O)OCCCC)
[CH2][C](C)(C(=O)OC)
[CH2][C](C)(C(=O)OCC)
[CH2][C](C)(C(=O)OCC(C)C)
[CH2][C](C)(C(=O)OC1CCCCC1)
[CH2][C](C)(C(=O)OCc1ccccc1)
[CH2][C](C)(C(=O)OCCC)
[CH2][C](C)(C(=O)Oc1ccccc1)
[CH2][C](C)(C(=O)OC(C)(C)C)
[CH2][C](C)(C(=O)OC1CC2CCC1(C)C2(C)C)
Predicted Z-average [nm]
Measured Z-average [nm]
c
0.9 Quantile ($\overline{error} = 8.3 \pm 7.2\%$)
0.95 Quantile ($\overline{error} = 9.2 \pm 8.2\%$)
0.99 Quantile ($\overline{error} = 10.1 \pm 9.1\%$)
1.0 Quantile ($\overline{error} = 10.3 \pm 9.5\%$)
Test data ($\overline{error} = 29.8 \pm 20.7\%$)
Predicted Z-average [nm]
Measured Z-average [nm]
d
Random comparison of Z-average predicted and measured
Measured
Predicted
Difference
Rel. Z-average
Random comparison of Z-average predicted and measured
True
Measured
Difference
Z-average [nm]

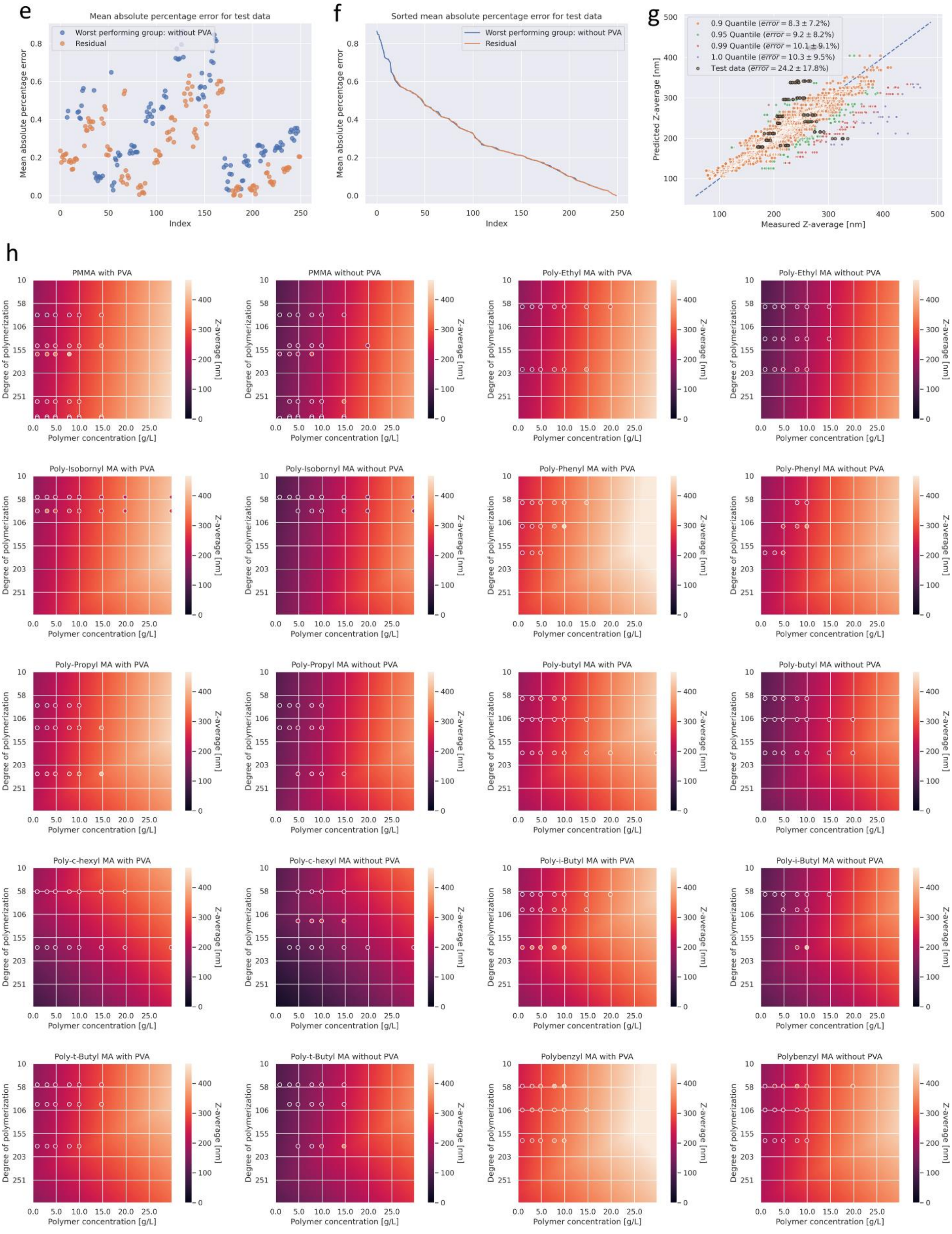
e
Mean absolute percentage error for test data
Worst performing group: without PVA
Residual
Mean absolute percentage error
Index
f
Sorted mean absolute percentage error for test data
Worst performing group: without PVA
Residual
Mean absolute percentage error
Index
g
0.9 Quantile ($\overline{error} = 8.3 \pm 7.2\%$)
0.95 Quantile ($\overline{error} = 9.2 \pm 8.2\%$)
0.99 Quantile ($\overline{error} = 10.1 \pm 9.1\%$)
1.0 Quantile ($\overline{error} = 10.3 \pm 9.5\%$)
Test data ($\overline{error} = 24.2 \pm 17.8\%$)
Predicted Z-average [nm]
Measured Z-average [nm]
h
PMMA with PVA
PMMA without PVA
Poly-Ethyl MA with PVA
Poly-Ethyl MA without PVA
Poly-Isobornyl MA with PVA
Poly-Isobornyl MA without PVA
Poly-Phenyl MA with PVA
Poly-Phenyl MA without PVA
Poly-Propyl MA with PVA
Poly-Propyl MA without PVA
Poly-butyl MA with PVA
Poly-butyl MA without PVA
Poly-c-hexyl MA with PVA
Poly-c-hexyl MA without PVA
Poly-i-Butyl MA with PVA
Poly-i-Butyl MA without PVA
Poly-t-Butyl MA with PVA
Poly-t-Butyl MA without PVA
Polybenzyl MA with PVA
Polybenzyl MA without PVA
Degree of polymerization
Polymer concentration [g/L]
Z-average [nm]

Pretrained model: np_model_11

If all graph convolutional layer are fixed to an output feature size of 5 the resulting network is not able to fully integrate all necessary features into the prediction, which becomes worse.

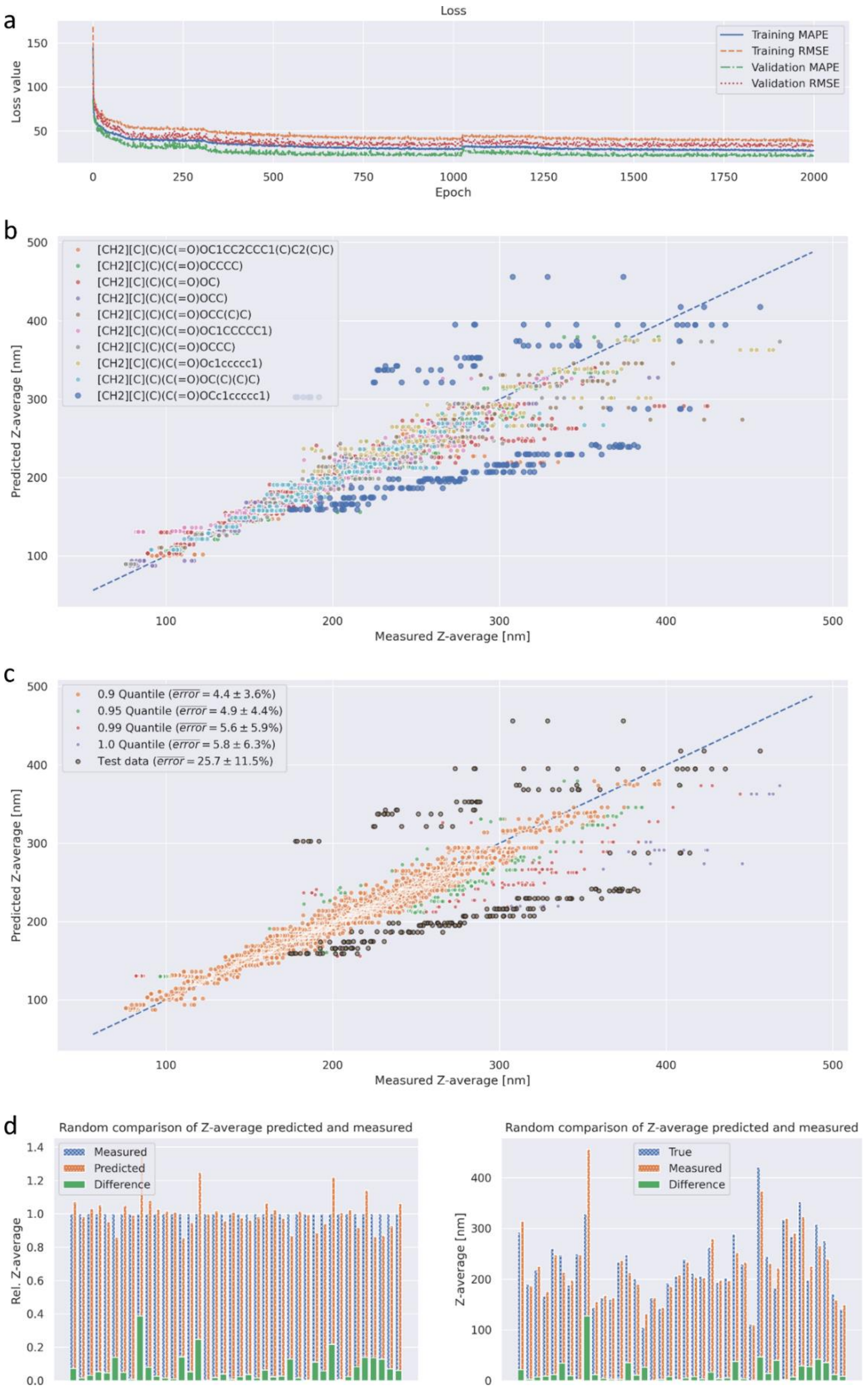

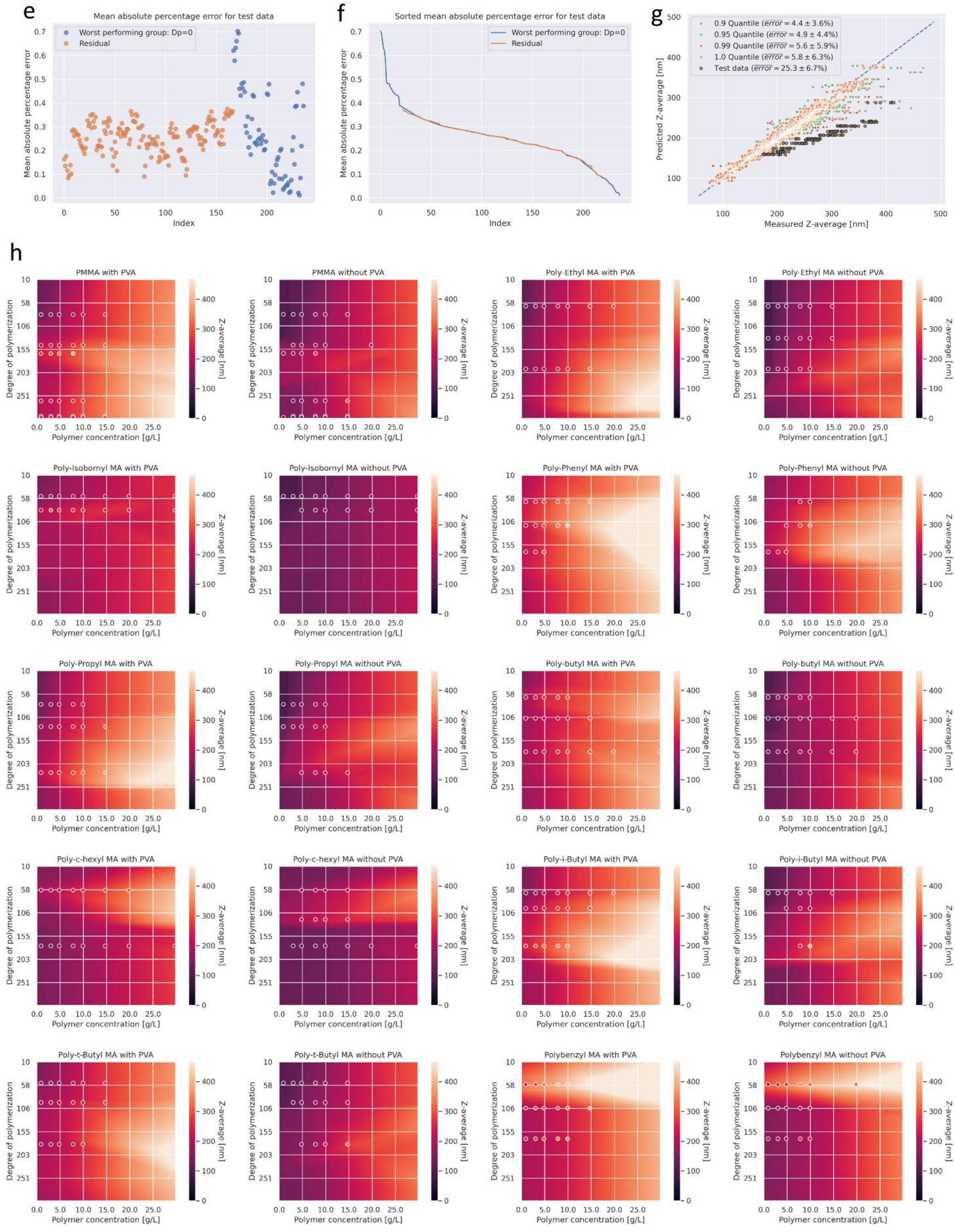
e
Mean absolute percentage error for test data
Worst performing group: Dp=0
Residual
Mean absolute percentage error
Index
f
Sorted mean absolute percentage error for test data
Worst performing group: Dp=0
Residual
Mean absolute percentage error
Index
g
0.9 Quantile ($\overline{error}$ = 4.4 ± 3.6%)
0.95 Quantile ($\overline{error}$ = 4.9 ± 4.4%)
0.99 Quantile ($\overline{error}$ = 5.6 ± 5.9%)
1.0 Quantile ($\overline{error}$ = 5.8 ± 6.3%)
Test data ($\overline{error}$ = 25.3 ± 6.7%)
Predicted Z-average [nm]
Measured Z-average [nm]
h
PMMA with PVA
PMMA without PVA
Poly-Ethyl MA with PVA
Poly-Ethyl MA without PVA
Poly-Isobornyl MA with PVA
Poly-Isobornyl MA without PVA
Poly-Phenyl MA with PVA
Poly-Phenyl MA without PVA
Poly-Propyl MA with PVA
Poly-Propyl MA without PVA
Poly-butyl MA with PVA
Poly-butyl MA without PVA
Poly-c-hexyl MA with PVA
Poly-c-hexyl MA without PVA
Poly-i-Butyl MA with PVA
Poly-i-Butyl MA without PVA
Poly-t-Butyl MA with PVA
Poly-t-Butyl MA without PVA
Polybenzyl MA with PVA
Polybenzyl MA without PVA
Degree of polymerization
Polymer concentration [g/L]
Z-average [nm]

Pretrained model: np_model_12

In this model only two graph convolutional layer were implemented. The prediction results are ok for our structural input but might get worse with increasing structure sizes or more functional units.

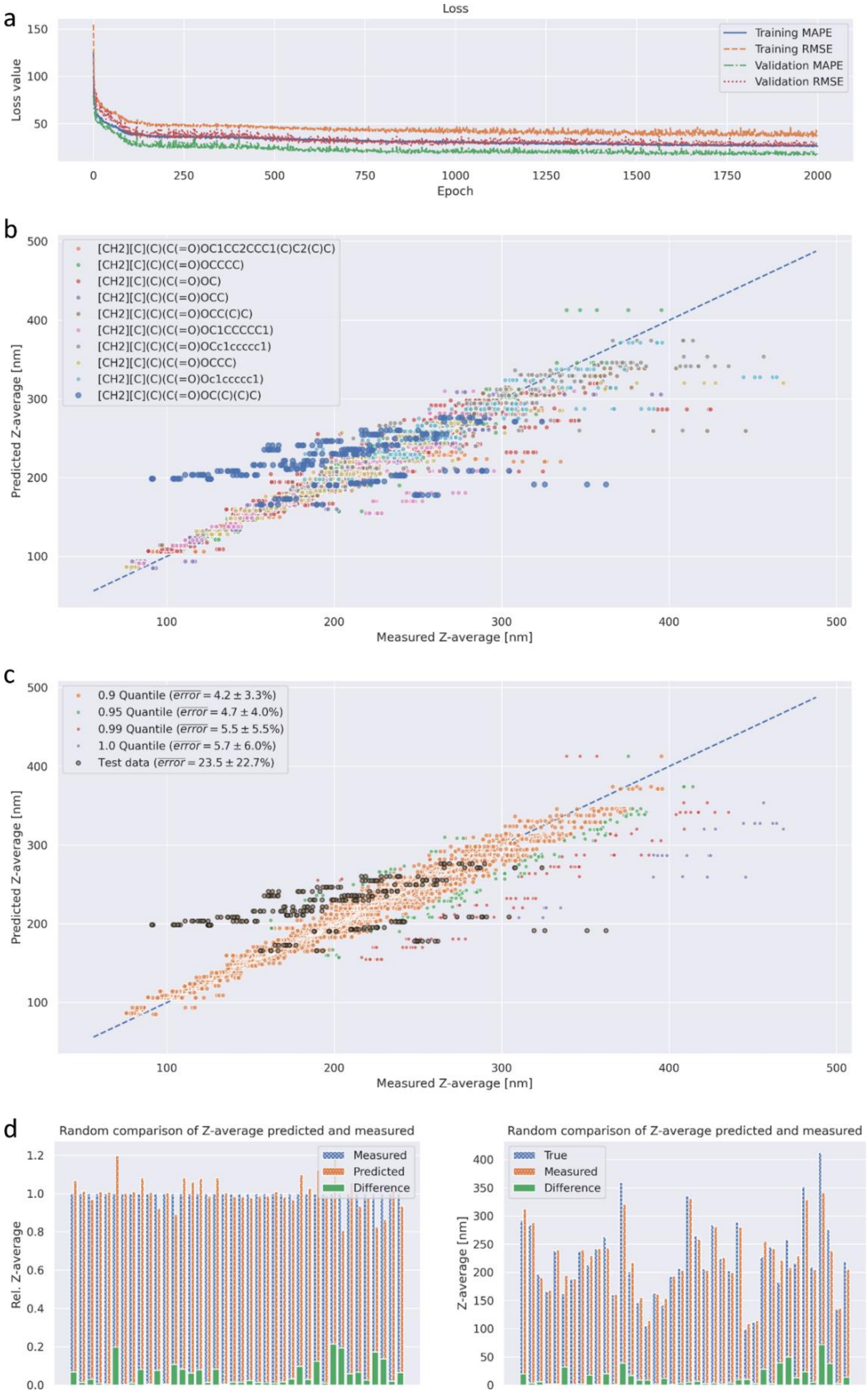

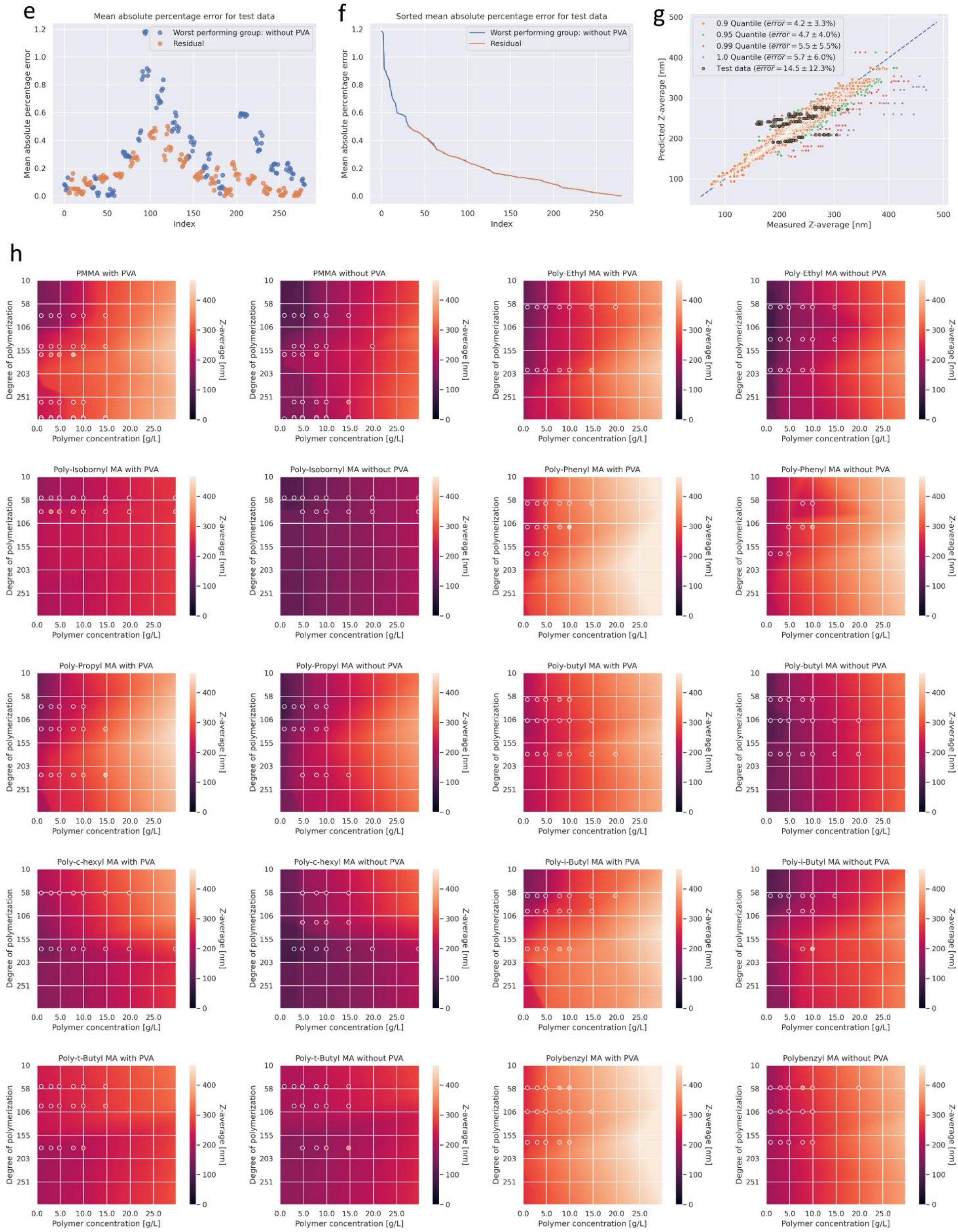
e
Mean absolute percentage error for test data
Worst performing group: without PVA
Residual
Mean absolute percentage error
Index
f
Sorted mean absolute percentage error for test data
Worst performing group: without PVA
Residual
Mean absolute percentage error
Index
g
0.9 Quantile (error = 4.2 ± 3.3%)
0.95 Quantile (error = 4.7 ± 4.0%)
0.99 Quantile (error = 5.5 ± 5.5%)
1.0 Quantile (error = 5.7 ± 6.0%)
Test data (error = 14.5 ± 12.3%)
Predicted Z-average [nm]
Measured Z-average [nm]
h
PMMA with PVA
PMMA without PVA
Poly-Ethyl MA with PVA
Poly-Ethyl MA without PVA
Poly-Isobornyl MA with PVA
Poly-Isobornyl MA without PVA
Poly-Phenyl MA with PVA
Poly-Phenyl MA without PVA
Poly-Propyl MA with PVA
Poly-Propyl MA without PVA
Poly-butyl MA with PVA
Poly-butyl MA without PVA
Poly-c-hexyl MA with PVA
Poly-c-hexyl MA without PVA
Poly-i-Butyl MA with PVA
Poly-i-Butyl MA without PVA
Poly-t-Butyl MA with PVA
Poly-t-Butyl MA without PVA
Polybenzyl MA with PVA
Polybenzyl MA without PVA
Degree of polymerization
Polymer concentration [g/L]
Z-average [nm]

Pretrained model: np_model_13

This model was trained with only the atom symbol as a one-hot encoded feature vector. The results are not as good as the model trained with a larger feature input, but nevertheless achieves good results. A possible explanation is that many features can be indirectly estimated from the type of atom and the connections between them.

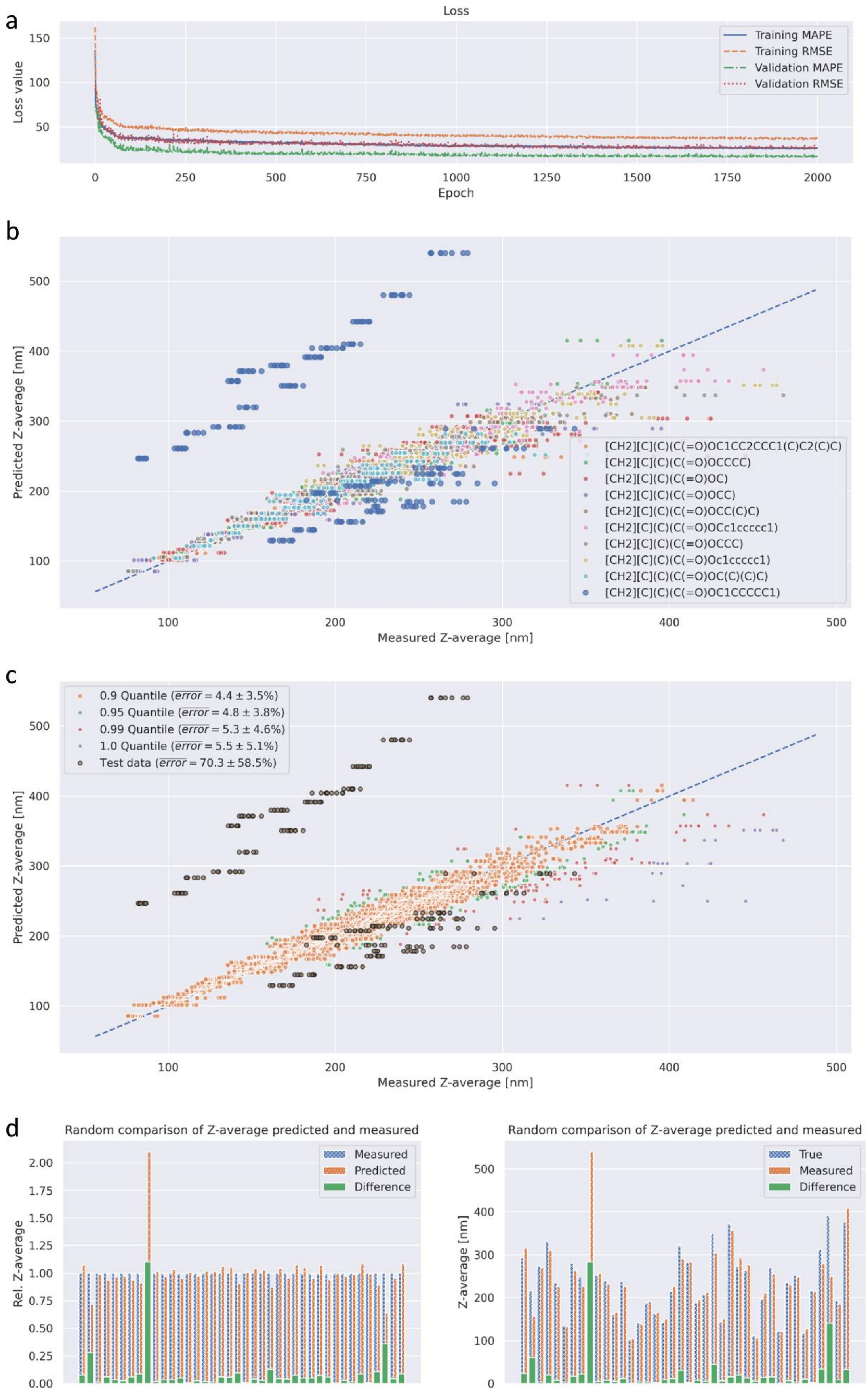
a
Loss
Loss value
Epoch
Training MAPE
Training RMSE
Validation MAPE
Validation RMSE
b
Predicted Z-average [nm]
Measured Z-average [nm]
[CH2][C](C)(C(=O)OC1CC2CCC1(C)C2(C)C)
[CH2][C](C)(C(=O)OCCCC)
[CH2][C](C)(C(=O)OC)
[CH2][C](C)(C(=O)OCC)
[CH2][C](C)(C(=O)OCC(C)C)
[CH2][C](C)(C(=O)OCc1ccccc1)
[CH2][C](C)(C(=O)OCCC)
[CH2][C](C)(C(=O)Oc1ccccc1)
[CH2][C](C)(C(=O)OC(C)(C)C)
[CH2][C](C)(C(=O)OC1CCCCC1)
c
0.9 Quantile ($\overline{error}$ = 4.4 ± 3.5%)
0.95 Quantile ($\overline{error}$ = 4.8 ± 3.8%)
0.99 Quantile ($\overline{error}$ = 5.3 ± 4.6%)
1.0 Quantile ($\overline{error}$ = 5.5 ± 5.1%)
Test data ($\overline{error}$ = 70.3 ± 58.5%)
Predicted Z-average [nm]
Measured Z-average [nm]
d
Random comparison of Z-average predicted and measured
Rel. Z-average
Measured
Predicted
Difference
Random comparison of Z-average predicted and measured
Z-average [nm]
True
Measured
Difference

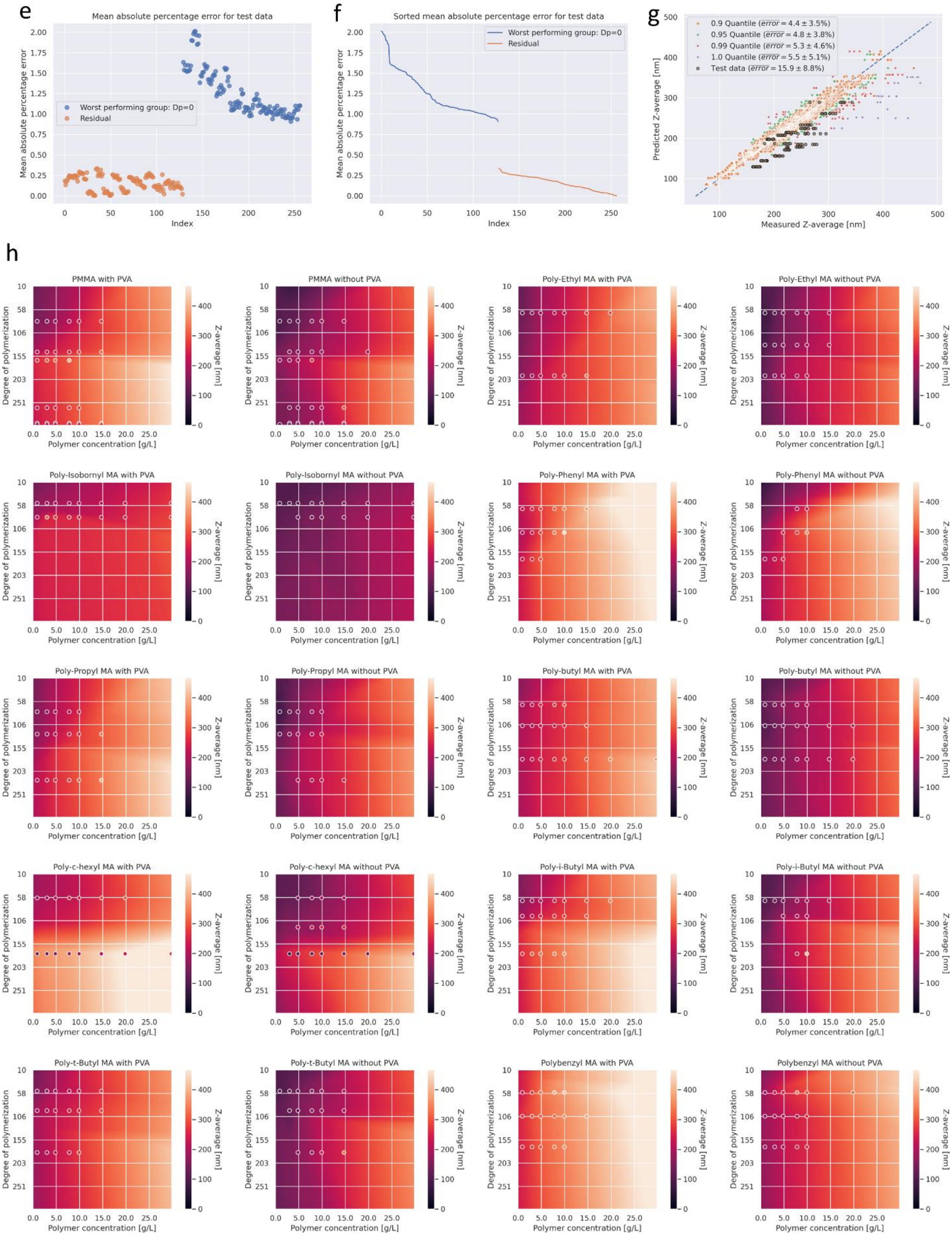
e
Mean absolute percentage error for test data
Mean absolute percentage error
Worst performing group: Dp=0
Residual
Index
f
Sorted mean absolute percentage error for test data
Mean absolute percentage error
Worst performing group: Dp=0
Residual
Index
g
0.9 Quantile ($\overline{error} = 4.4 \pm 3.5\%$)
0.95 Quantile ($\overline{error} = 4.8 \pm 3.8\%$)
0.99 Quantile ($\overline{error} = 5.3 \pm 4.6\%$)
1.0 Quantile ($\overline{error} = 5.5 \pm 5.1\%$)
Test data ($\overline{error} = 15.9 \pm 8.8\%$)
Predicted Z-average [nm]
Measured Z-average [nm]
h
PMMA with PVA
PMMA without PVA
Poly-Ethyl MA with PVA
Poly-Ethyl MA without PVA
Poly-Isobornyl MA with PVA
Poly-Isobornyl MA without PVA
Poly-Phenyl MA with PVA
Poly-Phenyl MA without PVA
Poly-Propyl MA with PVA
Poly-Propyl MA without PVA
Poly-butyl MA with PVA
Poly-butyl MA without PVA
Poly-c-hexyl MA with PVA
Poly-c-hexyl MA without PVA
Poly-i-Butyl MA with PVA
Poly-i-Butyl MA without PVA
Poly-t-Butyl MA with PVA
Poly-t-Butyl MA without PVA
Polybenzyl MA with PVA
Polybenzyl MA without PVA
Degree of polymerization
Polymer concentration [g/L]
Z-average [nm]

Pretrained model: np_model_14

In this model, poly(*tert*-butyl methacrylate) (**P19-P21**) and poly(phenyl methacrylate) (**P28-P30**) were excluded from the training data. As can be seen in e and f, the prediction error for both is completely within the range of the training data.

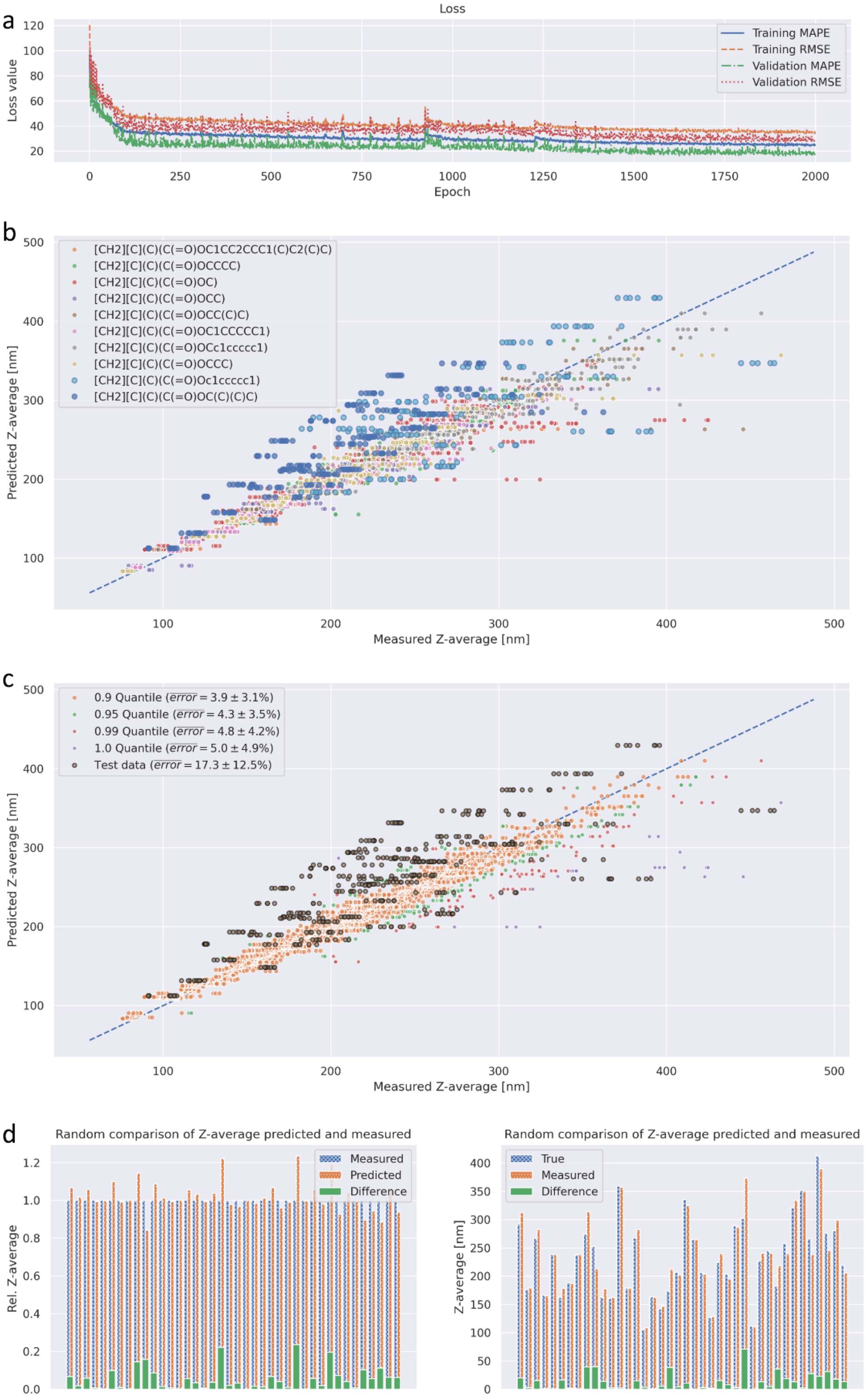
a
Loss
Loss value
Epoch
Training MAPE
Training RMSE
Validation MAPE
Validation RMSE
b
Predicted Z-average [nm]
Measured Z-average [nm]
[CH2][C](C)(C(=O)OC1CC2CCC1(C)C2(C)C)
[CH2][C](C)(C(=O)OCCCC)
[CH2][C](C)(C(=O)OC)
[CH2][C](C)(C(=O)OCC)
[CH2][C](C)(C(=O)OCC(C)C)
[CH2][C](C)(C(=O)OC1CCCCC1)
[CH2][C](C)(C(=O)OCc1ccccc1)
[CH2][C](C)(C(=O)OCCC)
[CH2][C](C)(C(=O)Oc1ccccc1)
[CH2][C](C)(C(=O)OC(C)(C)C)
c
0.9 Quantile (error = 3.9 ± 3.1%)
0.95 Quantile (error = 4.3 ± 3.5%)
0.99 Quantile (error = 4.8 ± 4.2%)
1.0 Quantile (error = 5.0 ± 4.9%)
Test data (error = 17.3 ± 12.5%)
d
Random comparison of Z-average predicted and measured
Rel. Z-average
Measured
Predicted
Difference
Random comparison of Z-average predicted and measured
Z-average [nm]
True
Measured
Difference

e f g

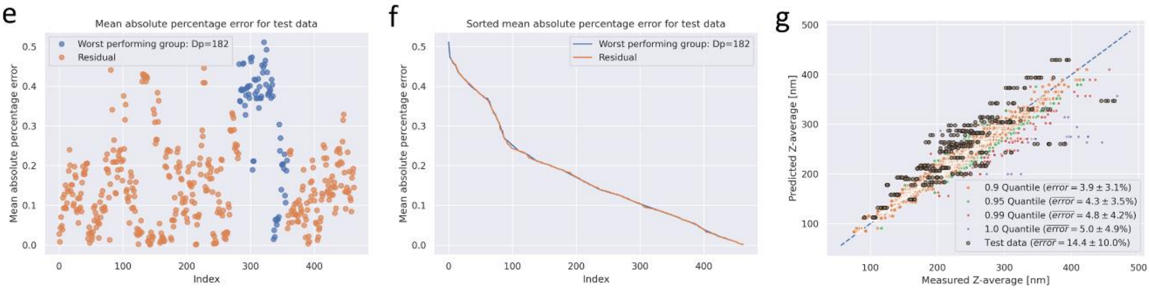
Mean absolute percentage error for test data
Worst performing group: Dp=182
Residual
Mean absolute percentage error
Index
Sorted mean absolute percentage error for test data
Worst performing group: Dp=182
Residual
Mean absolute percentage error
Index
Predicted Z-average [nm]
Measured Z-average [nm]
0.9 Quantile (error = 3.9 ± 3.1%)
0.95 Quantile (error = 4.3 ± 3.5%)
0.99 Quantile (error = 4.8 ± 4.2%)
1.0 Quantile (error = 5.0 ± 4.9%)
Test data (error = 14.4 ± 10.0%)

h

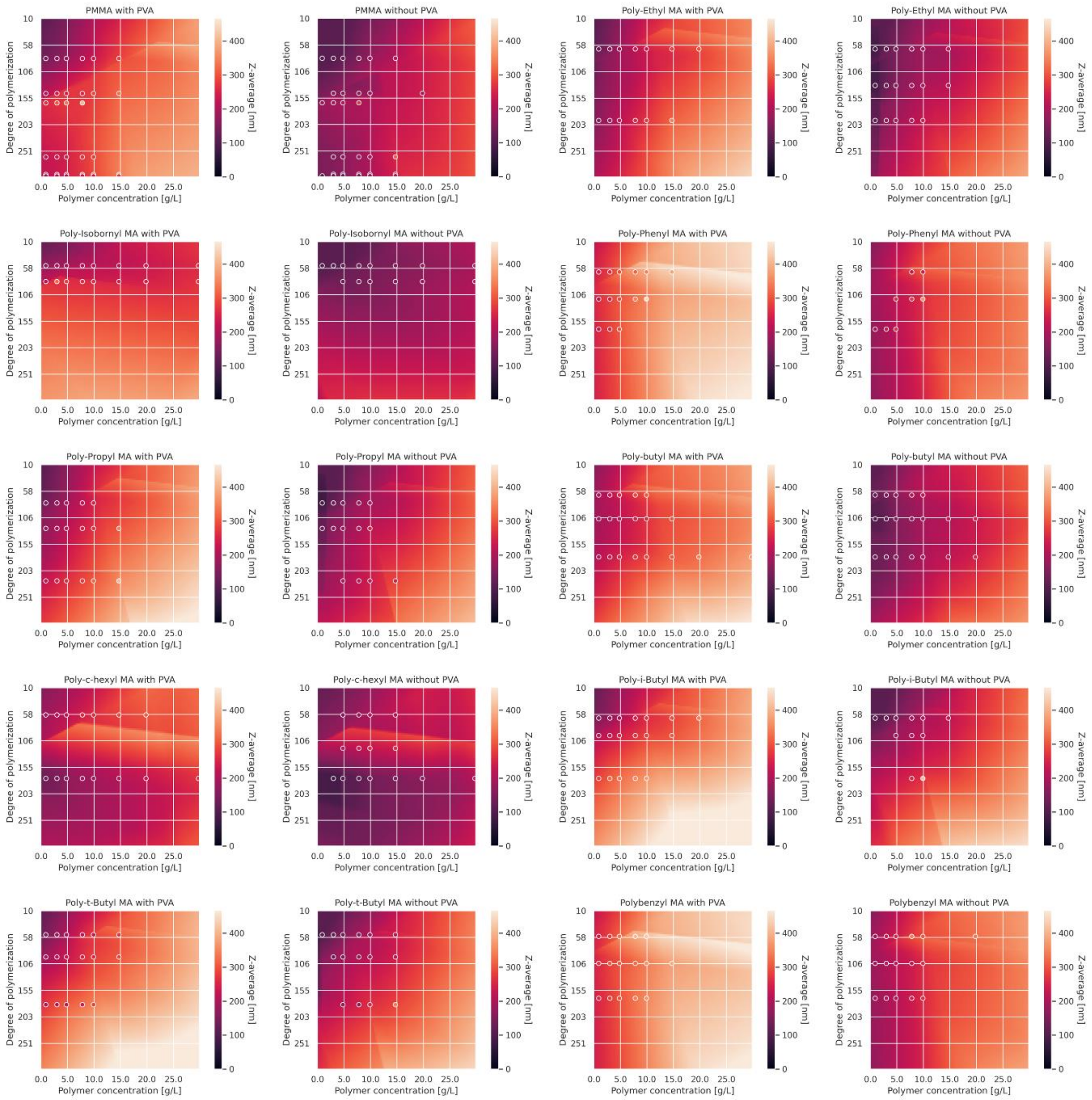
PMMA with PVA
PMMA without PVA
Poly-Ethyl MA with PVA
Poly-Ethyl MA without PVA
Poly-Isobornyl MA with PVA
Poly-Isobornyl MA without PVA
Poly-Phenyl MA with PVA
Poly-Phenyl MA without PVA
Poly-Propyl MA with PVA
Poly-Propyl MA without PVA
Poly-butyl MA with PVA
Poly-butyl MA without PVA
Poly-c-hexyl MA with PVA
Poly-c-hexyl MA without PVA
Poly-i-Butyl MA with PVA
Poly-i-Butyl MA without PVA
Poly-t-Butyl MA with PVA
Poly-t-Butyl MA without PVA
Polybenzyl MA with PVA
Polybenzyl MA without PVA
Degree of polymerization
Polymer concentration [g/L]
Z-average [nm]

References

[1] Directorate-General for Health and Consumers. Scientific basis for the definition of the term “nanomaterial”, 2010.

[2] M. Danaei, M. Dehghankhold, S. Ataei, F. Hasanzadeh Davarani, R. Javanmard, A. Dokhani, S. Khorasani, M. R. Mozafari, *Pharmaceutics* **2018**, *10*, 57.

[3] J. Cheng, B. A. Teply, I. Sherifi, J. Sung, G. Luther, F. X. Gu, E. Levy-Nissenbaum, A. F. Radovic-Moreno, R. Langer, O. C. Farokhzad, *Biomaterials* **2007**, *28*, 869.

[4] D. Weininger, *J. Chem. Inf. Model*. **1988**, *28*, 31.

[5] RDKit: Open-Source Cheminformatics Software, 2020, http://www.rdkit.org.

[6] M. Fey, J. E. Lenssen, (Preprint), arXiv.org, 1903.02428v3, submitted March, 2019.

[7] A. Paszke, S. Gross, F. Massa, A. Lerer, J. Bradbury, G. Chanan, T. Killeen, Z. Lin, N. Gimelshein, L. Antiga, A. Desmaison, A. Köpf, E. Yang, Z. DeVito, M. Raison, A. Tejani, S. Chilamkurthy, B. Steiner, L. Fang, J. Bai, S. Chintala, (Preprint), arXiv.org, 1912.01703v1, submitted December, 2019.

[8] T. Akiba, S. Sano, T. Yanase, T. Ohta, M. Koyama, (Preprint), arXiv.org, 1907.10902v1, submitted July, 2019.
